# *Pseudomonas* Strains Induce Transcriptional and Morphological Changes and Reduce Root Colonization of *Verticillium* spp.

**DOI:** 10.3389/fmicb.2021.652468

**Published:** 2021-05-24

**Authors:** Rebekka Harting, Alexandra Nagel, Kai Nesemann, Annalena M. Höfer, Emmanouil Bastakis, Harald Kusch, Claire E. Stanley, Martina Stöckli, Alexander Kaever, Katharina J. Hoff, Mario Stanke, Andrew J. deMello, Markus Künzler, Cara H. Haney, Susanna A. Braus-Stromeyer, Gerhard H. Braus

**Affiliations:** ^1^Institute of Microbiology and Genetics, Göttingen Center for Molecular Biosciences, Georg-August-Universität Göttingen, Göttingen, Germany; ^2^Department of Medical Informatics, University Medical Center, Georg-August-Universität Göttingen, Göttingen, Germany; ^3^Institute of Chemical and Bioengineering, ETH Zürich, Zurich, Switzerland; ^4^Institute of Microbiology, ETH Zürich, Zurich, Switzerland; ^5^Institute of Mathematics and Computer Science, Universität Greifswald, Greifswald, Germany; ^6^Department of Microbiology and Immunology, The University of British Columbia, Vancouver, BC, Canada

**Keywords:** *Verticillium dahliae*, *Verticillium longisporum*, fluorescent pseudomonads, plant pathogen, fungal growth inhibition, bacterial-fungal interaction, microfluidic device

## Abstract

Phytopathogenic Verticillia cause Verticillium wilt on numerous economically important crops. Plant infection begins at the roots, where the fungus is confronted with rhizosphere inhabiting bacteria. The effects of different fluorescent pseudomonads, including some known biocontrol agents of other plant pathogens, on fungal growth of the haploid *Verticillium dahliae* and/or the amphidiploid *Verticillium longisporum* were compared on pectin-rich medium, in microfluidic interaction channels, allowing visualization of single hyphae, or on *Arabidopsis thaliana* roots. We found that the potential for formation of bacterial lipopeptide syringomycin resulted in stronger growth reduction effects on saprophytic *Aspergillus nidulans* compared to *Verticillium* spp. A more detailed analyses on bacterial-fungal co-cultivation in narrow interaction channels of microfluidic devices revealed that the strongest inhibitory potential was found for *Pseudomonas protegens* CHA0, with its inhibitory potential depending on the presence of the GacS/GacA system controlling several bacterial metabolites. Hyphal tip polarity was altered when *V. longisporum* was confronted with pseudomonads in narrow interaction channels, resulting in a curly morphology instead of straight hyphal tip growth. These results support the hypothesis that the fungus attempts to evade the bacterial confrontation. Alterations due to co-cultivation with bacteria could not only be observed in fungal morphology but also in fungal transcriptome. *P. protegens* CHA0 alters transcriptional profiles of *V. longisporum* during 2 h liquid media co-cultivation in pectin-rich medium. Genes required for degradation of and growth on the carbon source pectin were down-regulated, whereas transcripts involved in redox processes were up-regulated. Thus, the secondary metabolite mediated effect of *Pseudomonas* isolates on *Verticillium* species results in a complex transcriptional response, leading to decreased growth with precautions for self-protection combined with the initiation of a change in fungal growth direction. This interplay of bacterial effects on the pathogen can be beneficial to protect plants from infection, as shown with *A*. *thaliana* root experiments. Treatment of the roots with bacteria prior to infection with *V. dahliae* resulted in a significant reduction of fungal root colonization. Taken together we demonstrate how pseudomonads interfere with the growth of *Verticillium* spp. and show that these bacteria could serve in plant protection.

## Introduction

The genus *Verticillium* comprises soil-borne plant pathogens causing vascular wilt disease in numerous crops. The amphidiploid species *Verticillium longisporum* induces a stem striping disease in rapeseed. In field trials it was found that symptoms occur close to the harvest and might only in some cases result in yield losses (Depotter et al., 2019). Still a recent survey demonstrates that Verticillium stem striping caused by *V. longisporum* belongs to the top ten biotic threats of oilseed rape in Europe (Zheng et al., 2020). In addition, temperature increase caused by global warming might exacerbate the effects of fungal plant disease (Siebold and von Tiedemann, 2013). *V. longisporum* is the result of several independent hybridization events between *Verticillium dahliae* or *V. dahliae*-like species (named D1–D3) as well as a so far unknown species named A1 (Depotter et al., 2016). *V. longisporum* isolates can differ in their pathogenicity toward hosts. Even pathogenic isolates have mechanisms to tame their virulence and limit the damage to the host plant (Harting et al., 2021).

The haploid *V. dahliae* is the economically most important fungus for causing Verticillium wilt, which infects up to several hundred different plants and is further broadening its host range (EFSA Panel on Plant Health [PLH], 2014). Host plants include crops such as strawberry, tomato, olive, lettuce, hops or sunflowers. Verticillium wilt can lead to severe yield losses and control of the fungus with fungicides remains difficult. The impact on agricultural productivity of *Verticillium* species highlights the need for novel efficient but environmentally friendly strategies for effective control of *Verticillium*-induced disease.

Verticillia infect their host plants through the roots, mainly at positions where the endodermis is not fully developed. They first colonize the vascular system and later other tissues of the plant. Once the fungus has entered the plant and reached the xylem vessels, it produces conidia, which are distributed in the plant with the sap stream. At suitable sites, conidia get trapped, germinate and colonize adjacent cells (Fradin and Thomma, 2006). Transport processes in the cell are hindered by increased fungal colonization and potentially also by plant defense mechanisms, as for example the accumulation of phenolic compounds, leading to the development of disease symptoms (Fradin and Thomma, 2006; Kunej et al., 2020). In addition, the fungal infection can also induce cellular changes inside the plant, which allow compensation of the transport processes (Reusche et al., 2014). *Verticillium* spp. produce microsclerotia as long-term resting structures in the dying host, which reach the soil with degrading plant tissue. The melanized cell wall of the resting structures enables the fungus to survive extreme environmental conditions such as radiation, temperature changes and reactive oxygen species but also shelters the fungus against other microorganisms (Belozerskaya et al., 2017; Casadevall et al., 2017; Fan et al., 2020). In one of the next growing seasons, microsclerotia re-establish infections once a susceptible host is present (Wilhelm, 1955; Fradin and Thomma, 2006).

The fungus recognizes appropriate hosts by their root exudates, forms a germ tube and grows toward the root surface (Acharya et al., 2020; Zhang et al., 2020). *Verticillium* spp. have to cope with organisms inhabiting the plant rhizosphere in this hyphal growth phase after microsclerotia germination and prior to root penetration (Deketelaere et al., 2017). *Verticillium* spp. can form hyphopodia, which are swollen hyphal tips, to enter the root. The plant rhizosphere is colonized by a variety of different organisms, including plant-beneficial bacteria. The use of bacteria against plant pathogens is a useful alternative instead of chemical toxins, which improve crop quality and output but can lead to environmental damages (Deketelaere et al., 2017; Syed Ab Rahman et al., 2018). Plants can shape their own root microbiome by attracting certain bacteria (Rudrappa et al., 2008; Berendsen et al., 2018; Huang et al., 2019a), which can not only improve nutrient availability for the plant, but also may act in pest control. *V. dahliae* in turn can shape the soil microbiome in order to enhance infection (Snelders et al., 2020).

Rhizosphere bacteria including the genera *Bacillus* or *Pseudomonas* reduce *Verticillium* spp. growth in co-culture with potential plant growth promoting or biocontrol functions (Hayat et al., 2010; Hollensteiner et al., 2017; Nesemann et al., 2018). In general, bacteria can prevent pathogen infection by a variety of mechanisms. These include the competition for space and/or nutrients, parasitism (mycophagy), enhancement of plant immunity or the production of antimicrobial compounds (Leveau and Preston, 2008; Köhl et al., 2019). Fluorescent pseudomonads secrete various compounds with bioactive properties as potential biocontrol agents of Verticillium wilt (Deketelaere et al., 2017). These include 2,4-diacetylphloroglucinol (DAPG), phenazines, hydrogen cyanide (HCN), pyoluteorin, and cyclic lipodepsipeptides such as syringomycin and syringopeptin (Maurhofer et al., 1994; Haas and Défago, 2005; Berry et al., 2010).

The polyketide DAPG acts on mitochondria, where it eliminates the proton gradient and thereby leads to increased respiration without production of ATP resulting in growth inhibition (Troppens et al., 2013). DAPG and another polyketide, pyoluteorin, enhance their own production through autoregulation and mutually inhibit the synthesis of each other (Schnider-Keel et al., 2000; Brodhagen et al., 2004). Hydrogen cyanide interferes with the mitochondrial respiratory chain through blockage of oxygen reduction (Hamel, 2011). Phenazines are small pigmented metabolites, which lead to formation of reactive oxygen species (ROS), reactive nitrogen species or iron starvation in *Aspergillus fumigatus* (Briard et al., 2015). Closely related *Pseudomonas* strains with beneficial or detrimental effects for *Arabidopsis thaliana* can differ in the genes encoding enzymes required to produce lipopeptides, which are regulated via quorum sensing (Melnyk et al., 2019). Lipopeptides can also have antifungal properties (Sinden et al., 1971; Berry et al., 2010; Meena and Kanwar, 2015; Toral et al., 2018; Desmyttere et al., 2019). Cyclic lipodepsipeptides as for instance syringomycin and syringopeptin can be inserted into plasma membranes to form ion channels resulting in ion leakage and cell death (Hutchison and Gross, 1997). The inhibitory potential of bacteria in fungal co-cultures varies considerably in the presence of different nutrients affecting fungal spore germination or growth (Michelsen and Stougaard, 2012; Nesemann et al., 2018). We could show that *Pseudomonas* isolates from soil are not only able to inhibit plant pathogenic *Verticillium* strains but also moderately affect growth of soil saprophytes of the genus *Aspergillus* (Nesemann et al., 2018). *Aspergillus nidulans* is an important and well-studied model organism and is widely distributed in the soil. Depending on the environmental conditions, it can form mitotic spores, which are distributed by the wind or fruiting bodies as overwintering structures with meiotic spores (Krijgsheld et al., 2013). Another ubiquitous saprophyte is *Aspergillus fumigatus*. Humans are exposed to conidia of this fungus on a regular basis without the development of disease but in immunocompromised patients, the fungus can cause invasive aspergillosis (Kwon-Chung and Sugui, 2013; Shlezinger et al., 2017).

A broader understanding on how *Pseudomonas* spp. act on or interact with fungal pathogens in different environments is needed to explore potential applications in plant protection. The aim of our study was to compare the inhibitory effects of various fluorescent pseudomonads in different artificial co-culture settings to elucidate the antagonistic effects in a controlled environment with a focus on the rapeseed pathogen *V. longisporum*. For individual experiments we also included *V. dahliae*, which is a relative of the *V. longisporum* parental strain as well as the saprophytic soil fungi *A. nidulans* and *A. fumigatus*. *Pseudomonas* isolates with different genomic potentials including the genes to produce lipopeptides and DAPG were examined. Co-cultivation in small interaction channels of microfluidic devices filled with liquid medium, which enabled visualization of growth and polarity effects on single hyphae level was compared to co-cultures on plates. Fungal transcriptome changes were determined in the absence or presence of the strong inhibitor *P. protegens* CHA0 (P_DAPG). The ability of different *Pseudomonas* spp. to interfere with *V. dahliae* plant root colonization as early and important step for host protection was quantified. Our data revealed that different *Pseudomonas* isolates induce morphological and transcriptional alterations when controlling growth of phytopathogenic *Verticillium* spp. and reduce fungal root colonization.

## Materials and Methods

Fungal and bacterial strains used in this study are listed in Supplementary Table 1.

### Cultivation of Fungal Strains

For spore production, *V. dahliae* and *V. longisporum* were grown in liquid simulated xylem medium (SXM), modified according to Neumann and Dobinson (2003) as previously described by Hollensteiner et al. (2017). *A. nidulans* and *A. fumigatus* were grown on solid minimal medium [1% (w/v) glucose, 1 x AspA solution (70 mM NaNO_3_, 7 mM KCl, 11.2 mM KH_2_PO_4_, pH 5.5 with KOH), 1 x (v/v) trace element solution (18 μM FeSO_4_, 174 μM EDTA, 76 μM ZnSO_4_, 178 μM H_3_BO_3_, 25 μM MnCl_2_, 7.1 μM CoCl_2_, 6.4 μM CuSO_4_, 6.2 μM (NH_4_)_6_Mo_7_O_24_; pH 6.5 with KOH) (Hill and Käfer, 2001), 2 mM MgSO_4_, 2% agar] at 37°C in light or darkness, respectively.

### Bacterial Fungal Co-cultivation on Solid Medium

The co-cultivation experiments on solid SXM were performed as previously described in Hollensteiner et al. (2017) and Nesemann et al. (2018). Spores of *Verticillium* spp. were harvested after 7 days of incubation, washed and resuspended in sterile water. After two to three days of incubation *Aspergillus* spp. spores were harvested into a physiological solution (0.96% NaCl, 0.05% Tween 80). The spore concentration was determined with a particle counter (Beckman Coulter, Brea, CA, United States) or in case of *A. fumigatus* with a Thoma cell counting chamber (hemocytometer; Paul Marienfeld GMBH and CO. KG, Lauda-Königshofen, Germany). 1 × 10^5^ spores of the respective fungus were spread on SXM plates. The concentration of bacterial cultures was determined by photometry (Eppendorf BioPhotometer^®^ D30, Hamburg, Germany). 60 μl bacterial culture grown in liquid SXM (OD_600__*nm*_ = 1; 7 × 10^7^ colony-forming bacteria) were added to a hole in the plate center.

The bacteria tested included different strains, which contained or lacked genes involved in lipopeptide biosynthesis. *Pseudomonas brassicacearum* DF41 and *Pseudomonas* sp. N2C3 possess the complete cluster for the production of lipopeptides with homologous genes to *P. syringae* encoding syringomycin and syringopeptin synthetases (Melnyk et al., 2019). The respective genes are absent in the closely related strains N2E2 or WCS365. Different N2C3 deletion strains were examined, including Δ*SYR*, impaired in syringomycin synthesis and Δ*SYP*, missing genes for syringopeptin production. The Δ*SYR*/Δ*SYP* double deletion strain and a strain missing the transcriptional regulator *LUXR* (Δ*LUXR*) are impaired in the synthesis of both lipopeptides.

After 4 days of incubation in the light at 25°C the inhibition radius was measured. Inhibition was defined as area, where no fungal growth was observed. The experiment was conducted in three biological replicates, each consisting of two to four technical replicates.

### Determination of Bacterial Doubling Time

For comparison of doubling times, bacterial strains were inoculated in 50 ml SXM to an OD_600_ of 0.2 and cultures were incubated at 25°C shaking for 12 h. OD_600_ was measured directly after inoculation and then every 2 h. Growth curves were plotted and the respective doubling time (Td) was calculated based on the exponential growth phase of the individual bacterial strains (Allen and Waclaw, 2019). The experiment was conducted in three biological replicates.

### Bacterial Fungal Co-cultivation in Microfluidic Devices

*Verticillium longisporum* was inoculated with a piece of agar from a fungal colony at one side of the device (Stanley et al., 2014), which was filled with liquid pectin-rich SXM. Then, the device was incubated at 25°C, which allowed individual hyphae of the fungus to cross a small connecting bridge (10 μm width) and enter the interaction channels (110 μm width, 6650 μm length). Once the fungus entered these interaction channels, bacterial cultures (40 μl, OD_600__*nm*_ = 1; 5 × 10^7^ CFU) were added at the opposite end and the device was further incubated at 25°C. The number of devices used for each experiment is indicated in the respective figure legend. Fungal growth was evaluated once the fungus of the non-treated control reached the end of the channels using a fluorescence microscope (Zeiss Axio Observer Z.1 system (Carl Zeiss AG, Germany) with Laser Lunch System (Model 3iL32, Intelligent Imaging Innovations Inc., Colorado, United States), Zeiss Plan-Apochromat 40×/1,4 oil objective, QuantEM:512SC camera (Photometrics, AZ, United States) and the Slide Book 5.0 imaging software (Intelligent Imaging Innovations Inc.) with an exposure time of 150 ms for GFP).

### Sample Preparation and Evaluation of Transcriptomic Data

1350 ml liquid SXM were inoculated with 9 × 10^6^ spores of *V. longisporum* Vl43 and incubated for five days at 25°C under constant agitation at 150 rpm. After slight sedimentation, 350 ml supernatant were discarded. The remaining culture was equally separated to eight cultures of 125 ml each. *P. protegens* P_DAPG (CHA0) cultures were also grown in SXM at 25°C and 150 rpm to an OD_600__*nm*_ = 1. After centrifugation at 5000 *g* bacteria were resuspended in fresh SXM to an OD_600__*nm*_ = 3. To six of the fungal cultures, 25 ml bacterial culture were added resulting in a co-culture with an OD_600__*nm*_ = 0.5. Two fungal cultures mixed with 25 ml SXM served as control. All cultures were further incubated under agitation at 25°C. After 60, 120, and 180 min, respectively two co-cultures were filtered through Miracloth (EMD Millipore Corp., Billerica, MA, United States) and the mycelium remaining in the filter was frozen in liquid nitrogen. The control cultures without bacteria were harvested after 120 min, thus further analysis was conducted for data obtained from the 120 min time point. RNA extraction was performed using the RNeasy Plant Mini Kit (Qiagen, Hilden, Germany). RNA sequencing was conducted by GATC Biotech AG (Konstanz, Germany). By selecting the poly-A^+^-part of the eukaryotic mRNA, only fungal RNA was sequenced. The sequences were released at NCBI under the Accession number SRP068348^1^.

The produced RNAseq data were analyzed using the GALAXY platform (Afgan et al., 2018) maintained by the GWDG (Gesellschaft für wissenschaftliche Datenverarbeitung mbH Göttingen). In short, the raw reads from the sequencing were mapped against the *Verticillium longisporum* genome (GCA_001268145.1, (Fogelqvist et al., 2018)) using Bowtie 2 (Langmead and Salzberg, 2012). The produced alignment files were then used together with the GFF file for *V. longisporum* (GCA_001268145.1, (Fogelqvist et al., 2018) in the tool HTSeq (Anders et al., 2015) to calculate the number of mapped reads per feature and generate the corresponding matrices, required for the subsequent analysis. The output files from HTSeq were then used for the identification of statistically significant differentially expressed genes with the tool DESeq2 (Love et al., 2014). For the produced data cut off criteria were applied regarding the *p*-value (*p* < 0.0001) and Log_2_-fold-change. Transcripts with a Log_2_-fold-change > 2 were regarded as most up-regulated and those with a Log_2_-fold-change < −2 were regarded as most down-regulated (Supplementary Tables 2, 3). The GO enrichment analysis was performed separately for the down-regulated and up-regulated genes using the g:Profiler (Raudvere et al., 2019). Genes within significantly enriched GO categories (Supplementary Tables 4, 5) were further investigated using EnsemblFungi (Yates et al., 2020) with embedded InterPro (Mitchell et al., 2019). Some predicted proteins were additionally compared by BLAST with NCBI (Agarwala et al., 2018) and AspGD (Cerqueira et al., 2014). Cellular localization of selected candidates was predicted using DeepLoc 1.0 (Almagro Armenteros et al., 2017).

### *Arabidopsis thaliana* Root Infection With *V. dahliae* in Presence of Bacteria

*Arabidopsis thaliana* (Col-0, N1902; Nottingham Arabidopsis Stock Centre) seeds were surface sterilized and grown as described by Bui et al. (2019) and Harting et al. (2020) with following modifications.

*Escherichia coli* and *Pseudomonas* strains were inoculated in Lysogeny broth (LB; Bertani, 1951) and grown over the day at 30°C, under constant agitation. From this culture, 50 ml LB were inoculated to an OD_600__*nm*_ = 0.00001 or in case of the *P. synxantha* or *P. brassicacearum* isolates to an OD_600__*nm*_ = 0.001. After overnight incubation at 30°C or 37°C (*E. coli*) the bacterial cultures were centrifuged at room temperature, with 2500 rpm for 5 min. The supernatant was discarded and the cells were washed in dH_2_O twice. Eventually the cells were resuspended in dH_2_O and adjusted to an OD_600__*nm*_ = 0.001 in dH2O.

20-day-old *A. thaliana* seedlings were transferred to H_2_O agarose plates (1% agarose (Biozym LE Agarose) in dH_2_O) and roots were treated with the respective bacterial suspension (OD_600__*nm*_ = 0.001) or dH_2_O as a control. For that, 50 μl were applied to the hypocotyl of an individual *A. thaliana* Col-0 plant. Bacteria were spread as the 50 μl drop ran down the root. The plants were further incubated under long day conditions. On the next day all plants were shifted to fresh H_2_O agarose plates to only keep bacteria on the root. The following day, the plants were infected with *GFP*-expressing *V. dahliae* JR2 (Tran et al., 2014) via root dipping in 50 ml of a 1 × 10^5^ spores per ml suspension as described by Bui et al. (2019). Five days after infection, roots were stained using 0.0025% propidium iodide/0.0005% silwet staining solution and colonization of the roots was analyzed using fluorescence microscopy (see section “Bacterial Fungal Co-cultivation in Microfluidic Devices”; EC Plan-Neofluar 20×/0.50 objective and Slide Book 6.0 imaging software were used). For one biological replicate two independent roots were analyzed per treatment. Per root, 10–15 stacks consisting of several individual pictures were acquired to image the different layers of the complete root. Positions were chosen randomly along the root. From each stack, a projection was created. The experiment was performed in three or four replicates (without bacteria, with the N2C3 wild type and respective mutants). Fluorescence quantification was performed using mask statistics feature of Slidebook 6.0 software package (Intelligent Imaging Innovations). Mean GFP intensity was normalized to mean root area. Mean values of biological replicates were normalized to the control of only fungus without bacteria.

## Results

### *Pseudomonas* spp. Specifically Inhibit Different Fungi in Growth on Plate

We investigated whether soil inhabiting pseudomonads with or without the ability to form the lipopetides syringomycin and/or syringopeptin have effects on growth of the plant pathogenic fungus *Verticillium longisporum*. For comparison, the haploid parental relative *V. dahliae* was included. Cultivations of only fungi or co-cultivation with *E. coli* were used as controls. As culture medium simulated xylem medium (SXM) was used, which was initially developed to mimic the conditions inside the plant. SXM mainly contains pectin, which is found in plant debris in the soil and is significantly different from plant xylem sap, which is a nutrient-poor unbalanced medium (Singh et al., 2010). *V. longisporum* is able to distinguish between these two environments, which is supported by two different secretomes, which are expressed by the fungus when grown in these two media (Leonard et al., 2020).

**FIGURE 1:**
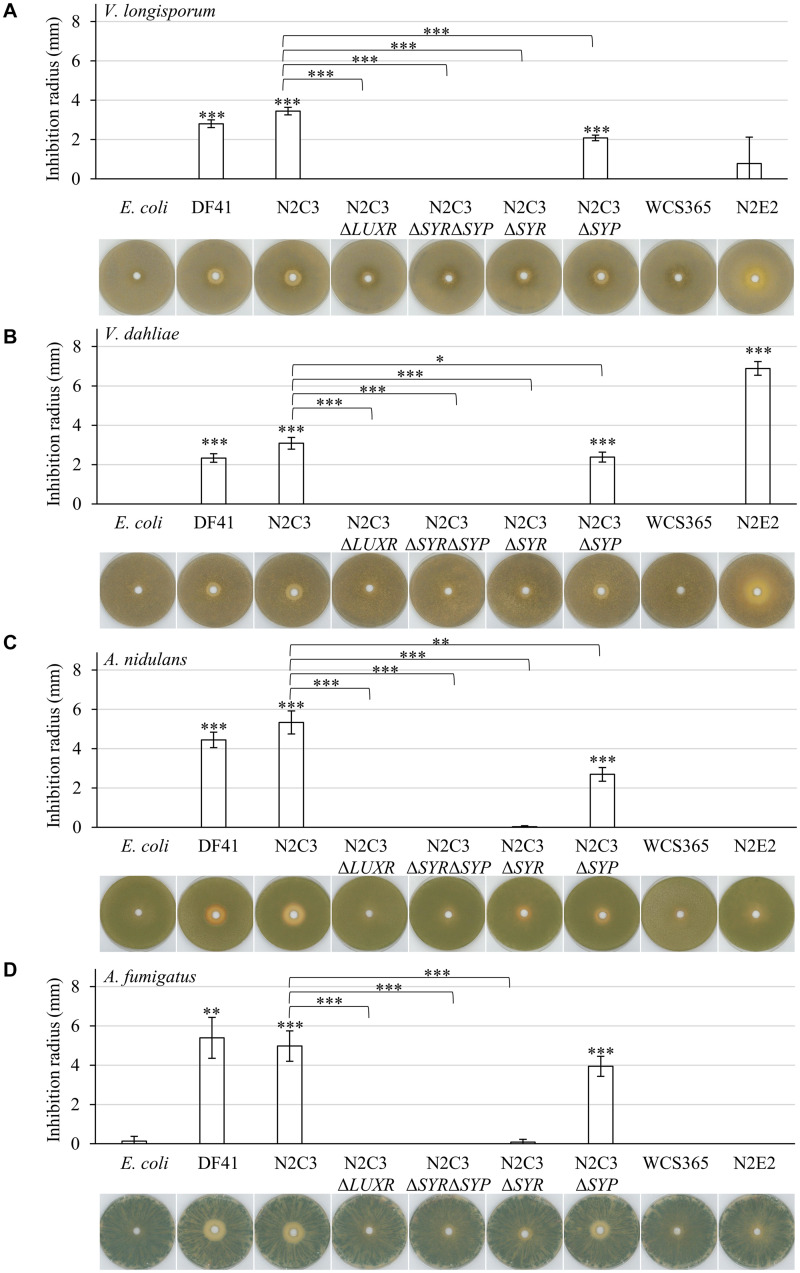
Lipopeptide producing fluorescent pseudomonads inhibit plant pathogenic *Verticillium* spp. as well as saprophytic *Aspergillus* spp. Co-cultivation of 1 × 10^5^ fungal spores with 7 × 10^7^ colony-forming bacteria on pectin-rich simulated xylem medium. Plates were incubated in the light for four days at 25°C. The following bacterial isolates were used: *Pseudomonas brassicacearum* DF41 and *Pseudomonas* sp. N2C3 (cluster for the production of lipopeptides present), N2C3 deletion strains Δ*LUXR* (neither syringomycin nor syringopeptin synthesis), Δ*SYR*Δ*SYP* (neither syringomycin nor syringopeptin synthesis), Δ*SYR* (no syringomycin synthesis), Δ*SYP* (no syringopeptin synthesis), WCS365 and N2E2 (both no cluster for lipopeptide synthesis), *E. coli* as control. Each cultivation was performed in three biological replicates with two to four technical replicates each. Bars represent the mean of biological replicates with standard deviation. Statistical differences were calculated with two-tailed Student’s T-test (^∗^*p* < 0.05, ^∗∗^*p* < 0.01, ^∗∗∗^*p* < 0.001). Differences to *E. coli* control are indicated directly on top of the bars, differences between bacterial wild types and respective mutant strains are indicated with connecting lines. Representative plates are shown beneath the diagrams. Only zones without any fungal growth were measured. Co-cultivation was performed with the rapeseed pathogen *V. longisporum* Vl43 **(A)**, the tomato pathogen *V. dahliae* JR2 **(B)**, the saprophyte *A. nidulans* A4 **(C)** and the opportunistic human pathogen *A. fumigatus* AfS35 **(D)**. Fungal growth inhibition by the tested *Pseudomonas* isolates was increased toward *Aspergillus* spp. compared to *Verticillium* spp. The antagonistic effect of N2C3 was more dependent on syringomycin than on syringopeptin.

Both bacterial strains with lipopeptide clusters (DF41 and N2C3) were able to inhibit growth of *Verticillium* spp. (Figures 1A,B). This effect depends on the presence of the gene for the transcriptional regulator LuxR or genes involved in synthesis of syringomycin (Δ*LUXR*, Δ*SYR*, Δ*SYR*/Δ*SYP*). Absence of only the gene for syringopeptin synthesis (Δ*SYP*) decreased the antagonistic effect toward *Verticillium* spp. compared to the respective wild type N2C3. The closely related strains WCS365 and N2E2, which do not encode genes for lipopeptide synthesis, showed a dissimilar effect on *Verticillium* fungi. Whereas the strain WCS365 was not able to inhibit growth of both isolates, N2E2 showed a strong antagonistic effect against *V. dahliae*, but no significant growth inhibition of *V. longisporum*. This suggests that the bacterial isolate N2E2 produces other bioactive substances that are specifically harmful to *V. dahliae*.

Both *Verticillium* species are hemibiotrophic pathogens with only a short saprophytic phase. We further examined bacterial antagonism on specialized saprophytes, as the soil fungus *Aspergillus nidulans* or *A. fumigatus*, which can occasionally be an opportunistic human pathogen in immunocompromised individuals. The overall effect of lipopeptide producing strains on growth of *Aspergillus* spp. is more pronounced than the antagonistic activity toward *Verticillium* spp. (Figures 1C,D; Supplementary Table 6). Syringomycin contributes more to fungal growth inhibition than syringopeptin. The two strains lacking essential genes for lipopeptide synthesis, WCS365 and N2E2, were not able to affect hyphal growth of the two *Aspergillus* isolates.

As control we compared doubling times of the different bacterial wild type isolates as well as respective mutant strains to exclude a fitness effect. Growth of the isolates DF41, N2C3 and N2E2 was similar, whereas the isolate WCS365 even grew significantly better under the tested conditions (Supplementary Figure 1). Comparison of the isolate N2C3 and the mutant strains impaired in lipopeptide production shows similar doubling times without significant differences (Supplementary Figure 1). This suggests that the observed changes in fungal inhibition are not the result of differences in bacterial growth.

Taken together, presence of the genes required for syringomycin production is a prerequisite to inhibit growth of plant pathogenic *Verticillium* species and to a greater extend also the saprophyte *A. nidulans* and the opportunistic human pathogen *A. fumigatus*.

### *V. longisporum* Polar Hyphal Growth Is Reduced by Fluorescent Pseudomonads

Co-cultivation of *Verticillium* hyphae with *Pseudomonas* spp. might affect growth differently when agar plates are compared to spatially restricted device conditions. Small interaction channels of microfluidic devices (Stanley et al., 2014) allowed confrontation assays and microscopic analysis of single hyphae. Bacterial isolates from the rhizosphere were used for these experiments. The analyzed strains exhibited a stronger antagonistic potential toward *V. longisporum* compared to the lipopeptide producing bacteria (Nesemann et al., 2018). The growth inhibition potential of the rhizosphere bacteria on plate was dependent on the medium and the genomic potential to produce different metabolites *Pseudomonas fluorescens* DSM8569 (P_rhizo) was isolated from the rhizosphere of rapeseed (Berg and Ballin, 1994). P_rhizo lacks the entire cluster for the production of phenazines and also part of the cluster for 2-4-diacetylphloroglucinol (DAPG) formation (Nesemann et al., 2015a, 2018). *Pseudomonas synxantha* 2-79 (P_phen), which was isolated from wheat rhizosphere (Weller and Cook, 1983), has the potential to produce phenazines (Nesemann et al., 2015b, 2018). *Pseudomonas protegens* CHA0 (P_DAPG) was isolated from the rhizosphere of tobacco (Stutz et al., 1986). P_DAPG lacks the genes for phenazine synthesis, but has genes for DAPG, pyoluteorin and HCN synthesis and also the GacS/GacA control system for the production of several metabolites (Jousset et al., 2014). The production of DAPG, HCN and pyoluteorin in the P_DAPG strain was also experimentally verified (Laville et al., 1992; Schnider-Keel et al., 2000).

Growth of single hyphae of the rapeseed pathogen *V. longisporum* Vl43 in presence of *Pseudomonas* isolates P_rhizo, P_phen, P_DAPG and mutant strains were monitored in microfluidic interaction devices depicted in Supplementary Figure 2 (Stanley et al., 2014). Mycelium of the fungal strain ectopically overexpressing *GFP* was inoculated at one side of the interaction device. Hyphae were grown into the device filled with pectin-rich simulated xylem medium (SXM). As the hyphae entered the interaction channels of the device, bacterial culture was added at the opposite side for co-cultivation. Fungal growth was quantified by measuring how far hyphae grew through the device relative to the control (100%) using the scale of the device.

**FIGURE 2:**
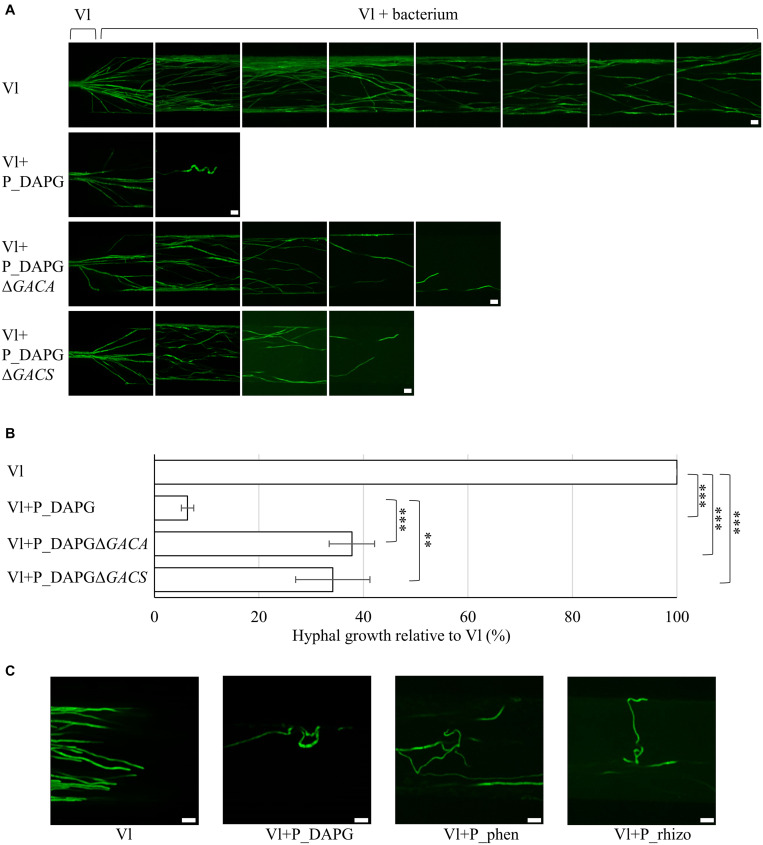
Fluorescent pseudomonads inhibit fungal growth and induce polarity changes in microfluidic devices. Co-cultivation was performed in liquid pectin-rich simulated xylem medium. *V. longisporum* Vl43 producing high amounts of GFP (Vl) was inoculated at one side of the device with an agar block containing hyphae. The device was incubated at 25°C until hyphae entered the microchannels. As soon as the fungal hyphae reached the beginning of the interaction channels fluorescent pseudomonads were inoculated at the opposite end of the channel. Bacteria spread throughout the device. **(A)** Representative micrographs of a *V. longisporum* strain producing high amounts of GFP without (Vl) and with fluorescent bacteria with genes for DAPG synthesis (Vl + P_DAPG) and respective mutant strains, which lack the GacS/GacA two-component control system for the synthesis of various metabolites (Vl + P_DAPGΔ*GACS*, Vl + P_DAPGΔ*GACA*). Images were acquired from three devices with 28 interaction channels each. Fungal growth inhibition partially depends on the presence of GacS/GacA. Scale bars: 20 μm. **(B)** Quantification of fungal growth in devices relative to *V. longisporum* growth without bacteria (Vl), which was set to 100% for each device. Bars represent the mean of three devices and error bars the respective standard deviation. Statistical significance was calculated with two-tailed Student’s T-test (^∗∗^*p* < 0.01, ^∗∗∗^*p* < 0.001). **(C)** Representative micrographs of a *V. longisporum* strain producing high amounts of GFP without (Vl) and with fluorescent bacteria with genes for DAPG (Vl + P_DAPG) or phenazines (Vl + P_phen) synthesis and a bacterium isolated from the rhizosphere of rapeseed (Vl + P_rhizo). Polar growth of *V. longisporum* is altered when cultivated together with different bacterial isolates resulting in a curly phenotype. Two (Vl + P_phen and Vl + P_rhizo) or three (Vl + P_DAPG) devices were evaluated. Scale bars: 20 μm.

Specific associations of bacteria with fungal hyphae were never observed during these experiments. Bacteria were instead distributed throughout the whole medium. Co-cultivation with the bacteria resulted in measurable reductions of fungal growth compared to the non-treated control (Figures 2A,B) or co-cultivation with *E. coli* (Supplementary Figures 3A,B). Incubation with P_rhizo decreased fungal growth to approximately 42% (Supplementary Figures 3A,B). *V. longisporum* hyphal growth was reduced from 100% to approximately 6% when co-cultivated with P_DAPG (Figures 2A,B). This was the strongest bacterial effect observed. The doubling time of P_DAPG is decreased compared to the other rhizosphere isolates. However, the overall difference in doubling times is rather small, therefore the impact of these differences might be neglectable (Supplementary Figures 1A,B). Incubation with P_phen decreased fungal growth to approximately 30%. A mutant bacterial strain lacking parts of the phenazine metabolite gene cluster improved fungal growth in comparison to P_phen (decreased to only approximately 57% compared to non-treated control; Supplementary Figure 3), although no significant differences in the doubling time were observed (Supplementary Figure 3C). This suggests that the effect of P_phen on fungal growth is partially mediated by phenazines.

Further analysis focused on *Pseudomona*s P_DAPG, which caused the strongest inhibitory effect of the three bacterial wild type strains on the fungus. Several bacterial P_DAPG mutant derivatives were analyzed, which are impaired in the production of different metabolites. The focus was on strains, which did not show any statistically significant difference in the doubling times compared to wild type (Supplementary Figure 4). Deletion of a structural gene for a DAPG biosynthetic enzyme (P_DAPGΔ*PHLA*) resulted in a strain without DAPG but increased pyoluteorin production (Schnider-Keel et al., 2000). Lack of the gene for the repressor of the DAPG operon (P_DAPGΔ*PHLF*) resulted in increased DAPG production (Schnider-Keel et al., 2000). P_DAPGΔ*PHLA* and P_DAPGΔ*PHLF* did not lead to strong changes in fungal growth inhibition compared to P_DAPG. Deletion of genes for HCN (P_DAPGΔ*HCN* and P_DAPGΔ*ANR*) resulted in not detectable or strongly reduced HCN production (Laville et al., 1998). In the strain P_DAPGΔ*PLT* no pyoluteorin was produced (Maurhofer et al., 1994). Both, HCN and pyoluteorin, are dispensable for the inhibitory potential of the bacteria (Supplementary Figure 5). Deletion strains of genes encoding GacS or GacA (P_DAPGΔ*GACS* and P_DAPGΔ*GACA*), which function together in a two-component system to control production of multiple metabolites, did not produce DAPG, HCN or pyoluteorin (Laville et al., 1992; Zuber et al., 2003). Co-cultivation with both strains caused significantly less fungal repression (38% and 34% relative to 100% fungal growth without bacteria or reduction to 6% in presence of P_DAPG; Figures 2A,B). This suggests an important role of the GacS/GacA two-component signal transduction pathway in the bacterial-fungal interaction. As there was still an inhibitory effect observable these results further indicate that there are also GacS/GacA independent inhibitory effects.

An interesting observation of the microscopy studies with microfluidic interaction devices was that polarity of single hyphal growth was disturbed when the fungus was confronted with either of the bacterial isolates P_DAPG, P_phen and P_rhizo, respectively (Figure 2C). The untreated *V. longisporum* control (Vl) grew in a polar manner, whereas many hyphal tips started to redirect growth and showed an overall curly phenotype when the bacterial antagonist was added to the device. This curly phenotype was less pronounced in co-cultivation with bacterial mutant strains deficient in GacS/GacA system (P_DAPGΔ*GACS*, P_DAPGΔ*GACA*, Figure 2A) or phenazine production (P_phenΔ*PHZ;* Supplementary Figure 3A), which is in line with our observations regarding fungal growth inhibition.

In summary, these data suggest that fluorescent *Pseudomonas* strains induce polarity changes of the fungus when space is limited. Bacteria are able to significantly decrease growth of single fungal hyphae. This effect on growth is most severe when a bacterial strain is used with a broad potential for the synthesis of different metabolites, which is dependent on the GacS/GacA system that controls the production of several metabolites. Such strong morphological growth defects correlate most likely with significant transcriptomic changes in fungal cells.

### *P. protegens* P_DAPG Induces Transcriptional Changes in *V. longisporum* for Substrate Utilization and Oxidation-Reduction Processes

RNA sequencing was applied to analyze how co-cultivation induces changes in the fungal transcriptome. Pre-grown fungal cultures were co-cultivated with the bacterium P_DAPG (CHA0), which showed the strongest inhibition potential in previous experiments, in liquid SXM in submerged cultures for 120 min. RNAs were extracted and sequenced. Fungal culture without bacteria served as control. Reads were mapped onto the genome of *V. longisporum* VL1 (Fogelqvist et al., 2018) and data were further processed as described in the Materials and Methods section.

We found a total of 2151 transcripts which were significantly differentially expressed (*p*-value < 0.0001). 1407 genes were found to be up-regulated in the presence of *P. protegens* P_DAPG (Log_2_-fold-change > 2; Supplementary Table 2). 744 transcripts were down-regulated when the fungus was confronted with bacteria (Log_2_-fold-change < −2; Supplementary Table 3). The number of proteins encoded by significantly regulated genes, which could be assigned to a GO term was limited, but several candidates could be assigned to one or more significantly enriched categories (Tables 1, 2). The conserved domains of these candidates were further analyzed using the EnsemblFungi webpage (Yates et al., 2020) (Supplementary Tables 4, 5). 11 significantly down-regulated transcripts encode proteins, which are likely involved in the degradation of pectin. They were sorted to the biological process categories ‘cell wall organization’, ‘cell wall organization or biogenesis’, ‘external encapsulating structure organization’ and ‘cellular component organization’. In addition, eight of them were also assigned to the molecular function ‘polygalacturonase activity’. Other identified down-regulated candidates are connected to cellular transport processes. These proteins are represented in the categories ‘SNARE complex disassembly’, ‘cellular component organization’, ‘vesicle-mediated transport’ and ‘structural constituent of cytoskeleton’. For 16 candidates the GO terms ‘tRNA aminoacylation for protein translation’, ‘tRNA aminoacylation’, ‘catalytic activity, acting on RNA’, ‘ligase activity, forming carbon-oxygen bonds’, ‘aminoacyl-tRNA ligase activity’ and ‘catalytic activity, acting on a tRNA’ were identified. Further 11 down-regulated transcripts encode proteins that are predicted to act on RNA (GO term ‘catalytic activity, acting on RNA’). The three candidates from the group ‘N-glycan processing’ are predicted to be involved in protein glycosylation. Additional candidates were grouped into the categories ‘monosaccharide catabolic process’ and ‘monosaccharide metabolic process’. One GO term of the group cellular component with eight proteins was enriched, namely ‘proteasome core complex’.

**TABLE 1:** GO term enrichment of proteins encoded by genes down-regulated after fungal co-cultivation with P_DAPG for 120 min (*p*-value < 0.0001 and Log_2_-fold-change < −2).

GO category	GO term	Adjusted *p*-value	Number of candidates
Biological process	tRNA aminoacylation for protein translation (GO:0006418)	0.005049635	16
Biological process	Monosaccharide catabolic process (GO:0046365)	0.006376761	5
Biological process	Cell wall organization (GO:0071555)	0.009380084	12
Biological process	External encapsulating structure organization (GO:0045229)	0.009380084	12
Biological process	Monosaccharide metabolic process (GO:0005996)	0.013036605	11
Biological process	SNARE complex disassembly (GO:0035494)	0.014166236	4
Biological process	tRNA aminoacylation (GO:0043039)	0.01478278	16
Biological process	Cellular component organization (GO:0016043)	0.020421763	34
Biological process	Cell wall organization or biogenesis (GO:0071554)	0.024867501	14
Biological process	Vesicle-mediated transport (GO:0016192)	0.028088849	28
Biological process	N-glycan processing (GO:0006491)	0.032130268	3
Cellular component	Proteasome core complex (GO:0005839)	0.047398555	8
Molecular function	Structural constituent of cytoskeleton (GO:0005200)	5.69437E-05	7
Molecular function	Catalytic activity, acting on RNA (GO:0140098)	0.000109369	27
Molecular function	Polygalacturonase activity (GO:0004650)	0.002643383	8
Molecular function	Ligase activity, forming carbon-oxygen bonds (GO:0016875)	0.00650204	16
Molecular function	Aminoacyl-tRNA ligase activity (GO:0004812)	0.00650204	16
Molecular function	Catalytic activity, acting on a tRNA (GO:0140101)	0.014137349	19
Molecular function	Phosphoglycerate mutase activity (GO:0004619)	0.049844805	3

**TABLE 2:** GO term enrichment of proteins encoded by genes up-regulated after fungal co-cultivation with P_DAPG for 120 min (*p*-value < 0.0001 and Log_2_-fold-change > 2).

GO category	GO term	Adjusted *p*-value	Number of candidates
Biological process	Cellular aldehyde metabolic process (GO:0006081)	0.002079647	8
Biological process	Aldehyde biosynthetic process (GO:0046184)	0.002655749	6
Biological process	Vitamin B6 biosynthetic process (GO:0042819)	0.002655749	6
Biological process	Vitamin B6 metabolic process (GO:0042816)	0.002655749	6
Biological process	Pyridoxal phosphate metabolic process (GO:0042822)	0.002655749	6
Biological process	Protoporphyrinogen IX metabolic process (GO:0046501)	0.003121445	8
Biological process	Protoporphyrinogen IX biosynthetic process (GO:0006782)	0.003121445	8
Biological process	Heme biosynthetic process (GO:0006783)	0.004950441	11
Biological process	Porphyrin-containing compound biosynthetic process (GO:0006779)	0.011773111	11
Biological process	Porphyrin-containing compound metabolic process (GO:0006778)	0.01751914	11
Biological process	Tetrapyrrole biosynthetic process (GO:0033014)	0.01751914	11
Biological process	Pyridoxal phosphate biosynthetic process (GO:0042823)	0.024094575	6
Biological process	Tetrapyrrole metabolic process (GO:0033013)	0.025526976	11
Biological process	Proline metabolic process (GO:0006560)	0.030412694	8
Molecular function	Oxygen binding (GO:0019825)	0.001584484	6
Molecular function	Oxidoreductase activity (GO:0016491)	0.002147285	176
Molecular function	Proline dehydrogenase activity (GO:0004657)	0.031266391	5
Molecular function	Amine-lyase activity (GO:0016843)	0.036201599	3
Molecular function	Pyridoxal 5’-phosphate synthase (glutamine hydrolyzing) activity (GO:0036381)	0.036201599	3

The largest group of transcripts (176), which were found to be up-regulated in response to bacterial co-cultivation, could be assigned to the molecular function GO term ‘oxidoreductase activity’. This group contains a great variety of different enzymes, which are linked to different cellular processes. One example of this category is a potential manganese/iron superoxide dismutase (BN1708_001526), which might be involved in stress response. Genes encoding proteins, which might be involved in ER redox homeostasis were also identified in this group (BN1708_006230 and BN1708_014150). Five encoded proteins of the oxidoreductases also belong to the group ‘proline dehydrogenase activity’ and are also associated with the biological process ‘proline metabolic process’, to which three additional candidates were also assigned. Six candidates were sorted to the GO term ‘oxygen binding’, including putative protoglobins/globins. Three proteins were categorized to the GO terms ‘amine-lyase activity’ and ‘pyridoxal 5’-phosphate synthase (glutamine hydrolyzing) activity’. Several biological process associated GO terms were significantly enriched in the group of up-regulated transcripts (Table 2). This includes proteins involved in the biosynthesis of aldehydes, vitamin B6 and heme.

These co-cultivation data suggest that the presence of the bacterium induces a *V. longisporum* response of reduced transcription of genes, which encode enzymes for the degradation of pectin, the carbon source in the surrounding medium. Additionally, cellular transport and protein biosynthesis as well as degradation are presumably down-regulated. In parallel, *P. protegens* P_DAPG causes a significantly higher fungal expression of genes with oxidoreductase activities. The bacterial induction of changes in fungal growth, morphology and transcription might be sufficient to protect plants from fungal infection.

### *Pseudomonas* spp. Protect Roots From *V. dahliae* Colonization

*Verticillium* fungi colonize their host plants starting at the roots. This is also the place where they might encounter bacteria, which inhabit the plant rhizosphere, and where mutually antagonistic interactions take place. Root colonization of *V. dahliae* in presence of respective bacteria was monitored to examine whether *Pseudomonas* strains can inhibit *Verticillium* spp. at the entry point of their plant hosts. Roots of the model plant *Arabidopsis thaliana* were used for these experiments. Two days prior to infection, roots of the seedlings were treated with a fresh bacterial suspension. Plants were treated with sterile water as control. *V. dahliae* wild type ectopically expressing high amounts of *GFP* was used for fungal infection. Roots were inspected by fluorescence microscopy five days after infection.

**FIGURE 3:**
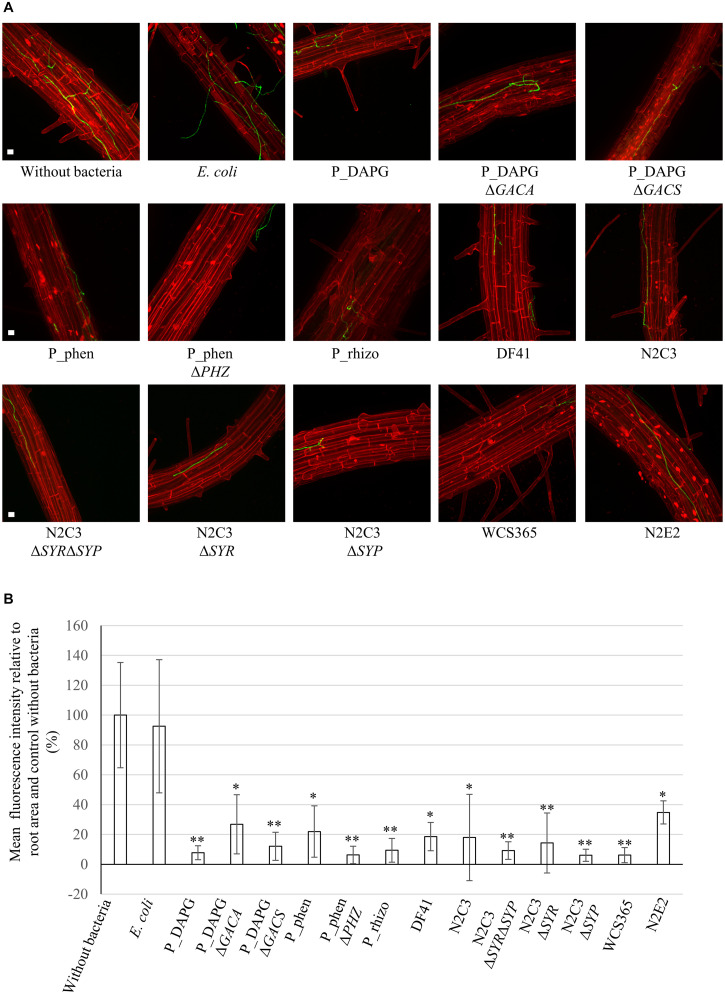
*Verticillium dahliae* avoids efficient colonization of *Arabidopsis thaliana* roots in the presence of *Pseudomona*s strains. *A. thaliana* seedlings were pre-grown on plate and roots were coated with water (without bacteria) or a bacterial suspension of the indicated strains (OD_600__*nm*_ = 0.001). Two days later, plants were infected by root dipping in spore suspension of *V. dahliae* JR2 expressing *GFP*. After five days, fungal colonization of the root surface was investigated by fluorescence microscopy. Roots were stained with propidium iodide. Data are derived from three to four independent experiments. Per experiment, fungal colonization of two roots was analyzed for each individual treatment. For each root 10-15 stacks of single micrographs from randomly chosen root sections were pictured and projections created. **(A)** Single projection pictures generated from stacks of micrographs for each treatment. Roots of the control without bacteria or with *E. coli* were colonized by fungal hyphae. When *Pseudomonas* strains were applied to the roots prior to infection, large parts of the roots remained free of fungus or showed only mild colonization. The pictures show examples of fungal hyphae, which were detected on the root surface. Scale bars: 20 μm. **(B)** Quantification of green fluorescence of fungal hyphae relative to the root area and the respective control without bacteria. The mean value of treatment without bacteria was set as 100%. Depicted is the mean of three to four biological replicates conducted as described above. Error bars indicate the standard deviation between biological replicates. Significant differences to the control without bacteria were calculated with two-tailed Student’s T-test (^∗^*p* < 0.05, ^∗∗^*p* < 0.01). Differences to control are indicated on top of the bars. There was no significant difference between wild type strains and their respective mutants. All *Pseudomonas* isolates are able to reduce fungal colonization of the root surface.

Roots of plants, which were not treated with bacteria but only water, showed a strong colonization with fungal hyphae on the root surface (Figure 3A). Similarly, roots of plants, which were exposed to *E. coli* cells, were successfully colonized by the fungus. In contrast, less fungal hyphae could be detected on roots, which have been treated with a suspension containing cells of different *Pseudomonas* strains. Large parts of the root were free of fungal hyphae, other parts were only slightly colonized (Figure 3A; only pictures with fungal hyphae are shown). Green fluorescence of hyphae was quantified relative to the root surface inspected and normalized to the control without bacteria (Figure 3B). Colonization was decreased to approximately 8% when P_DAPG was applied to the plants before infection. Even the *GACS* or *GACA* deletion could reduce the detected fungus on the root surface significantly (27% and 12% compared to the untreated control), which could be due to Gac-independent compounds.

The bacterial isolates P_phen and P_rhizo also efficiently inhibited fungal growth, as only 22% or 9% of fungal hyphae were detected on the root surface compared to the control. The function of P_phen in root protection was independent of the cluster for phenazine production, as the phenazine deficient mutant showed similar protection capacities (P_phenΔ*PHZ*) as the wild type. The lipopeptide producing strains DF41 and N2C3 were also able to significantly reduce the number of fungal hyphae on the root (19% and 18% of hyphae compared to the control). This effect was independent from the production of syringomycin and/or syringopeptin as respective deletion strains (N2C3Δ*SYP*, N2C3Δ*SYR*, N2C3Δ*SYR*Δ*SYP*) or the non-producing isolate WCS365 also prevented the fungus to efficiently colonize the root surface. The isolate N2E2, which showed a strong inhibitory potential toward *V. dahliae* on plate, was able to significantly inhibit fungal colonization of the root but overall showed the weakest effect of all tested bacteria with 35% of fungal hyphae on the root compared to the control.

Taken together, these data suggest that treatment of roots prior to *Verticillium* infection significantly reduced the number of fungal hyphae on the root surface. This effect was not significantly dependent on the genomic potential to produce phenazines, lipopeptides or several metabolites controlled by the GacS/GacA system. This suggests a general protective effect of different *Pseudomonas* isolates on the root surface against the plant pathogen *V. dahliae.* By reducing the fungal burden on the root, the bacteria might decrease the intensity of Verticillium wilt, accordingly.

## Discussion

**FIGURE 4:**
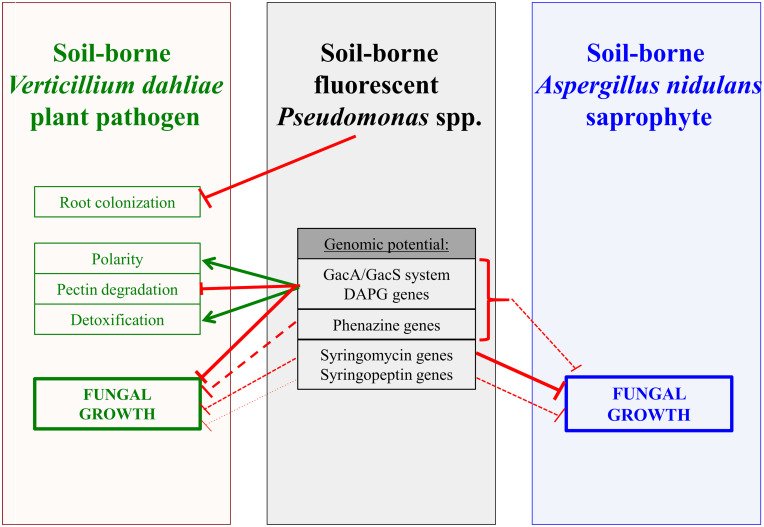
Interplay of *Pseudomona*s strains with fungal phytopathogen *Verticillium dahliae* or saprophyte *Aspergillus nidulans*. Fluorescent pseudomonads isolated from different rhizospheres or groundwater affect *Verticillium dahliae* and its amphidiploid hybrid *V. longisporum* as well as growth of *A. nidulans*. Inhibitory and promoting activities of the bacteria are indicated by red connectors and green arrows, respectively. Dashed lines and smaller line width indicate weaker effects. The bacterial GacS/GacA two component system controls the formation of several bacterial metabolites. For details see text. DAPG: 2,4-diacetylphloroglucinol (DAPG).

Treatment of the devastating Verticillium wilt disease is difficult as the most efficient agent for soil fumigation, methyl bromide is no longer allowed, because it can harm the environment (Carroll et al., 2018). Other fungicides are ineffective once the fungus has entered the plant (Sande et al., 2011; Deketelaere et al., 2017; Huang et al., 2019b). The use of antagonistic organisms is an alternative to protect plants from fungal pathogen infection (Uppal et al., 2008; Angelopoulou et al., 2014; Deketelaere et al., 2017; Mulero-Aparicio et al., 2020). Verticillium wilt symptoms of cotton plants can be attenuated by the addition of *P. protegens* or *P. donghuensis* isolates with an inhibitory effect of the metabolite 7-hydroxytropolone, which can be monitored during co-cultivation on plates (Tao et al., 2020). Here, the impacts of different bacterial *Pseudomonas* spp. with various genetic potentials for secondary metabolite formation were dissected at different interaction levels with amphidiploid *Verticillium longisporum* or haploid *V. dahliae*. This includes plant root colonization, polarity changes at the fungal hyphal tips, pectin substrate degradation, detoxification of bacterial compounds and the fungal growth rate in general. The complex bacterial influence on the fungus, which was analyzed, is summarized in Figure 4.

The root is the fungal entry point into the plant. Treatment of *A. thaliana* roots with a bacterial solution of low amounts of different *Pseudomonas* isolates two days prior to *V. dahliae* spore infection is sufficient to decrease the number of fungal hyphae on the root surface significantly (Figure 3). Competition for space has been suggested as one potential mechanism (Deketelaere et al., 2017). Plants can also manipulate their root microbiome to attract beneficial bacteria that can help to reduce pathogen attack (Pascale et al., 2020). In our experiments, we applied the bacteria prior to fungal infection, but did not examine if simultaneous or later application of bacteria would still allow reduction of pathogen colonization. An important contribution to the decrease in fungal colonization presumably is bacterial inhibition of fungal spore germination, which can even not be re-established after co-cultivation when bacteria were removed (Nesemann et al., 2018). In addition, the fungus might sense and avoid roots where bacteria are present. In nature, colonization of the host usually starts with fungal hyphae instead of conidia, which derive from stress-resistant microsclerotia, which can survive in the soil for several years (Wilhelm, 1955). Fungal hyphal growth also suggests an avoidance behavior toward *Pseudomonas* isolates and might not necessarily depend on the production of specific bacterial metabolites. Confrontation of single fungal hyphae with various *Pseudomonas* strains in microfluidic devices resulted not only in a slowdown of fungal growth, but also in polarity changes at the tip of the hyphae (Figure 2). The fungus is not able to evade the bacterium in these interaction devices filled with liquid medium. The tips of single hyphae start to redirect growth in the channels, leading to a curly phenotype. This suggests that the fungus senses its environment, including the presence of bacteria and/or secretion of bacterial metabolites. Co-cultures of the plant pathogenic fungus *Rhizoctonia solani* with *Serratia* spp. bacteria resulted in a different type of morphology change, with swollen fungal hyphae linked to increased branch and septa formation. The *R. solani* fungal cell wall is modified in co-culture with the bacterium, which is reflected in the transcriptome where genes involved in chitin metabolism as well as genes for ergosterol biosynthesis are down-regulated (Gkarmiri et al., 2015). Growth direction changes of *V. longisporum* in co-culture with *Pseudomonas* spp. might be a strategy to avoid contact with the bacteria and potentially harmful metabolites.

Confrontation of the fungus with the bacterium does not only lead to a slowdown but might even stop fungal growth. Analysis of different mutant strains of the *Pseudomonas* P_DAPG with *Verticillium* in microfluidic devices revealed that the contribution of bacterial genes for single metabolites as DAPG, pyoluteorin or HCN to fungal growth inhibition is significantly lower than the impairment of the bacterial GacS/GacA two-component regulatory system, which controls the formation of a cocktail of metabolites. This is consistent with earlier findings of bacterial-fungal surface co-cultures on plates (Nesemann et al., 2018). *V. longisporum* is able to distinguish between pectin-rich medium, plant xylem sap, and other culture media and adapts its secretion response accordingly (Leonard et al., 2020). The perception of external signals and an adequate fungal response requires intracellular signaling pathways, which have important functions in development and virulence of *Verticillium* spp. (Rauyaree et al., 2005; Qi et al., 2016; Li et al., 2019; Yu et al., 2019; Starke et al., 2021). Transcriptional changes of the fungus caused during bacterial co-cultivation with P_DAPG in liquid medium revealed that transcripts encoding proteins required for the degradation of pectin as main carbon source of the co-culture medium were among the most down-regulated transcripts within 120 min (Supplementary Table 5). This slowdown in metabolism presumably correlates with reduced fungal growth. It is an open question, whether bacteria cause nutrient starvation, and the decreased fungal expression of pectinolytic genes is a result of this depletion. Cellular transport processes as well as transcripts potentially involved in protein synthesis and turnover are also down-regulated (Table 1). Similar responses have been observed for other fungi during growth in unfavorable conditions. When the human opportunistic pathogen *A. fumigatus* is cultured in human blood, it also senses the environment and then down-regulates energy-consuming processes while turning into a kind of resting mycelium (Irmer et al., 2015).

*Botrytis cinerea* induces the expression of ABC transporters in the presence of phenazines or DAPG (Schoonbeek et al., 2002). Some of the *V. longisporum* transporter encoding genes with significantly increased expression (e.g., the potential ABC transporters BN1708_002864 and BN1708_002867, Supplementary Table 2) might be required for the export of toxic bacterial substances from the fungal cell. Significantly induced *V. longisporum* transcripts that encode the carbon-nitrogen hydrolase (BN1708_019975 and BN1708_004875, Supplementary Table 2) potentially protect against bacterial HCN formation as part of the fungal detoxification and stress response. HCN acts on the electron transport chain (Hamel, 2011). This suggests that an increased expression of genes encoding alternative oxidases (BN1708_000782 and BN1708_017690; Supplementary Tables 2, 4) is a possible reaction of the fungus to cope with bacterial metabolites. Similarly, increased expression of the manganese/iron superoxide dismutase might be a direct stress response of the fungus.

Fungal secondary metabolite clusters can be induced in combination with genes for detoxification during fungal-bacterial interactions (Gkarmiri et al., 2015). The presence of *Pseudomonas* P_DAPG hardly affects the expression of *V. longisporum* secondary metabolite genes. BLAST searches for genes encoding several polyketide synthases, non-ribosomal peptide synthases, a hybrid polyketide synthase-non-ribosomal peptide synthase as well as genes for the production of terpenes and other secondary metabolites (Shi-Kunne et al., 2019) revealed that only the genes potentially coding for a non-ribosomal peptide synthase (BN1708_006789 and BN1708_014277) were up-regulated in our RNAseq analysis. Regulation of secondary metabolism and development is regulated by the velvet family of transcription factors. The *V. dahliae* velvet protein Vel1 is required for initial root colonization, transport within the plant by conidiation, control of secondary metabolism as well as resting structure formation for long time survival in the soil (Höfer et al., 2021).

Transcription of three glycoside hydrolase family 24 member genes was induced in *Coprinopsis cinerea* co-cultivated with bacteria and bacterial lysis was confirmed with purified proteins (Kombrink et al., 2019). One hint for antibacterial *Verticillium* activity is the increased transcript level (BN1708_003720, Supplementary Table 2) of the gene encoding a glycoside hydrolase for potential degradation of bacterial cell wall peptidoglycan. The predicted extracellular localization supports that the fungus secretes the enzyme against bacteria in the liquid medium.

The potential of *Pseudomonas* isolates to control growth of different fungi, which they might encounter in soil, varies considerably. *Pseudomonas* strains P_DAPG, P_phen and P_rhizo, isolated from the rhizosphere of different plants, had a greater inhibitory potential toward the plant pathogen *V. dahliae* than toward the saprophyte *A. nidulans* or the opportunistic human pathogen *A. fumigatus* when cultivated on solid medium (Nesemann et al., 2018). In contrast, the inhibitory potential of lipopetide producing *Pseudomonas brassicacearum* DF41, which inhibits the growth of *Sclerotinia sclerotiorum* (Berry et al., 2010, 2012), is significantly higher toward *Aspergillus* strains than to *Verticillium* spp. The overall effect of syringomycin is stronger than of syringopeptin (Figure 1). Lipopeptides act on the plasma membrane of plants and fungi and increase their permeability (Hutchison et al., 1995; Hutchison and Gross, 1997). Nonapeptide lactones including syringomycin have more antimicrobial activity, whereas syringopeptins are rather phytotoxic (Iacobellis et al., 1992; Lavermicocca et al., 1997; Dalla Serra et al., 1999). The addition of cell wall degrading enzymes in the soil, which permeabilize the cell wall, can increase the antifungal activity of syringopeptins, which are rather big molecules (Fogliano et al., 2002). Syringomycin is lethal for *Aspergillus* spp. (De Lucca et al., 1999). Germinating conidia from different fungal species from diseased grapes are killed by a combination of syringomycin and rhamnolipids (Takemoto et al., 2010).

In summary, fluorescent *Pseudomonas* strains with their metabolic diversity have a great and not yet fully explored potential to protect plants from fungal infections, such as Verticillium wilt. It is important to investigate the potential of respective bacteria to protect natural host plants as rapeseed from *V. longisporum* and tomato, lettuce, strawberry or olive trees from *V. dahliae*, respectively. Plant protection happens on multiple levels including control of hyphal growth and root colonization. The interactions need to be further explored to fully understand the mechanism by which inhibition, colonization reduction or ultimately plant protection occur.

## Data Availability Statement

The datasets presented in this study can be found in online repositories. The names of the repository/repositories and accession number(s) can be found below: https://www.ncbi.nlm.nih.gov/, SRP068348.

## Author Contributions

The study was designed and conceived by RH, AN, KN, CS, MK, CH, SB-S, and GB. RH, AN, KN, AH, EB, MStö, and KH performed and analyzed the experiments. Formal analysis was undertaken by all authors. The whole project was supervised by RH, SB-S, GB. RH, AN, KN, SB-S, and GB wrote the manuscript. All authors interpreted the results, revised, and approved the final manuscript.

## Conflict of Interest

The authors declare that the research was conducted in the absence of any commercial or financial relationships that could be construed as a potential conflict of interest.

## Footnotes

1 https://www.ncbi.nlm.nih.gov/sra/?term=SRP068348
