# Supplementary material for: Pseudomonas Strains Induce Transcriptional and Morphological Changes and Reduce Root Colonization of Verticillium spp
Source: Front Microbiol. 2021 May 24;12:652468. doi: 10.3389/fmicb.2021.652468 (PMC8180853; doi:10.3389/fmicb.2021.652468)
Supplement: Supplementary file 1 [file Data_Sheet_1.PDF]

Supplementary Material

1 Supplementary Figures and Tables

1.1 Supplementary Tables

Table S1: Fungal and bacterial strains used.

	**Isolate/ background strain**	**Description**	**Reference**	**Short name**
Verticillium spp.				
V. dahliae	JR2	Wild type	(Fradin et al., 2009)	
V. dahliae	JR2	GFP-expressing strain	(Tran et al., 2014)	
V. longisporum	Vl43	Wild type	(Zeise and von Tiedemann, 2001)	
V. longisporum	Vl43	GFP-expressing strain	(Eynck et al., 2007)	
Aspergillus spp.				
A. nidulans	A4	Wild type	FGSC (Pontecorvo et al., 1953)	
A. fumigatus	AfS35	Wild type with deletion of *akuA*	Derivative of AfS28 (Krappmann et al., 2006)	
Pseudomonas spp.				
P. protegens	CHA0	Wild type	(Stutz et al., 1986)	P_DAPG
P. protegens	CHA0	Deletion of *phlA*	(Schnider-Keel et al., 2000)	P_DAPGΔ*PHLA*
P. protegens	CHA0	Deletion of *phlF*	(Schnider-Keel et al., 2000)	P_DAPGΔ*PHLF*
P. protegens	CHA0	Deletion of *hcnABC*	(Laville et al., 1998)	P_DAPGΔ*HCN*
P. protegens	CHA0	Deletion of *anr*	(Laville et al., 1998)	P_DAPGΔ*ANR*
P. protegens	CHA0	Deletion of *plt*	(Maurhofer et al., 1994)	P_DAPGΔ*PLT*
P. protegens	CHA0	Deletion of *gacA*	(Laville et al., 1992)	P_DAPGΔ*GACA*
P. protegens	CHA0	Deletion of *gacS*	(Zuber et al., 2003)	P_DAPGΔ*GACS*
P. synxantha	2-79	Wild type	(Weller and Cook, 1983)	P_phen

**Table S1: Fungal and bacterial strains used, continued.**				
	**Isolate/ background strain**	**Description**	**Reference**	**Short name**
P. synxantha	2-79	Deletion of parts of phz-D and phz-E by replacement with lacZ	(Khan et al., 2005)	P_phenΔ*PHZ*
P. fluorescens	DSM8569	Wild type	(Berg and Ballin, 1994)	P_rhizo
P. brassica-cearum	DF41	Wild type	(Savchuk and Fernando, 2004)	DF41
Pseudomonas sp.	FW300-N2C3	Wild type	(Price et al., 2018)	N2C3
Pseudomonas sp.	FW300-N2C3	Deletion of lipopeptide transcriptional activator *LUXR*	(Melnyk et al., 2019)	N2C3Δ*LUXR*
Pseudomonas sp.	FW300-N2C3	Deletion of cluster for SYR and SYP synthesis	(Melnyk et al., 2019)	N2C3Δ*SYR*Δ*SYP*
Pseudomonas sp.	FW300-N2C3	Deletion of SYR synthesis genes	(Melnyk et al., 2019)	N2C3Δ*SYR*
Pseudomonas sp.	FW300-N2C3	Deletion of SYP synthesis genes	(Melnyk et al., 2019)	N2C3Δ*SYP*
Pseudomonas sp.	FW300-N2E2	Wild type	(Thorgersen et al., 2015)	N2E2
Pseudomonas sp.	WCS365	Wild type	(Geels and Schippers, 1983)	WCS365
Escherichia coli				
E. coli	DH5α		(Woodcock et al., 1989)	*E. coli*

FGSC: Fungal Genetic Stock Center (Manhattan, Kansas, USA) (McCluskey et al., 2010)

Table S2: List of *Verticillium* genes most up-regulated after 120 min of co-cultivation with *Pseudomonas protegens* P_DAPG. The reads of *V. longisporum* Vl43 have been mapped to the *V. longisporum* VL1 genome. Most up-regulated genes with Log_2-fold-change > 2 and p < 0.0001 have been chosen for further analysis.

Identifier	Log_2-fold-change	P-value	Adjusted p-value
BN1708_000033	3.16	7.65E-98	3.14E-96
BN1708_000037	3.06	7.15E-185	8.91E-183
BN1708_000038	3.89	2.35E-177	2.75E-175
BN1708_000039	4.42	7.95E-193	1.07E-190
BN1708_000040	2.89	8.66E-58	1.84E-56
BN1708_000095	2.38	3.29E-11	1.71E-10
BN1708_000096	2.85	1.23E-22	1.12E-21
BN1708_000097	3.11	5.66E-62	1.29E-60
BN1708_000105	2.46	8.38E-17	5.83E-16
BN1708_000106	2.13	5.00E-42	7.85E-41
BN1708_000107	4.38	0	0
BN1708_000108	7.30	6.75E-145	5.09E-143
BN1708_000112	7.40	1.22E-94	4.78E-93
BN1708_000122	2.03	3.93E-45	6.52E-44
BN1708_000126	2.31	4.78E-82	1.56E-80
BN1708_000135	2.39	7.64E-86	2.61E-84
BN1708_000145	2.03	1.53E-14	9.57E-14
BN1708_000161	3.36	8.33E-08	3.31E-07
BN1708_000168	4.31	1.06E-124	6.08E-123
BN1708_000169	5.26	0	0
BN1708_000170	2.76	1.07E-25	1.09E-24
BN1708_000171	2.37	1.73E-143	1.25E-141
BN1708_000187	2.21	1.14E-08	4.89E-08
BN1708_000199	2.04	2.11E-10	1.03E-09
BN1708_000205	2.19	9.45E-07	3.41E-06
BN1708_000206	2.40	2.53E-110	1.22E-108
BN1708_000207	2.24	7.06E-83	2.32E-81
BN1708_000208	3.20	6.34E-36	8.60E-35
BN1708_000250	2.15	1.49E-35	2.00E-34
BN1708_000251	2.22	4.55E-54	9.05E-53
BN1708_000325	2.03	2.44E-20	2.02E-19
BN1708_000326	2.18	1.69E-15	1.11E-14
BN1708_000328	2.95	6.22E-31	7.36E-30
BN1708_000355	3.19	1.22E-18	9.27E-18
BN1708_000391	2.10	2.88E-99	1.20E-97
BN1708_000395	2.74	3.39E-196	4.93E-194
BN1708_000398	2.09	1.69E-64	4.03E-63
BN1708_000406	2.99	3.01E-50	5.67E-49
BN1708_000452	3.48	1.79E-233	4.44E-231

**Table S2: List of *Verticillium* genes most up-regulated after 120 min of co-cultivation with *Pseudomonas protegens* P_DAPG, continued.**			
**Identifier**	**Log_2-fold-change**	**P-value**	**Adjusted p-value**
BN1708_000489	2.16	3.81E-64	9.02E-63
BN1708_000517	2.73	8.56E-168	8.63E-166
BN1708_000520	3.23	4.62E-26	4.76E-25
BN1708_000521	3.28	3.01E-38	4.32E-37
BN1708_000536	2.33	1.15E-39	1.72E-38
BN1708_000553	3.49	8.69E-73	2.39E-71
BN1708_000554	4.31	2.13E-49	3.94E-48
BN1708_000556	3.84	4.20E-53	8.23E-52
BN1708_000580	2.20	9.52E-126	5.62E-124
BN1708_000584	2.04	4.67E-70	1.22E-68
BN1708_000589	2.27	6.27E-117	3.29E-115
BN1708_000591	2.69	8.11E-136	5.41E-134
BN1708_000605	2.02	3.68E-54	7.34E-53
BN1708_000638	2.23	1.41E-22	1.27E-21
BN1708_000649	2.39	1.35E-12	7.61E-12
BN1708_000651	2.59	4.46E-19	3.46E-18
BN1708_000674	2.26	2.04E-35	2.74E-34
BN1708_000675	2.56	7.02E-79	2.15E-77
BN1708_000678	2.15	1.89E-71	5.09E-70
BN1708_000705	2.03	1.47E-08	6.24E-08
BN1708_000741	2.35	9.78E-109	4.61E-107
BN1708_000743	2.36	2.83E-109	1.35E-107
BN1708_000744	2.26	6.83E-101	2.92E-99
BN1708_000750	2.42	7.52E-44	1.22E-42
BN1708_000782	4.66	0	0
BN1708_000784	2.22	3.71E-57	7.80E-56
BN1708_000824	2.45	2.94E-144	2.17E-142
BN1708_000825	2.68	6.97E-149	5.63E-147
BN1708_000844	2.56	1.24E-93	4.81E-92
BN1708_000862	3.14	7.70E-07	2.80E-06
BN1708_000866	2.45	4.47E-11	2.30E-10
BN1708_000898	2.27	3.86E-18	2.87E-17
BN1708_000899	3.73	1.99E-149	1.61E-147
BN1708_000907	2.06	6.86E-42	1.08E-40
BN1708_000908	2.26	1.04E-29	1.19E-28
BN1708_000910	2.09	1.74E-06	6.10E-06
BN1708_000915	2.01	6.81E-20	5.49E-19
BN1708_000918	2.20	3.40E-38	4.88E-37
BN1708_000921	2.15	8.32E-38	1.18E-36
BN1708_000922	2.20	5.18E-36	7.05E-35

**Table S2: List of *Verticillium* genes most up-regulated after 120 min of co-cultivation with *Pseudomonas protegens* P_DAPG, continued.**			
**Identifier**	**Log2-fold-change**	**P-value**	**Adjusted p-value**
BN1708_000954	2.46	2.82E-92	1.07E-90
BN1708_000956	3.37	1.10E-262	3.76E-260
BN1708_000957	2.04	9.93E-31	1.17E-29
BN1708_000971	2.71	9.60E-143	6.91E-141
BN1708_000972	3.39	1.54E-265	5.35E-263
BN1708_001031	2.86	2.83E-224	5.94E-222
BN1708_001039	3.32	1.85E-269	6.92E-267
BN1708_001040	3.24	6.64E-233	1.58E-230
BN1708_001067	3.56	1.23E-81	3.98E-80
BN1708_001082	2.48	1.85E-162	1.76E-160
BN1708_001083	2.09	2.13E-76	6.17E-75
BN1708_001089	2.40	1.78E-32	2.21E-31
BN1708_001090	3.90	2.33E-146	1.80E-144
BN1708_001097	2.37	1.56E-47	2.76E-46
BN1708_001109	2.44	6.21E-92	2.33E-90
BN1708_001164	2.69	1.31E-05	4.17E-05
BN1708_001198	2.17	4.24E-92	1.60E-90
BN1708_001233	2.02	1.74E-10	8.53E-10
BN1708_001249	3.57	2.06E-31	2.47E-30
BN1708_001256	4.88	1.62E-237	4.12E-235
BN1708_001257	6.28	0	0
BN1708_001261	2.04	1.39E-47	2.46E-46
BN1708_001265	4.36	9.68E-160	8.92E-158
BN1708_001267	5.97	2.10E-240	5.90E-238
BN1708_001272	2.28	9.55E-18	6.96E-17
BN1708_001273	2.85	5.68E-15	3.62E-14
BN1708_001274	2.08	2.43E-18	1.82E-17
BN1708_001318	2.15	2.02E-75	5.83E-74
BN1708_001360	2.20	1.22E-125	7.15E-124
BN1708_001361	2.66	2.04E-183	2.52E-181
BN1708_001386	3.20	6.12E-197	9.04E-195
BN1708_001423	2.52	1.38E-55	2.83E-54
BN1708_001471	2.28	1.18E-54	2.38E-53
BN1708_001472	2.30	4.23E-87	1.47E-85
BN1708_001475	2.53	2.98E-28	3.27E-27
BN1708_001476	2.39	6.71E-83	2.20E-81
BN1708_001478	2.52	1.85E-49	3.42E-48
BN1708_001499	3.53	3.94E-291	2.09E-288
BN1708_001501	3.55	1.12E-266	4.04E-264
BN1708_001526	2.73	1.77E-45	2.99E-44
BN1708_001527	3.12	2.87E-21	2.46E-20

Table S2: List of *Verticillium* genes most up-regulated after 120 min of co-cultivation with *Pseudomonas protegens* P_DAPG, continued.

Identifier	Log_2-fold-change	P-value	Adjusted p-value
BN1708_001548	3.22	4.36E-217	8.58E-215
BN1708_001554	2.13	7.35E-65	1.76E-63
BN1708_001557	2.58	6.04E-35	8.01E-34
BN1708_001563	2.50	3.82E-08	1.57E-07
BN1708_001565	2.89	5.78E-61	1.29E-59
BN1708_001566	4.29	3.13E-280	1.33E-277
BN1708_001572	2.72	5.86E-11	2.99E-10
BN1708_001573	2.70	1.90E-71	5.11E-70
BN1708_001574	3.44	5.74E-175	6.51E-173
BN1708_001578	2.47	5.35E-09	2.35E-08
BN1708_001594	2.51	2.64E-34	3.44E-33
BN1708_001602	2.50	7.16E-24	6.79E-23
BN1708_001612	2.20	1.46E-18	1.11E-17
BN1708_001642	4.52	1.19E-70	3.14E-69
BN1708_001645	2.26	1.90E-78	5.77E-77
BN1708_001671	3.58	5.17E-12	2.82E-11
BN1708_001674	2.28	2.78E-61	6.26E-60
BN1708_001679	2.13	7.37E-77	2.15E-75
BN1708_001686	3.10	3.81E-33	4.82E-32
BN1708_001741	2.09	9.65E-14	5.79E-13
BN1708_001753	3.04	2.93E-94	1.14E-92
BN1708_001755	2.15	2.54E-107	1.17E-105
BN1708_001789	2.31	3.05E-09	1.37E-08
BN1708_001790	3.17	6.40E-169	6.59E-167
BN1708_001796	2.99	3.13E-217	6.21E-215
BN1708_001797	3.33	2.43E-52	4.70E-51
BN1708_001831	2.54	1.25E-28	1.39E-27
BN1708_001849	4.06	3.24E-34	4.22E-33
BN1708_001881	2.19	2.60E-75	7.48E-74
BN1708_001941	2.12	1.31E-31	1.59E-30
BN1708_001955	3.17	6.35E-167	6.27E-165
BN1708_002017	2.14	2.49E-75	7.19E-74
BN1708_002018	2.91	2.14E-186	2.70E-184
BN1708_002026	2.53	3.20E-173	3.57E-171
BN1708_002027	2.62	1.64E-173	1.84E-171
BN1708_002029	2.54	8.91E-120	4.84E-118
BN1708_002035	2.07	2.68E-18	2.00E-17
BN1708_002036	3.76	0	0
BN1708_002045	2.49	2.27E-17	1.63E-16
BN1708_002054	2.70	2.36E-18	1.77E-17

**Table S2: List of *Verticillium* genes most up-regulated after 120 min of co-cultivation with *Pseudomonas protegens* P_DAPG, continued.**			
**Identifier**	**Log_2-fold-change**	**P-value**	**Adjusted p-value**
BN1708_002055	2.24	6.47E-21	5.45E-20
BN1708_002097	2.71	7.53E-07	2.74E-06
BN1708_002145	2.75	1.81E-128	1.10E-126
BN1708_002150	3.46	2.41E-193	3.28E-191
BN1708_002156	2.82	1.82E-199	2.94E-197
BN1708_002172	2.96	4.36E-05	0.000130773
BN1708_002180	3.53	4.94E-163	4.75E-161
BN1708_002181	2.22	2.11E-24	2.04E-23
BN1708_002188	2.62	2.12E-129	1.31E-127
BN1708_002192	3.43	2.78E-167	2.76E-165
BN1708_002194	3.52	3.64E-142	2.60E-140
BN1708_002196	4.77	0	0
BN1708_002197	2.50	2.09E-51	3.99E-50
BN1708_002198	2.44	7.77E-45	1.28E-43
BN1708_002211	3.68	4.35E-21	3.70E-20
BN1708_002249	2.61	4.32E-81	1.38E-79
BN1708_002289	4.35	0	0
BN1708_002293	3.88	0	0
BN1708_002305	3.63	4.87E-211	9.10E-209
BN1708_002307	3.79	9.53E-281	4.23E-278
BN1708_002350	2.49	9.49E-11	4.76E-10
BN1708_002351	3.10	1.89E-11	9.94E-11
BN1708_002357	2.20	2.29E-109	1.10E-107
BN1708_002434	2.99	1.76E-168	1.79E-166
BN1708_002435	2.33	2.56E-17	1.83E-16
BN1708_002437	3.79	1.56E-187	2.02E-185
BN1708_002446	2.41	1.41E-110	6.86E-109
BN1708_002454	2.81	1.57E-197	2.36E-195
BN1708_002455	2.80	7.61E-162	7.21E-160
BN1708_002458	2.47	2.91E-18	2.17E-17
BN1708_002461	2.91	5.41E-144	3.97E-142
BN1708_002488	2.56	1.55E-13	9.16E-13
BN1708_002490	2.02	1.19E-34	1.57E-33
BN1708_002506	2.83	2.76E-12	1.53E-11
BN1708_002510	2.90	2.71E-08	1.13E-07
BN1708_002528	2.02	5.60E-64	1.32E-62
BN1708_002570	2.29	5.33E-25	5.29E-24
BN1708_002576	2.01	3.00E-51	5.69E-50
BN1708_002580	2.36	4.89E-67	1.21E-65
BN1708_002590	3.20	1.18E-61	2.68E-60
BN1708_002591	2.39	2.08E-10	1.02E-09

Table S2: List of *Verticillium* genes most up-regulated after 120 min of co-cultivation with *Pseudomonas protegens* P_DAPG, continued.

Identifier	Log_2-fold-change	P-value	Adjusted p-value
BN1708_002604	2.68	4.92E-159	4.47E-157
BN1708_002606	3.39	1.12E-39	1.68E-38
BN1708_002621	2.89	1.36E-226	2.88E-224
BN1708_002624	2.90	3.70E-233	9.04E-231
BN1708_002637	2.76	1.66E-73	4.60E-72
BN1708_002639	2.58	4.26E-27	4.54E-26
BN1708_002643	4.37	5.12E-31	6.08E-30
BN1708_002652	3.10	6.81E-16	4.53E-15
BN1708_002688	4.22	2.59E-259	8.52E-257
BN1708_002726	2.53	4.85E-11	2.49E-10
BN1708_002751	2.21	1.27E-10	6.32E-10
BN1708_002775	3.28	7.04E-145	5.28E-143
BN1708_002858	3.52	3.24E-05	9.86E-05
BN1708_002864	5.25	2.44E-239	6.65E-237
BN1708_002867	4.97	4.34E-290	2.18E-287
BN1708_002870	2.32	3.95E-17	2.80E-16
BN1708_002882	2.81	8.10E-36	1.10E-34
BN1708_002905	2.39	1.38E-107	6.42E-106
BN1708_003004	3.32	5.64E-304	3.84E-301
BN1708_003005	3.83	0	0
BN1708_003006	3.43	3.14E-31	3.76E-30
BN1708_003040	2.32	7.16E-61	1.60E-59
BN1708_003068	2.69	9.86E-61	2.19E-59
BN1708_003075	2.63	2.21E-64	5.26E-63
BN1708_003092	2.10	2.26E-78	6.85E-77
BN1708_003095	3.24	1.66E-31	2.00E-30
BN1708_003098	2.39	5.43E-17	3.82E-16
BN1708_003099	2.92	3.32E-122	1.87E-120
BN1708_003122	3.53	7.26E-133	4.61E-131
BN1708_003132	2.22	9.46E-07	3.41E-06
BN1708_003155	3.02	2.00E-119	1.08E-117
BN1708_003156	4.44	6.09E-176	6.99E-174
BN1708_003191	3.17	2.20E-156	1.93E-154
BN1708_003202	3.35	1.59E-206	2.76E-204
BN1708_003210	2.49	1.91E-148	1.53E-146
BN1708_003211	2.72	4.78E-49	8.72E-48
BN1708_003217	2.67	5.23E-124	2.98E-122
BN1708_003220	2.13	3.57E-69	9.19E-68
BN1708_003267	2.09	2.85E-64	6.77E-63
BN1708_003272	2.74	4.52E-159	4.12E-157

**Table S2: List of *Verticillium* genes most up-regulated after 120 min of co-cultivation with *Pseudomonas protegens* P_DAPG, continued.**			
**Identifier**	**Log_2-fold-change**	**P-value**	**Adjusted p-value**
BN1708_003287	2.49	1.39E-80	4.40E-79
BN1708_003299	2.55	1.35E-60	3.00E-59
BN1708_003341	2.50	1.74E-160	1.61E-158
BN1708_003358	2.18	7.52E-22	6.64E-21
BN1708_003375	2.04	2.55E-75	7.34E-74
BN1708_003381	2.88	1.75E-157	1.56E-155
BN1708_003384	3.17	6.79E-15	4.31E-14
BN1708_003385	2.50	8.62E-127	5.15E-125
BN1708_003390	2.73	1.61E-128	9.80E-127
BN1708_003391	2.37	1.20E-112	6.00E-111
BN1708_003402	2.05	2.40E-19	1.89E-18
BN1708_003419	2.58	3.75E-83	1.24E-81
BN1708_003473	2.90	7.15E-05	0.000208412
BN1708_003493	2.93	1.14E-173	1.28E-171
BN1708_003567	2.90	1.14E-61	2.59E-60
BN1708_003570	2.27	4.26E-52	8.22E-51
BN1708_003600	2.12	9.25E-87	3.20E-85
BN1708_003614	2.37	6.95E-73	1.92E-71
BN1708_003621	2.19	2.65E-41	4.11E-40
BN1708_003628	3.04	4.33E-194	5.98E-192
BN1708_003629	2.07	8.40E-107	3.87E-105
BN1708_003643	2.10	3.66E-13	2.13E-12
BN1708_003672	2.04	4.45E-88	1.59E-86
BN1708_003688	3.16	2.39E-80	7.53E-79
BN1708_003691	2.34	1.47E-128	8.99E-127
BN1708_003692	2.32	5.82E-141	4.09E-139
BN1708_003708	2.12	6.42E-104	2.85E-102
BN1708_003719	3.90	7.46E-41	1.14E-39
BN1708_003720	5.06	1.70E-204	2.92E-202
BN1708_003721	2.54	1.80E-29	2.03E-28
BN1708_003741	2.48	1.28E-110	6.24E-109
BN1708_003777	2.12	6.96E-101	2.97E-99
BN1708_003780	2.07	6.83E-81	2.17E-79
BN1708_003861	2.12	3.65E-52	7.07E-51
BN1708_003885	3.01	7.49E-105	3.36E-103
BN1708_003890	2.38	4.43E-14	2.71E-13
BN1708_003893	2.34	2.79E-13	1.64E-12
BN1708_003915	2.88	1.16E-118	6.21E-117
BN1708_003927	3.63	8.83E-128	5.31E-126
BN1708_003956	3.15	3.35E-190	4.44E-188
BN1708_003957	2.20	1.28E-76	3.72E-75

Table S2: List of *Verticillium* genes most up-regulated after 120 min of co-cultivation with *Pseudomonas protegens* P_DAPG, continued.

Identifier	Log_2-fold-change	P-value	Adjusted p-value
BN1708_003965	2.84	4.30E-61	9.66E-60
BN1708_003966	3.08	2.33E-86	7.99E-85
BN1708_003967	3.44	4.37E-155	3.78E-153
BN1708_004011	2.46	6.08E-20	4.92E-19
BN1708_004023	2.37	2.15E-128	1.30E-126
BN1708_004028	2.08	8.71E-88	3.10E-86
BN1708_004067	2.02	1.04E-109	4.99E-108
BN1708_004107	3.55	1.03E-133	6.69E-132
BN1708_004108	2.24	7.69E-18	5.64E-17
BN1708_004151	2.66	1.27E-107	5.91E-106
BN1708_004154	4.03	1.55E-144	1.15E-142
BN1708_004197	3.53	9.50E-154	8.01E-152
BN1708_004246	3.72	5.79E-14	3.51E-13
BN1708_004282	2.57	2.13E-09	9.65E-09
BN1708_004283	4.33	0	0
BN1708_004353	2.18	1.25E-18	9.50E-18
BN1708_004405	2.58	2.91E-92	1.11E-90
BN1708_004406	2.66	8.12E-146	6.19E-144
BN1708_004408	2.75	6.34E-147	4.97E-145
BN1708_004425	2.06	1.24E-22	1.12E-21
BN1708_004467	2.33	2.00E-45	3.36E-44
BN1708_004536	2.93	2.05E-13	1.21E-12
BN1708_004539	2.07	8.69E-87	3.01E-85
BN1708_004546	2.68	2.72E-39	4.03E-38
BN1708_004585	2.02	6.99E-17	4.88E-16
BN1708_004601	2.54	2.13E-35	2.86E-34
BN1708_004604	2.48	3.59E-13	2.10E-12
BN1708_004631	2.08	1.47E-13	8.75E-13
BN1708_004636	2.62	1.01E-96	4.06E-95
BN1708_004651	2.76	2.22E-97	9.06E-96
BN1708_004655	2.13	1.17E-87	4.15E-86
BN1708_004656	2.84	4.91E-134	3.19E-132
BN1708_004660	2.94	2.87E-230	6.37E-228
BN1708_004669	2.52	2.22E-133	1.42E-131
BN1708_004724	2.26	6.67E-138	4.58E-136
BN1708_004725	2.90	3.73E-31	4.46E-30
BN1708_004726	5.18	0	0
BN1708_004727	3.80	4.07E-71	1.09E-69
BN1708_004729	2.29	3.92E-97	1.59E-95
BN1708_004766	2.12	1.31E-87	4.62E-86

**Table S2: List of *Verticillium* genes most up-regulated after 120 min of co-cultivation with *Pseudomonas protegens* P_DAPG, continued.**			
**Identifier**	**Log$_2$-fold-change**	**P-value**	**Adjusted p-value**
BN1708_004808	3.64	5.97E-100	2.51E-98
BN1708_004825	2.22	3.58E-106	1.63E-104
BN1708_004826	2.27	6.41E-63	1.49E-61
BN1708_004846	2.29	3.33E-88	1.20E-86
BN1708_004855	2.51	1.02E-33	1.31E-32
BN1708_004867	2.64	8.68E-106	3.95E-104
BN1708_004868	3.84	3.52E-240	9.73E-238
BN1708_004869	3.84	6.53E-239	1.75E-236
BN1708_004874	4.23	5.21E-126	3.09E-124
BN1708_004875	3.92	1.00E-81	3.25E-80
BN1708_004897	2.63	3.23E-64	7.68E-63
BN1708_004921	2.52	8.75E-153	7.29E-151
BN1708_004924	2.37	8.02E-110	3.86E-108
BN1708_004934	3.03	3.66E-208	6.58E-206
BN1708_004945	2.18	3.87E-78	1.16E-76
BN1708_004949	3.68	1.01E-34	1.33E-33
BN1708_004979	2.05	1.07E-128	6.58E-127
BN1708_004983	3.84	5.68E-169	5.89E-167
BN1708_005003	3.50	2.69E-228	5.89E-226
BN1708_005004	2.93	3.41E-103	1.51E-101
BN1708_005005	3.78	3.85E-203	6.44E-201
BN1708_005006	3.07	6.98E-253	2.22E-250
BN1708_005008	2.80	1.19E-171	1.27E-169
BN1708_005009	3.92	3.61E-296	2.02E-293
BN1708_005016	2.85	3.45E-103	1.52E-101
BN1708_005019	3.43	7.81E-82	2.54E-80
BN1708_005042	2.77	1.06E-48	1.91E-47
BN1708_005048	2.01	1.99E-32	2.46E-31
BN1708_005052	2.05	2.53E-33	3.21E-32
BN1708_005053	2.67	6.03E-60	1.32E-58
BN1708_005065	2.27	4.91E-74	1.38E-72
BN1708_005066	2.20	7.29E-65	1.75E-63
BN1708_005069	2.94	8.89E-29	9.91E-28
BN1708_005074	2.80	2.65E-100	1.12E-98
BN1708_005078	3.75	3.94E-09	1.75E-08
BN1708_005104	2.28	7.44E-07	2.71E-06
BN1708_005120	3.04	4.45E-191	5.94E-189
BN1708_005124	2.35	1.91E-122	1.08E-120
BN1708_005144	3.38	7.82E-153	6.54E-151
BN1708_005148	3.20	5.65E-197	8.41E-195
BN1708_005154	3.01	2.27E-101	9.82E-100

Table S2: List of *Verticillium* genes most up-regulated after 120 min of co-cultivation with *Pseudomonas protegens* P_DAPG, continued.

Identifier	Log_2-fold-change	P-value	Adjusted p-value
BN1708_005158	3.43	8.58E-173	9.35E-171
BN1708_005165	3.35	3.63E-68	9.13E-67
BN1708_005183	2.43	7.91E-93	3.03E-91
BN1708_005184	2.24	2.81E-80	8.83E-79
BN1708_005254	2.64	4.64E-119	2.50E-117
BN1708_005255	2.23	1.34E-74	3.80E-73
BN1708_005261	2.59	2.11E-187	2.72E-185
BN1708_005262	2.29	1.31E-83	4.35E-82
BN1708_005264	2.29	1.44E-145	1.09E-143
BN1708_005265	2.37	1.50E-148	1.21E-146
BN1708_005296	3.27	1.64E-30	1.91E-29
BN1708_005304	2.57	4.59E-134	3.00E-132
BN1708_005313	3.14	2.89E-125	1.68E-123
BN1708_005320	2.37	1.30E-17	9.41E-17
BN1708_005327	2.63	6.83E-125	3.94E-123
BN1708_005328	2.66	1.57E-115	8.11E-114
BN1708_005334	2.42	2.74E-39	4.05E-38
BN1708_005444	3.01	4.22E-126	2.51E-124
BN1708_005466	2.03	7.97E-89	2.88E-87
BN1708_005490	2.19	2.09E-25	2.10E-24
BN1708_005500	2.09	4.37E-112	2.16E-110
BN1708_005501	2.07	9.72E-17	6.74E-16
BN1708_005503	2.94	3.50E-74	9.83E-73
BN1708_005549	2.14	2.40E-99	1.00E-97
BN1708_005555	2.46	1.94E-62	4.46E-61
BN1708_005564	2.98	3.35E-132	2.11E-130
BN1708_005565	3.59	6.58E-11	3.34E-10
BN1708_005568	3.32	3.11E-27	3.33E-26
BN1708_005569	2.57	3.66E-19	2.85E-18
BN1708_005570	2.72	1.97E-33	2.52E-32
BN1708_005595	2.13	8.28E-71	2.20E-69
BN1708_005615	2.04	9.47E-17	6.57E-16
BN1708_005616	2.10	2.52E-28	2.77E-27
BN1708_005617	2.68	5.18E-36	7.05E-35
BN1708_005625	2.49	2.36E-87	8.30E-86
BN1708_005680	2.56	6.53E-23	5.98E-22
BN1708_005685	2.31	9.97E-77	2.91E-75
BN1708_005687	3.66	3.94E-87	1.38E-85
BN1708_005737	2.21	4.69E-101	2.01E-99
BN1708_005765	2.18	4.02E-83	1.33E-81

**Table S2: List of *Verticillium* genes most up-regulated after 120 min of co-cultivation with *Pseudomonas protegens* P_DAPG, continued.**			
**Identifier**	**Log$_2$-fold-change**	**P-value**	**Adjusted p-value**
BN1708_005789	2.59	1.73E-08	7.30E-08
BN1708_005791	2.93	6.87E-152	5.65E-150
BN1708_005798	2.94	2.59E-141	1.84E-139
BN1708_005813	2.14	2.94E-106	1.35E-104
BN1708_005817	2.77	1.01E-211	1.90E-209
BN1708_005818	2.73	2.48E-196	3.64E-194
BN1708_005822	2.27	3.45E-87	1.21E-85
BN1708_005832	2.18	1.05E-121	5.86E-120
BN1708_005839	2.05	6.80E-62	1.55E-60
BN1708_005843	2.16	3.50E-24	3.36E-23
BN1708_005846	3.08	3.31E-80	1.03E-78
BN1708_005847	2.80	1.19E-72	3.27E-71
BN1708_005906	2.29	3.09E-12	1.71E-11
BN1708_005935	2.31	1.22E-83	4.04E-82
BN1708_005936	2.42	7.65E-50	1.43E-48
BN1708_005954	2.78	6.23E-199	9.89E-197
BN1708_005956	3.13	6.89E-211	1.28E-208
BN1708_005995	2.94	1.54E-165	1.50E-163
BN1708_005998	3.64	6.30E-250	1.91E-247
BN1708_005999	6.07	0	0
BN1708_006000	6.11	0	0
BN1708_006002	2.31	2.10E-17	1.51E-16
BN1708_006003	2.29	3.72E-15	2.39E-14
BN1708_006006	2.56	3.16E-66	7.72E-65
BN1708_006025	2.28	2.35E-12	1.31E-11
BN1708_006033	2.44	4.72E-57	9.90E-56
BN1708_006098	3.50	0	0
BN1708_006099	3.34	4.26E-13	2.48E-12
BN1708_006129	2.22	1.07E-70	2.84E-69
BN1708_006132	2.04	1.29E-11	6.88E-11
BN1708_006157	3.04	1.12E-98	4.67E-97
BN1708_006158	2.43	4.80E-05	0.000143275
BN1708_006161	3.09	4.20E-62	9.60E-61
BN1708_006174	2.12	9.92E-53	1.94E-51
BN1708_006219	2.81	9.88E-112	4.88E-110
BN1708_006230	2.29	5.50E-146	4.21E-144
BN1708_006243	2.72	1.07E-129	6.65E-128
BN1708_006252	2.63	1.70E-35	2.28E-34
BN1708_006254	2.60	4.07E-181	4.95E-179
BN1708_006286	2.15	1.67E-12	9.38E-12
BN1708_006315	2.62	1.46E-09	6.71E-09

**Table S2: List of *Verticillium* genes most up-regulated after 120 min of co-cultivation with *Pseudomonas protegens* P_DAPG, continued.**			
**Identifier**	**Log_2-fold-change**	**P-value**	**Adjusted p-value**
BN1708_006324	2.53	1.43E-05	4.53E-05
BN1708_006382	2.20	7.49E-70	1.95E-68
BN1708_006414	3.38	1.20E-198	1.86E-196
BN1708_006526	2.66	1.05E-28	1.16E-27
BN1708_006562	2.24	6.67E-20	5.39E-19
BN1708_006568	2.34	3.77E-80	1.17E-78
BN1708_006575	3.17	1.51E-59	3.28E-58
BN1708_006593	3.31	4.13E-147	3.25E-145
BN1708_006607	3.49	2.29E-20	1.89E-19
BN1708_006635	2.34	3.02E-58	6.41E-57
BN1708_006683	2.49	1.26E-125	7.40E-124
BN1708_006703	2.78	5.02E-17	3.53E-16
BN1708_006741	2.94	3.50E-75	1.00E-73
BN1708_006772	2.44	7.24E-135	4.79E-133
BN1708_006773	2.31	1.05E-97	4.29E-96
BN1708_006784	2.12	3.78E-61	8.50E-60
BN1708_006787	3.11	2.28E-51	4.35E-50
BN1708_006788	3.25	8.38E-52	1.61E-50
BN1708_006789	2.11	1.79E-32	2.22E-31
BN1708_006798	2.53	9.32E-39	1.36E-37
BN1708_006832	2.13	9.82E-41	1.50E-39
BN1708_006838	3.63	2.47E-20	2.04E-19
BN1708_006847	2.17	4.20E-33	5.30E-32
BN1708_006859	2.36	2.56E-49	4.70E-48
BN1708_006880	2.49	1.34E-133	8.62E-132
BN1708_006881	2.89	1.26E-10	6.24E-10
BN1708_006885	2.68	6.18E-14	3.74E-13
BN1708_006894	2.95	5.28E-35	7.02E-34
BN1708_006898	2.54	1.15E-118	6.17E-117
BN1708_006904	2.75	5.55E-116	2.89E-114
BN1708_006910	2.08	5.81E-81	1.85E-79
BN1708_006920	3.44	8.97E-233	2.11E-230
BN1708_006941	2.83	9.35E-187	1.20E-184
BN1708_006944	4.08	5.77E-208	1.03E-205
BN1708_006945	3.69	2.87E-178	3.38E-176
BN1708_006957	2.49	3.59E-81	1.15E-79
BN1708_006962	2.15	1.50E-36	2.08E-35
BN1708_006971	3.34	5.24E-12	2.86E-11
BN1708_006974	3.11	2.91E-45	4.86E-44
BN1708_006975	2.04	1.48E-06	5.24E-06

**Table S2: List of *Verticillium* genes most up-regulated after 120 min of co-cultivation with *Pseudomonas protegens* P_DAPG, continued.**			
**Identifier**	**Log_2-fold-change**	**P-value**	**Adjusted p-value**
BN1708_006988	3.09	2.22E-21	1.92E-20
BN1708_007007	3.97	1.16E-34	1.52E-33
BN1708_007021	2.21	6.92E-22	6.11E-21
BN1708_007050	2.39	2.98E-09	1.34E-08
BN1708_007146	2.08	5.68E-63	1.32E-61
BN1708_007147	3.69	1.11E-32	1.39E-31
BN1708_007156	2.26	1.41E-06	4.98E-06
BN1708_007179	2.64	2.42E-05	7.46E-05
BN1708_007208	2.42	9.34E-32	1.13E-30
BN1708_007215	2.49	3.08E-39	4.55E-38
BN1708_007222	4.96	1.02E-14	6.40E-14
BN1708_007228	3.78	1.10E-06	3.96E-06
BN1708_007239	2.87	6.17E-168	6.26E-166
BN1708_007256	2.05	1.45E-06	5.12E-06
BN1708_007258	2.22	8.96E-41	1.37E-39
BN1708_007261	3.84	1.95E-33	2.49E-32
BN1708_007262	6.05	0	0
BN1708_007267	2.48	1.25E-112	6.23E-111
BN1708_007270	2.36	5.15E-98	2.13E-96
BN1708_007271	2.33	6.07E-133	3.87E-131
BN1708_007294	3.19	5.40E-170	5.72E-168
BN1708_007328	2.47	5.26E-49	9.59E-48
BN1708_007333	3.06	2.84E-176	3.28E-174
BN1708_007351	2.09	4.05E-26	4.19E-25
BN1708_007372	3.79	9.72E-24	9.17E-23
BN1708_007378	3.62	6.28E-10	2.96E-09
BN1708_007410	2.39	1.37E-06	4.85E-06
BN1708_007450	2.52	4.93E-25	4.91E-24
BN1708_007456	3.57	1.54E-25	1.56E-24
BN1708_007469	3.99	7.84E-86	2.67E-84
BN1708_007470	3.96	7.10E-19	5.44E-18
BN1708_007471	3.07	6.88E-37	9.61E-36
BN1708_007472	3.46	1.75E-216	3.38E-214
BN1708_007520	2.16	1.59E-85	5.38E-84
BN1708_007541	2.50	6.17E-144	4.51E-142
BN1708_007581	2.37	5.86E-105	2.64E-103
BN1708_007602	3.49	2.42E-96	9.65E-95
BN1708_007613	3.09	2.50E-07	9.47E-07
BN1708_007641	2.32	4.86E-65	1.17E-63
BN1708_007675	2.96	1.79E-207	3.17E-205
BN1708_007690	2.91	2.79E-12	1.55E-11

**Table S2: List of *Verticillium* genes most up-regulated after 120 min of co-cultivation with *Pseudomonas protegens* P_DAPG, continued.**			
**Identifier**	**Log_2-fold-change**	**P-value**	**Adjusted p-value**
BN1708_007691	2.71	1.04E-10	5.20E-10
BN1708_007710	2.30	7.46E-129	4.59E-127
BN1708_007762	3.00	5.84E-19	4.50E-18
BN1708_007771	2.66	1.89E-166	1.86E-164
BN1708_007787	2.45	1.68E-34	2.20E-33
BN1708_007811	2.10	3.02E-110	1.46E-108
BN1708_007812	3.19	3.64E-175	4.16E-173
BN1708_007813	2.99	2.96E-46	5.09E-45
BN1708_007832	2.74	3.85E-79	1.18E-77
BN1708_007850	3.76	8.92E-270	3.40E-267
BN1708_007854	2.15	5.38E-20	4.37E-19
BN1708_007862	2.23	4.23E-71	1.13E-69
BN1708_007885	2.08	2.98E-96	1.18E-94
BN1708_007888	4.16	9.46E-73	2.60E-71
BN1708_007889	6.00	0	0
BN1708_007892	2.67	7.14E-15	4.53E-14
BN1708_007896	2.66	3.79E-30	4.40E-29
BN1708_007900	2.44	8.93E-27	9.42E-26
BN1708_007904	2.18	6.01E-08	2.42E-07
BN1708_007911	3.54	3.02E-18	2.25E-17
BN1708_007912	3.78	5.85E-35	7.76E-34
BN1708_007923	2.27	5.10E-14	3.10E-13
BN1708_007932	3.44	0	0
BN1708_007962	2.49	2.50E-60	5.51E-59
BN1708_007988	2.39	7.72E-99	3.21E-97
BN1708_007989	2.25	1.45E-62	3.35E-61
BN1708_007997	2.28	7.00E-37	9.75E-36
BN1708_008064	2.70	3.13E-19	2.45E-18
BN1708_008065	2.44	2.54E-25	2.55E-24
BN1708_008115	2.74	6.87E-19	5.27E-18
BN1708_008121	2.38	1.13E-104	5.07E-103
BN1708_008125	2.12	3.50E-92	1.33E-90
BN1708_008128	5.34	8.81E-18	6.43E-17
BN1708_008136	3.40	1.39E-299	8.52E-297
BN1708_008163	3.09	1.28E-36	1.78E-35
BN1708_008170	2.09	3.53E-66	8.62E-65
BN1708_008175	2.20	3.53E-66	8.62E-65
BN1708_008231	3.36	1.33E-237	3.44E-235
BN1708_008301	2.22	2.24E-137	1.52E-135
BN1708_008304	2.29	2.47E-138	1.70E-136

**Table S2: List of *Verticillium* genes most up-regulated after 120 min of co-cultivation with *Pseudomonas protegens* P_DAPG, continued.**			
**Identifier**	**Log_2-fold-change**	**P-value**	**Adjusted p-value**
BN1708_008306	2.05	5.91E-88	2.11E-86
BN1708_008354	4.02	2.63E-54	5.27E-53
BN1708_008355	2.12	3.87E-18	2.87E-17
BN1708_008384	3.20	4.13E-15	2.65E-14
BN1708_008434	2.71	3.23E-05	9.83E-05
BN1708_008552	2.63	3.82E-31	4.56E-30
BN1708_008582	3.01	2.26E-10	1.10E-09
BN1708_008618	2.38	4.91E-68	1.23E-66
BN1708_008619	2.67	2.17E-88	7.79E-87
BN1708_008621	3.09	9.78E-07	3.52E-06
BN1708_008631	2.50	6.93E-09	3.02E-08
BN1708_008638	2.36	6.41E-144	4.66E-142
BN1708_008639	3.44	9.85E-126	5.79E-124
BN1708_008644	2.79	4.97E-81	1.58E-79
BN1708_008671	2.83	2.87E-139	2.00E-137
BN1708_008673	2.31	4.99E-78	1.49E-76
BN1708_008697	2.25	2.13E-89	7.77E-88
BN1708_008730	2.18	1.67E-45	2.83E-44
BN1708_008766	3.16	6.95E-64	1.63E-62
BN1708_008774	2.77	2.76E-33	3.51E-32
BN1708_008778	2.30	6.72E-72	1.83E-70
BN1708_008779	2.34	8.14E-79	2.49E-77
BN1708_008781	4.91	0	0
BN1708_008797	3.69	4.92E-14	3.00E-13
BN1708_008841	3.39	1.91E-148	1.53E-146
BN1708_008844	3.35	1.72E-92	6.58E-91
BN1708_008850	2.65	1.17E-158	1.06E-156
BN1708_008853	3.59	3.57E-29	4.02E-28
BN1708_008858	4.15	0	0
BN1708_008860	4.08	1.44E-272	5.59E-270
BN1708_008877	3.46	3.35E-08	1.38E-07
BN1708_008878	3.61	4.89E-97	1.98E-95
BN1708_008892	2.69	6.20E-23	5.68E-22
BN1708_008894	2.34	1.94E-08	8.15E-08
BN1708_008926	4.89	1.26E-58	2.70E-57
BN1708_008931	2.72	8.18E-87	2.84E-85
BN1708_008932	2.52	1.68E-33	2.14E-32
BN1708_008933	2.56	4.88E-21	4.14E-20
BN1708_008934	2.13	1.13E-20	9.45E-20
BN1708_008936	4.01	0	0
BN1708_008937	2.45	1.19E-37	1.69E-36

Table S2: List of *Verticillium* genes most up-regulated after 120 min of co-cultivation with *Pseudomonas protegens* P_DAPG, continued.

Identifier	Log_2-fold-change	P-value	Adjusted p-value
BN1708_008954	2.15	5.20E-41	7.99E-40
BN1708_008969	2.81	9.20E-74	2.56E-72
BN1708_008982	5.04	0	0
BN1708_008993	2.11	1.17E-10	5.83E-10
BN1708_009012	6.73	5.95E-44	9.70E-43
BN1708_009055	3.34	1.83E-22	1.65E-21
BN1708_009057	2.41	4.85E-64	1.15E-62
BN1708_009111	2.37	1.26E-63	2.95E-62
BN1708_009122	2.09	6.49E-54	1.29E-52
BN1708_009123	2.56	5.78E-12	3.14E-11
BN1708_009128	2.87	2.56E-11	1.34E-10
BN1708_009131	2.30	9.18E-35	1.21E-33
BN1708_009151	2.25	2.09E-81	6.73E-80
BN1708_009172	2.34	6.12E-75	1.74E-73
BN1708_009175	2.08	1.26E-78	3.84E-77
BN1708_009176	2.80	3.44E-106	1.58E-104
BN1708_009178	2.71	1.89E-137	1.29E-135
BN1708_009182	3.00	1.26E-249	3.74E-247
BN1708_009189	2.85	6.80E-231	1.56E-228
BN1708_009208	2.44	3.23E-16	2.18E-15
BN1708_009238	2.26	3.23E-06	1.10E-05
BN1708_009247	3.82	2.18E-11	1.14E-10
BN1708_009248	3.38	7.29E-224	1.51E-221
BN1708_009250	2.12	1.87E-10	9.15E-10
BN1708_009255	2.20	2.36E-08	9.87E-08
BN1708_009273	2.22	5.36E-53	1.05E-51
BN1708_009281	2.11	3.29E-06	1.12E-05
BN1708_009294	2.71	8.79E-84	2.92E-82
BN1708_009295	2.79	5.35E-05	0.000158425
BN1708_009322	2.72	1.42E-140	9.94E-139
BN1708_009365	2.42	5.58E-90	2.04E-88
BN1708_009376	3.13	7.81E-07	2.84E-06
BN1708_009400	2.20	1.48E-28	1.63E-27
BN1708_009421	2.18	4.97E-89	1.81E-87
BN1708_009422	2.70	4.22E-08	1.72E-07
BN1708_009458	2.25	3.20E-17	2.28E-16
BN1708_009470	4.00	1.01E-61	2.30E-60
BN1708_009492	2.50	1.26E-12	7.12E-12
BN1708_009520	2.39	6.41E-19	4.93E-18
BN1708_009548	2.10	3.24E-68	8.18E-67

**Table S2: List of *Verticillium* genes most up-regulated after 120 min of co-cultivation with *Pseudomonas protegens* P_DAPG, continued.**			
**Identifier**	**Log_2-fold-change**	**P-value**	**Adjusted p-value**
BN1708_009561	2.86	1.09E-195	1.57E-193
BN1708_009571	2.28	3.96E-105	1.79E-103
BN1708_009686	2.15	1.96E-59	4.24E-58
BN1708_009688	2.07	5.94E-20	4.81E-19
BN1708_009692	3.78	6.43E-38	9.15E-37
BN1708_009704	2.47	1.69E-154	1.45E-152
BN1708_009705	2.73	2.72E-35	3.63E-34
BN1708_009713	2.18	4.47E-15	2.86E-14
BN1708_009735	3.99	8.29E-15	5.24E-14
BN1708_009747	2.94	7.79E-30	8.94E-29
BN1708_009766	2.69	2.41E-127	1.44E-125
BN1708_009775	2.88	1.43E-154	1.23E-152
BN1708_009804	2.04	8.09E-24	7.66E-23
BN1708_009824	4.34	3.52E-06	1.19E-05
BN1708_009847	3.05	1.84E-36	2.54E-35
BN1708_009848	2.94	9.87E-125	5.67E-123
BN1708_009850	2.05	2.90E-06	9.91E-06
BN1708_009852	2.37	5.79E-81	1.84E-79
BN1708_009888	2.61	1.76E-20	1.46E-19
BN1708_009889	3.29	4.38E-117	2.31E-115
BN1708_009894	2.71	4.15E-10	1.98E-09
BN1708_009905	2.45	4.36E-06	1.46E-05
BN1708_009909	2.11	4.42E-60	9.69E-59
BN1708_009923	2.93	3.70E-148	2.94E-146
BN1708_009931	2.50	3.19E-32	3.92E-31
BN1708_009939	2.15	1.38E-39	2.06E-38
BN1708_009941	2.36	4.24E-07	1.57E-06
BN1708_010006	3.58	8.61E-70	2.24E-68
BN1708_010029	4.46	4.97E-20	4.04E-19
BN1708_010041	2.02	6.41E-88	2.29E-86
BN1708_010044	2.24	1.64E-11	8.67E-11
BN1708_010054	3.87	0	0
BN1708_010062	3.35	1.25E-68	3.18E-67
BN1708_010070	3.46	2.05E-93	7.92E-92
BN1708_010172	2.06	3.60E-16	2.43E-15
BN1708_010222	3.82	1.80E-49	3.34E-48
BN1708_010226	2.83	3.28E-108	1.55E-106
BN1708_010232	3.38	1.91E-54	3.84E-53
BN1708_010233	3.52	1.02E-158	9.25E-157
BN1708_010242	2.42	1.36E-37	1.92E-36
BN1708_010249	2.38	5.33E-36	7.24E-35

Table S2: List of *Verticillium* genes most up-regulated after 120 min of co-cultivation with *Pseudomonas protegens* P_DAPG, continued.

Identifier	Log_2-fold-change	P-value	Adjusted p-value
BN1708_010292	2.90	9.20E-58	1.95E-56
BN1708_010293	2.26	2.06E-28	2.27E-27
BN1708_010309	2.45	9.46E-66	2.30E-64
BN1708_010310	2.95	4.29E-105	1.93E-103
BN1708_010311	2.51	1.69E-07	6.53E-07
BN1708_010312	3.64	1.52E-193	2.08E-191
BN1708_010313	2.42	7.05E-08	2.82E-07
BN1708_010314	3.47	9.21E-209	1.69E-206
BN1708_010315	3.62	4.62E-141	3.26E-139
BN1708_010331	2.07	2.12E-08	8.89E-08
BN1708_010348	2.62	7.50E-72	2.04E-70
BN1708_010349	2.24	1.16E-06	4.15E-06
BN1708_010395	2.65	1.38E-08	5.86E-08
BN1708_010397	3.80	3.75E-08	1.54E-07
BN1708_010519	2.94	1.04E-238	2.76E-236
BN1708_010534	2.34	2.36E-96	9.44E-95
BN1708_010539	2.83	1.05E-188	1.38E-186
BN1708_010547	3.11	3.49E-114	1.78E-112
BN1708_010549	2.23	1.91E-68	4.83E-67
BN1708_010558	2.83	2.48E-14	1.53E-13
BN1708_010559	4.21	3.45E-144	2.54E-142
BN1708_010562	2.35	3.14E-25	3.14E-24
BN1708_010563	4.08	3.79E-78	1.14E-76
BN1708_010582	2.66	4.00E-207	6.99E-205
BN1708_010590	2.72	6.54E-05	0.000191531
BN1708_010601	3.26	1.19E-17	8.65E-17
BN1708_010604	5.81	1.14E-09	5.28E-09
BN1708_010605	2.37	2.97E-11	1.55E-10
BN1708_010606	2.73	1.81E-71	4.91E-70
BN1708_010607	3.03	3.47E-15	2.23E-14
BN1708_010632	2.27	3.18E-38	4.56E-37
BN1708_010633	2.91	5.83E-61	1.30E-59
BN1708_010655	3.12	3.62E-104	1.61E-102
BN1708_010680	2.89	2.70E-77	8.00E-76
BN1708_010695	2.11	8.34E-34	1.07E-32
BN1708_010704	2.65	2.65E-19	2.09E-18
BN1708_010705	2.13	5.97E-114	3.03E-112
BN1708_010710	2.29	2.69E-112	1.34E-110
BN1708_010718	2.81	1.87E-195	2.67E-193
BN1708_010719	2.85	4.79E-113	2.40E-111

**Table S2: List of *Verticillium* genes most up-regulated after 120 min of co-cultivation with *Pseudomonas protegens* P_DAPG, continued.**			
**Identifier**	**Log_2-fold-change**	**P-value**	**Adjusted p-value**
BN1708_010720	2.75	1.17E-201	1.93E-199
BN1708_010733	2.08	9.11E-89	3.29E-87
BN1708_010734	2.52	1.16E-62	2.68E-61
BN1708_010767	4.11	3.66E-95	1.44E-93
BN1708_010768	2.30	5.66E-07	2.08E-06
BN1708_010825	3.64	2.77E-293	1.51E-290
BN1708_010867	2.21	2.68E-08	1.11E-07
BN1708_010893	2.05	2.71E-51	5.14E-50
BN1708_010895	2.58	2.83E-134	1.86E-132
BN1708_010900	2.25	4.35E-21	3.70E-20
BN1708_010901	2.10	1.08E-05	3.48E-05
BN1708_010906	2.43	2.51E-102	1.10E-100
BN1708_010907	2.13	4.33E-35	5.78E-34
BN1708_010923	2.43	1.98E-144	1.47E-142
BN1708_010924	2.26	1.97E-46	3.41E-45
BN1708_010932	2.16	2.20E-57	4.65E-56
BN1708_010962	2.27	5.01E-37	7.02E-36
BN1708_010963	3.47	1.43E-54	2.88E-53
BN1708_010981	2.68	1.42E-81	4.60E-80
BN1708_010982	2.95	1.94E-169	2.03E-167
BN1708_011003	2.91	1.61E-52	3.13E-51
BN1708_011005	2.29	9.29E-39	1.36E-37
BN1708_011006	2.20	3.74E-72	1.02E-70
BN1708_011020	3.76	1.13E-216	2.21E-214
BN1708_011024	2.26	1.96E-151	1.60E-149
BN1708_011038	2.13	1.47E-63	3.43E-62
BN1708_011078	2.24	5.75E-57	1.20E-55
BN1708_011123	2.89	3.51E-70	9.22E-69
BN1708_011133	3.02	2.83E-198	4.31E-196
BN1708_011136	2.39	8.18E-131	5.11E-129
BN1708_011151	2.70	2.42E-152	2.00E-150
BN1708_011152	2.67	1.64E-111	8.09E-110
BN1708_011153	2.74	2.66E-36	3.66E-35
BN1708_011157	3.65	0	0
BN1708_011178	2.05	5.83E-21	4.92E-20
BN1708_011180	2.05	2.66E-78	8.02E-77
BN1708_011183	3.62	5.09E-285	2.43E-282
BN1708_011185	3.89	1.85E-280	8.01E-278
BN1708_011189	2.37	2.26E-14	1.40E-13
BN1708_011193	3.08	6.43E-134	4.17E-132
BN1708_011194	3.49	1.96E-21	1.70E-20

Table S2: List of *Verticillium* genes most up-regulated after 120 min of co-cultivation with *Pseudomonas protegens* P_DAPG, continued.

Identifier	Log_2-fold-change	P-value	Adjusted p-value
BN1708_011203	3.26	4.77E-77	1.40E-75
BN1708_011204	3.42	1.60E-23	1.49E-22
BN1708_011205	4.24	1.36E-57	2.87E-56
BN1708_011206	2.06	6.35E-37	8.88E-36
BN1708_011207	2.03	6.94E-55	1.41E-53
BN1708_011241	2.04	1.91E-56	3.97E-55
BN1708_011247	3.00	2.25E-114	1.15E-112
BN1708_011248	2.67	8.72E-97	3.51E-95
BN1708_011250	2.91	1.23E-96	4.94E-95
BN1708_011264	3.48	1.47E-186	1.87E-184
BN1708_011265	4.26	3.76E-56	7.78E-55
BN1708_011303	2.11	6.68E-121	3.67E-119
BN1708_011306	2.02	3.55E-80	1.11E-78
BN1708_011307	2.10	2.44E-110	1.18E-108
BN1708_011377	2.63	2.63E-10	1.28E-09
BN1708_011387	4.23	8.09E-269	2.96E-266
BN1708_011405	3.00	2.49E-113	1.26E-111
BN1708_011413	2.02	1.59E-06	5.60E-06
BN1708_011438	2.16	1.60E-14	1.00E-13
BN1708_011468	2.56	6.99E-60	1.53E-58
BN1708_011483	3.05	1.08E-113	5.44E-112
BN1708_011486	3.01	5.03E-19	3.90E-18
BN1708_011517	2.50	1.22E-47	2.16E-46
BN1708_011531	2.07	1.57E-105	7.13E-104
BN1708_011572	3.23	2.18E-22	1.96E-21
BN1708_011582	2.52	9.78E-121	5.36E-119
BN1708_011583	2.36	9.76E-81	3.09E-79
BN1708_011605	2.96	3.30E-195	4.66E-193
BN1708_011620	3.89	4.09E-196	5.90E-194
BN1708_011621	2.35	8.06E-05	0.000233153
BN1708_011627	2.35	4.50E-81	1.44E-79
BN1708_011629	2.24	4.35E-41	6.71E-40
BN1708_011642	2.38	4.18E-09	1.85E-08
BN1708_011685	5.52	3.14E-301	1.99E-298
BN1708_011704	2.43	5.16E-22	4.60E-21
BN1708_011738	2.79	2.76E-15	1.78E-14
BN1708_011752	3.47	4.71E-14	2.88E-13
BN1708_011763	2.32	3.33E-77	9.83E-76
BN1708_011777	2.92	1.14E-29	1.30E-28
BN1708_011789	2.33	7.08E-20	5.70E-19

**Table S2: List of *Verticillium* genes most up-regulated after 120 min of co-cultivation with *Pseudomonas protegens* P_DAPG, continued.**			
**Identifier**	**Log$_2$-fold-change**	**P-value**	**Adjusted p-value**
BN1708_011796	2.01	6.91E-22	6.10E-21
BN1708_011814	4.76	2.27E-302	1.50E-299
BN1708_011815	3.84	2.78E-130	1.73E-128
BN1708_011821	2.45	9.91E-116	5.15E-114
BN1708_011826	2.48	1.11E-50	2.09E-49
BN1708_011827	2.58	1.39E-102	6.09E-101
BN1708_011828	2.64	1.72E-60	3.79E-59
BN1708_011829	2.72	5.91E-193	7.99E-191
BN1708_011849	3.29	2.12E-147	1.68E-145
BN1708_011855	2.65	6.67E-57	1.39E-55
BN1708_011857	2.32	3.55E-30	4.13E-29
BN1708_011878	2.18	1.19E-41	1.86E-40
BN1708_011882	2.52	1.87E-122	1.06E-120
BN1708_011883	2.18	1.92E-67	4.81E-66
BN1708_011885	2.38	2.39E-145	1.81E-143
BN1708_011898	3.63	4.39E-101	1.89E-99
BN1708_011901	2.06	3.35E-41	5.19E-40
BN1708_011913	2.39	3.48E-63	8.12E-62
BN1708_011921	3.15	3.89E-100	1.64E-98
BN1708_011922	2.77	2.83E-28	3.11E-27
BN1708_011923	3.20	3.04E-19	2.39E-18
BN1708_011926	2.84	8.05E-68	2.01E-66
BN1708_011927	2.32	9.69E-19	7.40E-18
BN1708_011928	3.34	2.95E-24	2.85E-23
BN1708_011930	2.32	4.84E-80	1.50E-78
BN1708_011931	2.17	4.35E-43	6.94E-42
BN1708_011950	2.08	4.20E-43	6.71E-42
BN1708_011967	2.37	3.23E-69	8.32E-68
BN1708_011980	2.51	5.24E-05	0.000155402
BN1708_011981	5.24	3.72E-173	4.13E-171
BN1708_011985	3.67	5.25E-34	6.79E-33
BN1708_011993	2.23	6.85E-35	9.06E-34
BN1708_012000	2.12	8.30E-06	2.71E-05
BN1708_012007	2.02	6.05E-09	2.64E-08
BN1708_012013	3.91	1.77E-49	3.28E-48
BN1708_012028	2.02	6.49E-64	1.53E-62
BN1708_012057	2.38	1.10E-17	8.01E-17
BN1708_012063	3.12	1.77E-240	5.03E-238
BN1708_012066	2.38	6.06E-76	1.75E-74
BN1708_012074	2.43	1.82E-07	6.99E-07
BN1708_012079	2.04	4.35E-08	1.77E-07

Table S2: List of *Verticillium* genes most up-regulated after 120 min of co-cultivation with *Pseudomonas protegens* P_DAPG, continued.

Identifier	Log_2-fold-change	P-value	Adjusted p-value
BN1708_012147	2.05	5.94E-11	3.02E-10
BN1708_012160	2.10	1.89E-85	6.39E-84
BN1708_012161	3.05	2.27E-10	1.11E-09
BN1708_012184	2.96	2.28E-22	2.06E-21
BN1708_012185	4.11	5.56E-125	3.22E-123
BN1708_012187	2.68	1.86E-18	1.40E-17
BN1708_012195	2.61	9.34E-125	5.38E-123
BN1708_012222	2.79	5.03E-14	3.07E-13
BN1708_012223	2.52	1.07E-61	2.43E-60
BN1708_012228	2.35	3.02E-29	3.40E-28
BN1708_012230	2.13	2.33E-84	7.80E-83
BN1708_012249	2.46	1.08E-72	2.95E-71
BN1708_012257	2.50	8.49E-59	1.82E-57
BN1708_012265	2.34	6.66E-92	2.49E-90
BN1708_012270	2.49	2.43E-111	1.19E-109
BN1708_012273	3.75	1.28E-298	7.64E-296
BN1708_012274	2.54	6.12E-93	2.35E-91
BN1708_012275	2.38	2.41E-65	5.83E-64
BN1708_012286	3.01	4.51E-36	6.17E-35
BN1708_012314	2.37	1.54E-33	1.96E-32
BN1708_012339	2.13	7.47E-32	9.09E-31
BN1708_012341	2.19	3.85E-57	8.09E-56
BN1708_012365	2.01	1.56E-20	1.30E-19
BN1708_012388	3.36	1.25E-15	8.23E-15
BN1708_012389	2.82	4.68E-156	4.09E-154
BN1708_012392	2.13	6.24E-74	1.75E-72
BN1708_012395	2.66	9.86E-199	1.54E-196
BN1708_012398	2.37	2.27E-143	1.64E-141
BN1708_012443	2.55	4.10E-67	1.02E-65
BN1708_012470	2.38	7.11E-14	4.29E-13
BN1708_012475	2.43	6.55E-91	2.42E-89
BN1708_012530	2.58	3.94E-180	4.76E-178
BN1708_012559	2.41	1.86E-31	2.24E-30
BN1708_012560	2.49	1.12E-13	6.68E-13
BN1708_012567	2.67	9.16E-142	6.52E-140
BN1708_012608	2.07	2.79E-122	1.57E-120
BN1708_012652	2.77	3.16E-84	1.05E-82
BN1708_012660	2.43	1.09E-23	1.03E-22
BN1708_012669	2.68	8.19E-97	3.31E-95
BN1708_012697	2.21	1.15E-19	9.22E-19

**Table S2: List of *Verticillium* genes most up-regulated after 120 min of co-cultivation with *Pseudomonas protegens* P_DAPG, continued.**			
**Identifier**	**Log_2-fold-change**	**P-value**	**Adjusted p-value**
BN1708_012721	3.21	3.28E-17	2.33E-16
BN1708_012726	3.12	1.63E-198	2.50E-196
BN1708_012734	2.03	8.54E-32	1.04E-30
BN1708_012736	2.30	1.03E-07	4.03E-07
BN1708_012739	2.19	7.15E-43	1.13E-41
BN1708_012748	2.23	6.37E-98	2.62E-96
BN1708_012754	2.03	6.37E-30	7.32E-29
BN1708_012763	2.20	3.47E-115	1.79E-113
BN1708_012765	2.23	9.85E-118	5.23E-116
BN1708_012775	2.10	4.13E-69	1.06E-67
BN1708_012776	2.09	5.86E-103	2.58E-101
BN1708_012785	4.81	5.08E-275	2.06E-272
BN1708_012794	2.93	1.04E-181	1.27E-179
BN1708_012808	2.08	8.21E-56	1.69E-54
BN1708_012839	2.08	1.63E-24	1.59E-23
BN1708_012854	3.38	3.32E-109	1.58E-107
BN1708_012855	3.27	1.34E-169	1.42E-167
BN1708_012865	2.13	3.29E-26	3.41E-25
BN1708_012877	2.06	2.05E-78	6.21E-77
BN1708_012884	3.47	2.30E-45	3.85E-44
BN1708_012936	2.33	1.33E-68	3.38E-67
BN1708_012957	2.62	2.12E-15	1.38E-14
BN1708_012971	4.33	6.72E-19	5.16E-18
BN1708_012986	2.19	1.68E-25	1.70E-24
BN1708_013038	2.33	5.69E-56	1.17E-54
BN1708_013039	2.62	9.12E-30	1.04E-28
BN1708_013041	2.17	4.21E-78	1.26E-76
BN1708_013049	2.09	6.30E-39	9.24E-38
BN1708_013050	2.58	7.49E-12	4.04E-11
BN1708_013061	2.76	1.41E-18	1.07E-17
BN1708_013080	2.50	5.58E-60	1.22E-58
BN1708_013118	2.05	3.78E-09	1.68E-08
BN1708_013139	2.14	6.74E-10	3.17E-09
BN1708_013175	2.20	2.28E-112	1.13E-110
BN1708_013191	2.10	2.33E-91	8.65E-90
BN1708_013192	2.16	1.27E-89	4.64E-88
BN1708_013211	3.49	1.63E-138	1.13E-136
BN1708_013227	2.32	8.82E-59	1.89E-57
BN1708_013237	3.03	1.32E-107	6.16E-106
BN1708_013261	2.92	1.07E-22	9.71E-22
BN1708_013273	3.19	2.44E-49	4.49E-48

Table S2: List of *Verticillium* genes most up-regulated after 120 min of co-cultivation with *Pseudomonas protegens* P_DAPG, continued.

Identifier	Log_2-fold-change	P-value	Adjusted p-value
BN1708_013289	2.45	1.82E-98	7.51E-97
BN1708_013296	3.86	2.05E-230	4.59E-228
BN1708_013303	2.19	2.43E-46	4.20E-45
BN1708_013312	2.70	1.59E-08	6.76E-08
BN1708_013343	2.36	1.34E-55	2.74E-54
BN1708_013368	2.26	8.88E-57	1.85E-55
BN1708_013378	4.17	1.58E-103	7.03E-102
BN1708_013407	3.02	6.65E-92	2.49E-90
BN1708_013409	3.54	5.38E-20	4.37E-19
BN1708_013421	2.62	2.06E-176	2.39E-174
BN1708_013441	2.47	3.06E-95	1.20E-93
BN1708_013442	2.18	5.54E-56	1.14E-54
BN1708_013444	2.02	3.42E-75	9.81E-74
BN1708_013562	3.16	1.87E-15	1.22E-14
BN1708_013570	2.22	1.97E-64	4.70E-63
BN1708_013575	2.70	4.62E-75	1.32E-73
BN1708_013576	3.57	2.67E-46	4.61E-45
BN1708_013591	2.54	1.42E-158	1.28E-156
BN1708_013597	2.03	1.87E-36	2.58E-35
BN1708_013615	2.82	3.29E-05	0.000100142
BN1708_013641	2.23	9.52E-73	2.62E-71
BN1708_013654	2.21	2.98E-41	4.62E-40
BN1708_013679	2.07	5.63E-96	2.23E-94
BN1708_013700	3.43	8.46E-198	1.28E-195
BN1708_013701	3.28	4.90E-200	7.98E-198
BN1708_013712	2.09	5.49E-30	6.32E-29
BN1708_013713	2.09	1.09E-23	1.03E-22
BN1708_013727	2.31	6.17E-47	1.08E-45
BN1708_013790	2.27	1.82E-39	2.71E-38
BN1708_013792	3.04	7.52E-227	1.61E-224
BN1708_013797	2.06	1.34E-23	1.26E-22
BN1708_013836	3.18	1.80E-76	5.23E-75
BN1708_013855	2.10	2.13E-27	2.30E-26
BN1708_013877	2.47	1.09E-133	7.02E-132
BN1708_013878	2.31	3.18E-26	3.30E-25
BN1708_013879	4.53	1.68E-54	3.39E-53
BN1708_013880	3.14	1.06E-97	4.32E-96
BN1708_013881	4.42	3.05E-252	9.53E-250
BN1708_013882	2.68	3.98E-52	7.69E-51
BN1708_013883	3.52	1.13E-230	2.57E-228

**Table S2: List of *Verticillium* genes most up-regulated after 120 min of co-cultivation with *Pseudomonas protegens* P_DAPG, continued.**			
**Identifier**	**Log_2-fold-change**	**P-value**	**Adjusted p-value**
BN1708_013884	2.81	1.39E-107	6.43E-106
BN1708_013888	2.50	2.91E-128	1.75E-126
BN1708_013895	2.38	5.62E-20	4.55E-19
BN1708_013897	3.89	8.46E-188	1.10E-185
BN1708_013898	2.84	4.19E-15	2.69E-14
BN1708_013903	3.12	1.98E-199	3.17E-197
BN1708_013904	3.08	5.91E-65	1.42E-63
BN1708_013921	2.64	2.17E-21	1.88E-20
BN1708_013938	2.48	6.57E-23	6.01E-22
BN1708_013954	2.78	4.56E-36	6.23E-35
BN1708_013955	2.26	2.70E-05	8.27E-05
BN1708_014014	2.01	2.76E-57	5.83E-56
BN1708_014029	5.42	9.23E-291	4.75E-288
BN1708_014053	2.05	2.09E-05	6.51E-05
BN1708_014054	2.41	1.15E-21	1.01E-20
BN1708_014060	2.44	7.28E-07	2.65E-06
BN1708_014078	3.39	2.10E-251	6.46E-249
BN1708_014079	2.31	3.51E-122	1.97E-120
BN1708_014105	2.37	5.31E-111	2.60E-109
BN1708_014130	2.54	2.97E-171	3.17E-169
BN1708_014137	2.14	3.80E-58	8.08E-57
BN1708_014150	2.23	2.37E-126	1.41E-124
BN1708_014158	3.38	8.64E-222	1.75E-219
BN1708_014167	3.79	6.40E-201	1.05E-198
BN1708_014208	3.14	2.25E-16	1.53E-15
BN1708_014216	3.69	6.51E-10	3.06E-09
BN1708_014217	3.29	5.71E-12	3.10E-11
BN1708_014222	4.26	1.47E-258	4.76E-256
BN1708_014229	2.94	2.98E-47	5.23E-46
BN1708_014236	2.76	9.26E-135	6.11E-133
BN1708_014242	2.39	1.18E-08	5.04E-08
BN1708_014243	2.91	4.91E-23	4.51E-22
BN1708_014249	3.51	4.92E-228	1.07E-225
BN1708_014250	2.87	3.19E-80	9.99E-79
BN1708_014251	2.81	4.66E-97	1.89E-95
BN1708_014271	2.24	1.64E-65	3.98E-64
BN1708_014275	3.22	1.26E-50	2.39E-49
BN1708_014276	3.34	3.05E-73	8.43E-72
BN1708_014277	2.21	9.21E-42	1.44E-40
BN1708_014329	2.72	1.03E-152	8.57E-151
BN1708_014331	2.88	1.36E-121	7.52E-120

Table S2: List of *Verticillium* genes most up-regulated after 120 min of co-cultivation with *Pseudomonas protegens* P_DAPG, continued.

Identifier	Log_2-fold-change	P-value	Adjusted p-value
BN1708_014337	2.94	1.02E-05	3.30E-05
BN1708_014347	2.45	3.01E-46	5.16E-45
BN1708_014376	2.55	3.32E-24	3.19E-23
BN1708_014380	4.21	0	0
BN1708_014421	2.23	3.36E-40	5.11E-39
BN1708_014422	3.66	6.00E-204	1.01E-201
BN1708_014463	2.23	2.95E-142	2.12E-140
BN1708_014468	3.61	3.54E-154	3.01E-152
BN1708_014478	2.10	7.70E-108	3.61E-106
BN1708_014481	2.89	1.72E-63	4.02E-62
BN1708_014497	2.10	5.13E-69	1.32E-67
BN1708_014511	2.97	1.32E-231	3.07E-229
BN1708_014514	2.78	6.28E-186	7.87E-184
BN1708_014518	2.86	3.22E-33	4.08E-32
BN1708_014521	3.86	0	0
BN1708_014522	2.63	1.30E-150	1.06E-148
BN1708_014586	2.37	5.89E-14	3.57E-13
BN1708_014600	2.81	1.80E-102	7.88E-101
BN1708_014603	2.68	4.19E-136	2.80E-134
BN1708_014617	3.56	1.30E-120	7.11E-119
BN1708_014618	5.98	4.34E-16	2.92E-15
BN1708_014619	2.75	2.47E-06	8.49E-06
BN1708_014632	2.01	3.07E-18	2.29E-17
BN1708_014659	2.50	1.96E-120	1.07E-118
BN1708_014723	2.12	4.18E-122	2.33E-120
BN1708_014724	2.12	3.47E-118	1.85E-116
BN1708_014728	2.42	2.43E-56	5.02E-55
BN1708_014732	2.62	1.65E-53	3.25E-52
BN1708_014761	2.30	7.03E-109	3.33E-107
BN1708_014775	2.37	2.39E-49	4.39E-48
BN1708_014793	2.40	3.70E-93	1.42E-91
BN1708_014794	2.47	1.50E-132	9.49E-131
BN1708_014798	3.32	1.29E-06	4.57E-06
BN1708_014799	4.32	2.53E-219	5.09E-217
BN1708_014831	3.35	2.08E-17	1.50E-16
BN1708_014840	2.04	2.12E-13	1.25E-12
BN1708_014880	3.80	5.67E-273	2.25E-270
BN1708_014888	3.57	3.90E-05	0.000117735
BN1708_014921	2.42	6.33E-66	1.54E-64
BN1708_014936	4.00	1.64E-283	7.46E-281

**Table S2: List of *Verticillium* genes most up-regulated after 120 min of co-cultivation with *Pseudomonas protegens* P_DAPG, continued.**			
**Identifier**	**Log_2-fold-change**	**P-value**	**Adjusted p-value**
BN1708_014937	3.29	3.01E-09	1.35E-08
BN1708_014974	3.16	1.12E-20	9.36E-20
BN1708_014975	2.55	3.90E-70	1.02E-68
BN1708_015012	3.63	6.19E-56	1.27E-54
BN1708_015021	2.65	6.13E-118	3.27E-116
BN1708_015022	2.67	2.07E-154	1.77E-152
BN1708_015025	2.28	1.62E-101	7.02E-100
BN1708_015030	2.46	4.87E-124	2.78E-122
BN1708_015040	2.72	4.31E-129	2.66E-127
BN1708_015064	2.04	3.17E-09	1.42E-08
BN1708_015076	3.06	9.07E-147	7.09E-145
BN1708_015083	2.96	4.16E-195	5.83E-193
BN1708_015108	2.58	2.02E-56	4.20E-55
BN1708_015110	2.21	2.04E-45	3.43E-44
BN1708_015114	2.56	3.95E-115	2.03E-113
BN1708_015115	2.24	2.58E-17	1.85E-16
BN1708_015139	2.13	1.87E-48	3.36E-47
BN1708_015154	2.62	1.56E-167	1.57E-165
BN1708_015175	2.11	3.54E-41	5.47E-40
BN1708_015180	3.00	3.04E-41	4.71E-40
BN1708_015200	3.87	3.48E-106	1.59E-104
BN1708_015225	3.18	1.98E-284	9.21E-282
BN1708_015226	2.93	1.11E-86	3.81E-85
BN1708_015233	3.13	3.27E-121	1.81E-119
BN1708_015234	2.43	3.55E-18	2.64E-17
BN1708_015263	2.74	2.88E-33	3.66E-32
BN1708_015269	2.50	3.03E-79	9.30E-78
BN1708_015288	2.54	1.53E-39	2.27E-38
BN1708_015304	2.55	4.29E-108	2.01E-106
BN1708_015335	3.25	1.67E-35	2.24E-34
BN1708_015336	2.40	6.90E-31	8.15E-30
BN1708_015362	3.02	6.95E-14	4.20E-13
BN1708_015363	2.38	2.42E-134	1.59E-132
BN1708_015372	2.67	4.70E-16	3.16E-15
BN1708_015379	2.92	2.58E-21	2.22E-20
BN1708_015415	2.14	1.50E-37	2.12E-36
BN1708_015447	2.10	3.63E-71	9.74E-70
BN1708_015463	2.31	1.07E-77	3.17E-76
BN1708_015499	2.21	1.15E-27	1.25E-26
BN1708_015500	2.11	1.01E-29	1.15E-28
BN1708_015502	2.15	1.57E-10	7.73E-10

Table S2: List of *Verticillium* genes most up-regulated after 120 min of co-cultivation with *Pseudomonas protegens* P_DAPG, continued.

Identifier	Log_2-fold-change	P-value	Adjusted p-value
BN1708_015503	4.60	9.35E-199	1.47E-196
BN1708_015504	6.01	0	0
BN1708_015527	3.83	3.81E-06	1.29E-05
BN1708_015574	2.04	6.43E-05	0.000188425
BN1708_015614	2.71	2.45E-48	4.38E-47
BN1708_015616	2.04	1.67E-41	2.60E-40
BN1708_015617	2.45	1.04E-09	4.82E-09
BN1708_015626	3.00	1.46E-29	1.65E-28
BN1708_015627	2.61	3.02E-109	1.44E-107
BN1708_015628	2.79	5.93E-111	2.90E-109
BN1708_015636	2.09	1.08E-09	5.01E-09
BN1708_015674	3.23	2.54E-279	1.05E-276
BN1708_015675	3.41	8.57E-06	2.79E-05
BN1708_015680	3.15	1.20E-53	2.37E-52
BN1708_015681	2.14	1.31E-38	1.91E-37
BN1708_015693	2.73	1.18E-171	1.27E-169
BN1708_015704	2.58	9.84E-147	7.66E-145
BN1708_015743	2.91	1.88E-215	3.59E-213
BN1708_015769	2.20	1.75E-84	5.88E-83
BN1708_015796	2.25	6.78E-20	5.47E-19
BN1708_015815	2.13	6.65E-24	6.31E-23
BN1708_015820	2.14	5.21E-35	6.93E-34
BN1708_015827	3.73	2.73E-67	6.79E-66
BN1708_015828	2.06	1.06E-22	9.60E-22
BN1708_015841	2.35	3.93E-19	3.06E-18
BN1708_015846	3.88	2.23E-136	1.49E-134
BN1708_015847	2.37	9.76E-24	9.20E-23
BN1708_015848	2.01	5.93E-14	3.59E-13
BN1708_015849	2.34	3.71E-22	3.32E-21
BN1708_015875	2.95	2.00E-13	1.18E-12
BN1708_015909	2.06	3.00E-77	8.86E-76
BN1708_015910	2.78	6.18E-89	2.24E-87
BN1708_015921	2.71	2.25E-61	5.08E-60
BN1708_015922	2.82	5.14E-92	1.94E-90
BN1708_015964	3.50	1.25E-06	4.44E-06
BN1708_015970	3.97	3.55E-113	1.79E-111
BN1708_015971	3.98	2.76E-266	9.73E-264
BN1708_015978	2.57	5.31E-14	3.23E-13
BN1708_015998	2.43	3.53E-09	1.57E-08
BN1708_016011	2.02	5.22E-13	3.02E-12

**Table S2: List of *Verticillium* genes most up-regulated after 120 min of co-cultivation with *Pseudomonas protegens* P_DAPG, continued.**			
**Identifier**	**Log_2-fold-change**	**P-value**	**Adjusted p-value**
BN1708_016017	3.42	6.45E-244	1.86E-241
BN1708_016018	2.15	4.39E-96	1.74E-94
BN1708_016036	3.64	3.70E-236	9.28E-234
BN1708_016056	2.84	3.35E-57	7.05E-56
BN1708_016064	2.10	4.89E-52	9.43E-51
BN1708_016072	3.59	3.47E-32	4.27E-31
BN1708_016082	3.85	6.14E-21	5.18E-20
BN1708_016083	3.13	3.03E-22	2.71E-21
BN1708_016092	4.06	1.34E-32	1.66E-31
BN1708_016093	4.58	1.07E-35	1.44E-34
BN1708_016168	2.16	1.69E-16	1.16E-15
BN1708_016169	2.11	1.80E-26	1.88E-25
BN1708_016185	2.29	3.72E-43	5.97E-42
BN1708_016206	5.20	1.35E-38	1.95E-37
BN1708_016215	2.32	3.23E-100	1.36E-98
BN1708_016238	2.62	7.45E-107	3.44E-105
BN1708_016254	2.23	6.06E-85	2.04E-83
BN1708_016271	2.18	3.15E-57	6.63E-56
BN1708_016272	2.42	3.07E-10	1.48E-09
BN1708_016279	2.35	9.26E-43	1.47E-41
BN1708_016327	2.14	3.56E-12	1.96E-11
BN1708_016345	2.58	7.36E-10	3.45E-09
BN1708_016347	2.43	3.05E-55	6.22E-54
BN1708_016352	2.23	1.01E-23	9.49E-23
BN1708_016353	2.42	4.18E-12	2.30E-11
BN1708_016393	2.76	1.57E-26	1.64E-25
BN1708_016395	2.24	9.39E-75	2.66E-73
BN1708_016412	3.04	9.29E-13	5.29E-12
BN1708_016439	2.38	3.74E-87	1.31E-85
BN1708_016440	2.02	1.66E-41	2.58E-40
BN1708_016447	4.65	1.92E-247	5.63E-245
BN1708_016448	5.02	1.23E-238	3.22E-236
BN1708_016458	2.14	5.07E-78	1.52E-76
BN1708_016464	2.90	2.63E-85	8.88E-84
BN1708_016485	4.03	7.74E-101	3.29E-99
BN1708_016521	2.93	3.63E-132	2.28E-130
BN1708_016549	2.68	3.23E-95	1.27E-93
BN1708_016560	2.39	5.68E-59	1.22E-57
BN1708_016586	2.01	6.42E-78	1.92E-76
BN1708_016623	2.75	1.38E-10	6.86E-10
BN1708_016625	2.50	2.58E-182	3.18E-180

**Table S2: List of *Verticillium* genes most up-regulated after 120 min of co-cultivation with *Pseudomonas protegens* P_DAPG, continued.**			
**Identifier**	**Log_2-fold-change**	**P-value**	**Adjusted p-value**
BN1708_016631	2.40	2.88E-65	6.96E-64
BN1708_016632	3.39	4.23E-161	3.97E-159
BN1708_016707	2.25	4.40E-91	1.63E-89
BN1708_016738	2.06	9.26E-05	0.000265837
BN1708_016774	3.79	6.48E-63	1.50E-61
BN1708_016797	3.35	2.33E-07	8.86E-07
BN1708_016800	2.88	1.19E-116	6.21E-115
BN1708_016803	2.35	1.68E-15	1.10E-14
BN1708_016815	2.05	4.78E-11	2.46E-10
BN1708_016862	3.42	4.09E-179	4.88E-177
BN1708_016871	2.76	2.30E-47	4.06E-46
BN1708_016887	3.65	6.72E-91	2.48E-89
BN1708_016899	2.25	1.69E-60	3.72E-59
BN1708_016949	3.04	2.52E-159	2.31E-157
BN1708_016982	2.41	1.01E-08	4.34E-08
BN1708_016992	2.06	3.34E-47	5.85E-46
BN1708_017010	2.22	1.51E-22	1.36E-21
BN1708_017021	2.78	3.38E-20	2.77E-19
BN1708_017028	2.01	4.23E-84	1.41E-82
BN1708_017033	2.16	6.05E-23	5.54E-22
BN1708_017070	2.23	3.45E-77	1.02E-75
BN1708_017116	2.26	2.70E-19	2.13E-18
BN1708_017149	2.09	1.11E-10	5.51E-10
BN1708_017158	2.52	5.43E-32	6.64E-31
BN1708_017172	2.27	4.69E-18	3.47E-17
BN1708_017185	3.45	6.31E-139	4.38E-137
BN1708_017187	3.35	1.99E-172	2.15E-170
BN1708_017214	3.35	1.13E-119	6.14E-118
BN1708_017218	2.68	7.50E-12	4.05E-11
BN1708_017220	2.21	1.70E-08	7.18E-08
BN1708_017230	2.50	4.15E-109	1.97E-107
BN1708_017233	3.07	5.71E-122	3.18E-120
BN1708_017240	2.91	7.05E-31	8.32E-30
BN1708_017241	2.01	3.75E-40	5.69E-39
BN1708_017243	2.19	1.61E-10	7.95E-10
BN1708_017246	2.85	4.85E-07	1.79E-06
BN1708_017247	2.03	6.84E-16	4.55E-15
BN1708_017260	2.86	1.25E-168	1.28E-166
BN1708_017298	3.19	4.89E-28	5.34E-27
BN1708_017306	2.61	5.52E-06	1.84E-05

**Table S2: List of *Verticillium* genes most up-regulated after 120 min of co-cultivation with *Pseudomonas protegens* P_DAPG, continued.**			
**Identifier**	**Log_2-fold-change**	**P-value**	**Adjusted p-value**
BN1708_017313	2.38	9.38E-08	3.70E-07
BN1708_017327	2.76	1.64E-208	2.98E-206
BN1708_017339	2.38	1.64E-163	1.60E-161
BN1708_017384	2.02	1.20E-29	1.37E-28
BN1708_017401	3.00	4.00E-22	3.57E-21
BN1708_017437	2.62	3.25E-42	5.12E-41
BN1708_017442	2.37	2.69E-06	9.21E-06
BN1708_017470	2.20	2.34E-12	1.31E-11
BN1708_017472	2.73	2.45E-67	6.11E-66
BN1708_017477	2.05	7.68E-72	2.09E-70
BN1708_017487	2.01	3.21E-49	5.87E-48
BN1708_017496	2.82	6.79E-163	6.50E-161
BN1708_017508	2.07	3.52E-42	5.55E-41
BN1708_017510	2.09	3.57E-11	1.85E-10
BN1708_017551	2.05	5.87E-47	1.03E-45
BN1708_017570	3.10	2.59E-89	9.43E-88
BN1708_017579	2.73	7.79E-08	3.10E-07
BN1708_017602	2.51	7.20E-145	5.38E-143
BN1708_017629	2.60	2.74E-13	1.61E-12
BN1708_017655	3.01	3.19E-155	2.77E-153
BN1708_017682	2.28	4.33E-23	3.99E-22
BN1708_017690	5.07	0	0
BN1708_017707	3.54	2.95E-153	2.48E-151
BN1708_017712	2.32	3.83E-09	1.70E-08
BN1708_017715	2.38	9.36E-136	6.22E-134
BN1708_017722	3.41	1.69E-10	8.33E-10
BN1708_017724	3.17	1.96E-79	6.02E-78
BN1708_017731	2.11	3.34E-62	7.65E-61
BN1708_017740	2.11	1.95E-07	7.49E-07
BN1708_017753	2.97	7.41E-179	8.78E-177
BN1708_017766	2.45	1.77E-07	6.83E-07
BN1708_017768	3.41	1.35E-09	6.21E-09
BN1708_017824	2.22	1.86E-09	8.47E-09
BN1708_017867	2.37	4.65E-19	3.60E-18
BN1708_017874	2.42	9.26E-21	7.76E-20
BN1708_017902	3.14	1.24E-11	6.58E-11
BN1708_017941	2.19	5.60E-16	3.74E-15
BN1708_017955	2.19	4.61E-05	0.000137737
BN1708_017964	2.28	4.77E-05	0.000142207
BN1708_017967	2.35	1.57E-24	1.54E-23
BN1708_017981	2.07	1.55E-20	1.29E-19

Table S2: List of *Verticillium* genes most up-regulated after 120 min of co-cultivation with *Pseudomonas protegens* P_DAPG, continued.

Identifier	Log_2-fold-change	P-value	Adjusted p-value
BN1708_017982	2.37	3.12E-09	1.40E-08
BN1708_018010	2.69	4.02E-15	2.58E-14
BN1708_018019	2.12	7.90E-08	3.14E-07
BN1708_018025	2.05	3.95E-06	1.33E-05
BN1708_018044	4.39	6.68E-288	3.27E-285
BN1708_018049	2.16	2.11E-14	1.31E-13
BN1708_018062	2.93	1.29E-24	1.26E-23
BN1708_018067	3.82	1.71E-05	5.39E-05
BN1708_018098	2.20	9.09E-09	3.92E-08
BN1708_018101	2.05	2.46E-48	4.40E-47
BN1708_018124	2.79	7.84E-06	2.56E-05
BN1708_018134	2.43	3.07E-29	3.46E-28
BN1708_018137	2.42	6.81E-05	0.000198957
BN1708_018148	2.49	9.68E-05	0.00027694
BN1708_018156	2.07	6.06E-57	1.27E-55
BN1708_018171	2.74	4.89E-169	5.09E-167
BN1708_018240	2.94	9.35E-80	2.89E-78
BN1708_018251	2.98	4.32E-121	2.38E-119
BN1708_018342	3.28	4.42E-34	5.73E-33
BN1708_018354	2.79	3.09E-114	1.58E-112
BN1708_018377	2.01	3.56E-19	2.78E-18
BN1708_018391	3.55	1.44E-78	4.39E-77
BN1708_018396	2.44	5.13E-125	2.98E-123
BN1708_018445	2.53	1.13E-61	2.56E-60
BN1708_018459	2.08	9.60E-31	1.13E-29
BN1708_018460	2.11	1.86E-09	8.46E-09
BN1708_018462	2.79	1.82E-77	5.41E-76
BN1708_018486	2.08	3.23E-07	1.21E-06
BN1708_018530	3.99	4.33E-56	8.94E-55
BN1708_018532	3.41	4.30E-42	6.75E-41
BN1708_018541	3.37	4.50E-88	1.61E-86
BN1708_018554	2.41	3.72E-90	1.37E-88
BN1708_018577	3.68	8.85E-195	1.23E-192
BN1708_018581	2.56	3.95E-71	1.06E-69
BN1708_018606	2.79	1.36E-89	4.96E-88
BN1708_018619	2.11	2.48E-16	1.69E-15
BN1708_018661	2.89	8.52E-82	2.77E-80
BN1708_018669	2.20	1.25E-07	4.89E-07
BN1708_018677	2.34	2.22E-51	4.23E-50
BN1708_018679	2.07	6.92E-08	2.77E-07

**Table S2: List of *Verticillium* genes most up-regulated after 120 min of co-cultivation with *Pseudomonas protegens* P_DAPG, continued.**			
**Identifier**	**Log_2-fold-change**	**P-value**	**Adjusted p-value**
BN1708_018685	2.88	2.12E-167	2.12E-165
BN1708_018687	2.39	1.08E-20	9.02E-20
BN1708_018704	2.52	3.02E-82	9.89E-81
BN1708_018720	2.56	1.41E-11	7.49E-11
BN1708_018729	2.14	1.82E-53	3.59E-52
BN1708_018745	2.62	3.99E-157	3.54E-155
BN1708_018753	2.08	2.19E-43	3.53E-42
BN1708_018818	2.97	9.08E-15	5.73E-14
BN1708_018824	3.86	9.98E-63	2.31E-61
BN1708_018871	2.07	7.51E-10	3.51E-09
BN1708_018908	2.19	5.21E-05	0.000154581
BN1708_018914	2.28	1.90E-48	3.41E-47
BN1708_018951	2.29	2.43E-84	8.13E-83
BN1708_018957	2.83	3.00E-26	3.12E-25
BN1708_018961	3.54	1.02E-69	2.65E-68
BN1708_018976	2.62	6.47E-77	1.90E-75
BN1708_019028	2.72	2.06E-105	9.31E-104
BN1708_019039	2.57	1.49E-23	1.39E-22
BN1708_019050	2.16	1.94E-38	2.80E-37
BN1708_019068	2.63	2.32E-10	1.13E-09
BN1708_019091	4.16	3.90E-26	4.03E-25
BN1708_019132	2.45	2.08E-47	3.67E-46
BN1708_019164	2.15	5.16E-11	2.64E-10
BN1708_019179	3.49	1.06E-86	3.67E-85
BN1708_019186	2.21	6.69E-22	5.91E-21
BN1708_019220	2.26	9.98E-18	7.27E-17
BN1708_019235	2.73	7.02E-157	6.20E-155
BN1708_019255	2.41	8.64E-87	3.00E-85
BN1708_019258	2.87	5.99E-09	2.62E-08
BN1708_019260	2.41	2.63E-11	1.37E-10
BN1708_019261	2.10	8.87E-05	0.000255066
BN1708_019290	2.64	8.66E-27	9.15E-26
BN1708_019301	2.42	1.93E-21	1.67E-20
BN1708_019323	2.81	6.30E-173	6.94E-171
BN1708_019382	2.01	7.83E-30	8.97E-29
BN1708_019387	3.34	1.21E-21	1.06E-20
BN1708_019407	2.20	8.74E-06	2.85E-05
BN1708_019418	2.64	9.67E-34	1.24E-32
BN1708_019440	2.52	4.96E-24	4.72E-23
BN1708_019447	2.20	1.30E-22	1.18E-21
BN1708_019483	2.01	1.62E-104	7.25E-103

**Table S2: List of *Verticillium* genes most up-regulated after 120 min of co-cultivation with *Pseudomonas protegens* P_DAPG, continued.**			
**Identifier**	**Log_2-fold-change**	**P-value**	**Adjusted p-value**
BN1708_019526	2.23	1.60E-07	6.21E-07
BN1708_019538	2.04	1.60E-31	1.93E-30
BN1708_019548	3.10	6.20E-13	3.57E-12
BN1708_019565	2.93	3.91E-14	2.39E-13
BN1708_019602	2.27	5.03E-26	5.18E-25
BN1708_019641	4.47	1.69E-20	1.40E-19
BN1708_019664	2.59	2.89E-15	1.87E-14
BN1708_019699	3.62	2.62E-14	1.61E-13
BN1708_019710	4.23	4.98E-38	7.11E-37
BN1708_019717	2.51	1.61E-35	2.16E-34
BN1708_019771	2.51	2.21E-05	6.84E-05
BN1708_019778	3.21	2.48E-115	1.28E-113
BN1708_019795	2.21	5.64E-65	1.36E-63
BN1708_019796	2.12	1.15E-06	4.11E-06
BN1708_019805	3.38	6.48E-09	2.83E-08
BN1708_019808	2.06	1.53E-08	6.51E-08
BN1708_019811	2.58	2.79E-18	2.08E-17
BN1708_019875	2.41	2.93E-46	5.05E-45
BN1708_019877	3.25	1.54E-64	3.68E-63
BN1708_019906	2.18	1.76E-47	3.11E-46
BN1708_019923	2.77	1.39E-49	2.59E-48
BN1708_019948	2.38	1.10E-08	4.73E-08
BN1708_019952	2.35	2.04E-06	7.10E-06
BN1708_019968	3.04	5.25E-05	0.000155831
BN1708_019975	5.79	5.83E-71	1.55E-69
BN1708_019979	2.02	1.08E-19	8.66E-19
BN1708_019989	2.67	1.88E-26	1.97E-25
BN1708_020037	2.17	1.85E-09	8.43E-09
BN1708_020069	2.79	3.02E-08	1.25E-07
BN1708_020078	2.63	1.24E-07	4.83E-07
BN1708_020098	2.59	1.75E-05	5.49E-05
BN1708_020103	2.75	1.59E-19	1.26E-18
BN1708_020106	2.07	1.69E-16	1.16E-15
BN1708_020116	2.36	2.20E-08	9.20E-08
BN1708_020118	2.01	1.31E-12	7.42E-12
BN1708_020152	2.09	1.92E-09	8.73E-09
BN1708_020163	2.84	4.73E-09	2.09E-08
BN1708_020167	3.36	7.86E-11	3.97E-10
BN1708_020170	2.26	1.59E-12	8.96E-12
BN1708_020189	3.07	5.25E-10	2.49E-09

**Table S2: List of *Verticillium* genes most up-regulated after 120 min of co-cultivation with *Pseudomonas protegens* P_DAPG, continued.**			
**Identifier**	**Log_2-fold-change**	**P-value**	**Adjusted p-value**
BN1708_020288	2.67	5.12E-45	8.48E-44
BN1708_020294	3.57	1.52E-54	3.08E-53
BN1708_020310	2.06	1.71E-12	9.61E-12
BN1708_020347	2.06	3.59E-25	3.58E-24
BN1708_020352	2.67	1.29E-09	5.96E-09
BN1708_020353	2.32	1.87E-39	2.77E-38
BN1708_020359	2.84	9.38E-09	4.04E-08
BN1708_020384	3.41	1.72E-88	6.19E-87
BN1708_020422	2.49	1.09E-23	1.03E-22
BN1708_020436	2.75	2.11E-09	9.54E-09
BN1708_020455	2.52	1.30E-05	4.16E-05
BN1708_020468	3.73	5.99E-14	3.63E-13
BN1708_020493	2.41	1.29E-21	1.13E-20
BN1708_020494	2.83	6.80E-05	0.000198762
BN1708_020506	2.84	2.55E-58	5.45E-57
BN1708_020522	2.77	2.30E-13	1.35E-12
BN1708_020529	3.44	5.03E-12	2.74E-11
BN1708_020549	2.14	2.34E-05	7.22E-05
BN1708_020561	2.44	6.59E-10	3.10E-09
BN1708_020572	2.47	4.77E-16	3.20E-15
BN1708_020597	2.66	1.74E-42	2.75E-41
BN1708_020645	2.38	5.34E-17	3.75E-16
BN1708_020649	2.51	1.29E-27	1.40E-26
BN1708_020670	2.75	1.91E-99	8.03E-98
BN1708_020717	2.05	7.79E-13	4.46E-12
BN1708_020734	2.93	2.76E-17	1.97E-16
BN1708_020760	3.38	3.42E-204	5.82E-202
BN1708_020762	2.43	5.39E-15	3.44E-14
BN1708_020763	2.29	1.07E-38	1.56E-37
BN1708_020781	2.26	7.29E-30	8.38E-29
BN1708_020788	2.54	1.01E-36	1.41E-35
BN1708_020789	3.71	5.43E-38	7.74E-37

Table S3: List of *Verticillium* genes most down-regulated after 120 min of co-cultivation with *Pseudomonas protegens* P_DAPG. The reads of *V. longisporum* Vl43 have been mapped to the *V. longisporum* VL1 genome. Most down-regulated genes with Log_2-fold-change < -2 and p < 0.0001 have been chosen for further analysis.

Identifier	Log_2-fold-change	P-value	Adjusted p-value
BN1708_000023	-2.59	1.75E-07	6.76E-07
BN1708_000046	-2.73	3.90E-05	0.000117521
BN1708_000067	-2.60	4.09E-05	0.000123126
BN1708_000068	-2.12	2.29E-15	1.49E-14
BN1708_000074	-2.34	9.34E-15	5.89E-14
BN1708_000101	-2.25	4.72E-24	4.50E-23
BN1708_000234	-2.48	6.53E-64	1.54E-62
BN1708_000248	-2.29	1.14E-38	1.66E-37
BN1708_000257	-2.90	1.67E-11	8.82E-11
BN1708_000258	-2.56	5.83E-31	6.90E-30
BN1708_000274	-2.58	4.05E-06	1.36E-05
BN1708_000420	-2.01	1.59E-24	1.55E-23
BN1708_000462	-2.75	3.67E-13	2.14E-12
BN1708_000464	-3.43	2.19E-12	1.22E-11
BN1708_000478	-2.18	5.05E-15	3.23E-14
BN1708_000522	-3.00	6.61E-06	2.18E-05
BN1708_000541	-2.42	5.67E-05	0.000167435
BN1708_000561	-2.83	1.01E-07	3.99E-07
BN1708_000583	-2.25	4.03E-09	1.79E-08
BN1708_000621	-2.49	1.41E-15	9.29E-15
BN1708_000629	-2.04	1.29E-39	1.93E-38
BN1708_000693	-2.38	1.58E-34	2.07E-33
BN1708_000706	-2.47	1.11E-09	5.13E-09
BN1708_000714	-2.44	1.56E-08	6.62E-08
BN1708_000723	-2.07	2.86E-58	6.08E-57
BN1708_000724	-2.13	3.45E-19	2.70E-18
BN1708_000736	-2.19	5.68E-09	2.49E-08
BN1708_000739	-2.41	2.40E-09	1.08E-08
BN1708_000753	-2.05	2.04E-07	7.80E-07
BN1708_000840	-2.68	2.12E-19	1.68E-18
BN1708_000843	-2.24	3.59E-19	2.81E-18
BN1708_000854	-2.77	3.31E-20	2.71E-19
BN1708_000867	-3.30	2.22E-34	2.90E-33
BN1708_000912	-2.78	5.23E-16	3.50E-15
BN1708_000914	-2.23	4.19E-16	2.82E-15
BN1708_000920	-2.25	2.25E-39	3.32E-38
BN1708_000924	-2.34	8.74E-09	3.78E-08
BN1708_000936	-3.48	2.09E-09	9.45E-09

**Table S3: List of *Verticillium* genes most down-regulated after 120 min of co-cultivation with *Pseudomonas protegens* P_DAPG, continued.**			
**Identifier**	**Log_2-fold-change**	**P-value**	**Adjusted p-value**
BN1708_000949	-3.56	5.22E-38	7.45E-37
BN1708_000950	-3.68	1.28E-70	3.38E-69
BN1708_000991	-2.33	4.21E-20	3.44E-19
BN1708_000999	-2.73	1.33E-05	4.25E-05
BN1708_001043	-2.15	1.13E-08	4.85E-08
BN1708_001073	-2.71	1.13E-11	6.02E-11
BN1708_001085	-2.09	1.15E-06	4.11E-06
BN1708_001132	-2.15	4.57E-05	0.000136777
BN1708_001137	-2.07	1.91E-36	2.64E-35
BN1708_001190	-2.49	1.37E-15	9.02E-15
BN1708_001196	-2.16	4.49E-70	1.18E-68
BN1708_001217	-2.71	1.60E-17	1.16E-16
BN1708_001280	-2.09	1.50E-43	2.42E-42
BN1708_001332	-2.21	5.14E-14	3.13E-13
BN1708_001382	-2.35	1.76E-32	2.19E-31
BN1708_001396	-2.22	5.43E-13	3.14E-12
BN1708_001409	-2.01	2.01E-23	1.87E-22
BN1708_001428	-2.48	1.72E-07	6.63E-07
BN1708_001456	-2.06	5.34E-08	2.16E-07
BN1708_001485	-2.58	2.97E-08	1.23E-07
BN1708_001513	-2.10	8.43E-23	7.71E-22
BN1708_001516	-2.07	6.61E-05	0.000193302
BN1708_001519	-2.42	1.05E-05	3.39E-05
BN1708_001586	-2.57	1.11E-68	2.82E-67
BN1708_001628	-2.03	8.92E-09	3.85E-08
BN1708_001712	-3.43	5.41E-32	6.62E-31
BN1708_001719	-2.11	1.92E-10	9.43E-10
BN1708_001793	-2.56	5.49E-61	1.23E-59
BN1708_001794	-2.58	6.70E-29	7.49E-28
BN1708_001825	-2.02	8.32E-08	3.30E-07
BN1708_001839	-2.53	3.82E-05	0.000115464
BN1708_001876	-2.05	1.10E-05	3.54E-05
BN1708_001935	-2.28	2.08E-09	9.44E-09
BN1708_001983	-2.12	5.66E-13	3.27E-12
BN1708_002023	-2.18	4.49E-09	1.98E-08
BN1708_002025	-2.49	1.09E-24	1.07E-23
BN1708_002075	-2.87	2.73E-15	1.77E-14
BN1708_002112	-2.12	6.60E-13	3.80E-12
BN1708_002113	-2.63	5.04E-18	3.73E-17
BN1708_002136	-2.14	3.24E-09	1.45E-08
BN1708_002177	-2.30	7.92E-05	0.000229624

**Table S3: List of *Verticillium* genes most down-regulated after 120 min of co-cultivation with *Pseudomonas protegens* P_DAPG, continued.**			
**Identifier**	**Log_2-fold-change**	**P-value**	**Adjusted p-value**
BN1708_002201	-2.08	3.14E-46	5.38E-45
BN1708_002252	-2.31	6.37E-08	2.56E-07
BN1708_002253	-2.40	1.53E-07	5.93E-07
BN1708_002267	-3.32	1.22E-21	1.07E-20
BN1708_002272	-3.31	5.34E-67	1.32E-65
BN1708_002281	-2.20	1.97E-10	9.65E-10
BN1708_002301	-2.26	1.27E-05	4.06E-05
BN1708_002319	-2.98	1.99E-07	7.61E-07
BN1708_002333	-2.37	5.48E-11	2.80E-10
BN1708_002334	-2.04	6.59E-14	3.98E-13
BN1708_002346	-2.10	3.01E-06	1.02E-05
BN1708_002360	-2.96	4.35E-26	4.49E-25
BN1708_002361	-4.00	8.21E-138	5.61E-136
BN1708_002374	-2.59	1.02E-14	6.40E-14
BN1708_002384	-2.98	2.83E-24	2.73E-23
BN1708_002385	-2.08	1.23E-05	3.92E-05
BN1708_002448	-2.17	6.69E-06	2.20E-05
BN1708_002512	-2.66	7.73E-47	1.34E-45
BN1708_002614	-2.01	1.81E-09	8.24E-09
BN1708_002619	-2.03	5.47E-12	2.98E-11
BN1708_002627	-2.05	1.10E-05	3.54E-05
BN1708_002701	-2.50	1.77E-07	6.83E-07
BN1708_002780	-2.82	5.85E-19	4.51E-18
BN1708_002786	-2.30	3.00E-08	1.24E-07
BN1708_002849	-3.50	1.38E-146	1.07E-144
BN1708_002851	-2.69	1.84E-12	1.03E-11
BN1708_002885	-2.92	1.11E-28	1.24E-27
BN1708_002908	-2.04	4.28E-05	0.00012839
BN1708_002909	-2.66	6.16E-12	3.34E-11
BN1708_002910	-2.89	2.24E-12	1.25E-11
BN1708_002911	-2.93	3.83E-06	1.29E-05
BN1708_002917	-2.51	7.59E-09	3.29E-08
BN1708_002943	-2.07	6.52E-63	1.51E-61
BN1708_002947	-2.30	7.56E-14	4.56E-13
BN1708_002948	-2.21	5.23E-11	2.68E-10
BN1708_002964	-2.22	1.50E-13	8.92E-13
BN1708_002965	-2.78	9.07E-07	3.27E-06
BN1708_002989	-2.06	1.24E-23	1.16E-22
BN1708_002991	-2.23	4.74E-07	1.76E-06
BN1708_002994	-2.24	6.55E-06	2.16E-05

**Table S3: List of *Verticillium* genes most down-regulated after 120 min of co-cultivation with *Pseudomonas protegens* P_DAPG, continued.**			
**Identifier**	**Log_2-fold-change**	**P-value**	**Adjusted p-value**
BN1708_003029	-2.05	2.56E-11	1.34E-10
BN1708_003038	-2.78	1.85E-05	5.80E-05
BN1708_003057	-2.70	1.93E-07	7.41E-07
BN1708_003066	-4.35	3.47E-11	1.80E-10
BN1708_003067	-2.79	3.36E-06	1.14E-05
BN1708_003079	-3.28	9.40E-60	2.04E-58
BN1708_003082	-3.14	2.23E-05	6.91E-05
BN1708_003117	-2.03	1.89E-06	6.58E-06
BN1708_003160	-3.61	1.57E-05	4.97E-05
BN1708_003161	-2.73	1.12E-132	7.08E-131
BN1708_003164	-2.79	4.76E-161	4.45E-159
BN1708_003181	-2.11	9.69E-31	1.14E-29
BN1708_003183	-2.36	1.24E-27	1.34E-26
BN1708_003203	-2.05	4.14E-16	2.79E-15
BN1708_003223	-2.06	2.46E-28	2.71E-27
BN1708_003305	-2.07	4.66E-35	6.21E-34
BN1708_003322	-2.88	3.04E-32	3.75E-31
BN1708_003333	-2.08	4.11E-18	3.05E-17
BN1708_003339	-2.72	8.52E-30	9.75E-29
BN1708_003344	-2.50	5.32E-06	1.77E-05
BN1708_003353	-2.03	6.71E-35	8.89E-34
BN1708_003359	-3.41	6.88E-298	3.97E-295
BN1708_003364	-2.71	3.07E-32	3.79E-31
BN1708_003366	-2.37	1.77E-36	2.46E-35
BN1708_003398	-2.09	1.45E-09	6.65E-09
BN1708_003427	-2.89	9.03E-06	2.93E-05
BN1708_003441	-2.39	3.12E-16	2.11E-15
BN1708_003452	-2.48	1.69E-10	8.32E-10
BN1708_003463	-2.31	1.48E-06	5.24E-06
BN1708_003537	-2.78	8.08E-24	7.65E-23
BN1708_003542	-2.11	3.34E-05	0.000101311
BN1708_003545	-2.53	4.26E-16	2.87E-15
BN1708_003576	-2.68	1.86E-08	7.82E-08
BN1708_003625	-3.33	8.68E-07	3.14E-06
BN1708_003626	-3.68	2.77E-10	1.34E-09
BN1708_003654	-2.47	3.28E-24	3.15E-23
BN1708_003655	-2.96	1.58E-09	7.24E-09
BN1708_003675	-2.86	9.86E-35	1.30E-33
BN1708_003681	-2.16	1.19E-11	6.33E-11
BN1708_003686	-2.01	4.89E-06	1.63E-05
BN1708_003698	-4.87	5.71E-101	2.44E-99

**Table S3: List of *Verticillium* genes most down-regulated after 120 min of co-cultivation with *Pseudomonas protegens* P_DAPG, continued.**			
**Identifier**	**Log_2-fold-change**	**P-value**	**Adjusted p-value**
BN1708_003700	-4.66	7.71E-162	7.28E-160
BN1708_003703	-4.43	9.11E-26	9.30E-25
BN1708_003704	-4.07	1.27E-29	1.45E-28
BN1708_003705	-2.48	3.77E-10	1.81E-09
BN1708_003758	-2.47	3.08E-05	9.38E-05
BN1708_003781	-4.19	1.52E-11	8.04E-11
BN1708_003787	-3.42	6.15E-05	0.000180748
BN1708_003788	-2.67	1.43E-28	1.58E-27
BN1708_003791	-2.69	7.44E-10	3.48E-09
BN1708_003793	-3.00	6.75E-10	3.17E-09
BN1708_003794	-3.00	1.11E-38	1.61E-37
BN1708_003807	-2.11	1.40E-15	9.22E-15
BN1708_003808	-2.35	3.69E-30	4.28E-29
BN1708_003816	-2.41	9.51E-11	4.77E-10
BN1708_003821	-2.03	9.88E-20	7.91E-19
BN1708_003866	-2.64	7.56E-11	3.82E-10
BN1708_003867	-2.33	5.36E-15	3.42E-14
BN1708_003874	-2.15	3.67E-16	2.48E-15
BN1708_003889	-4.03	8.41E-06	2.74E-05
BN1708_003970	-2.77	1.46E-08	6.21E-08
BN1708_004005	-2.08	1.28E-07	5.01E-07
BN1708_004038	-2.91	4.51E-15	2.89E-14
BN1708_004043	-3.33	1.11E-260	3.72E-258
BN1708_004051	-2.03	2.37E-09	1.07E-08
BN1708_004081	-2.16	1.40E-09	6.44E-09
BN1708_004083	-2.25	1.10E-10	5.47E-10
BN1708_004093	-3.90	1.07E-10	5.36E-10
BN1708_004094	-2.65	3.91E-11	2.02E-10
BN1708_004112	-2.14	3.69E-14	2.27E-13
BN1708_004119	-2.41	1.23E-09	5.68E-09
BN1708_004165	-2.13	2.19E-13	1.29E-12
BN1708_004181	-2.09	1.64E-08	6.96E-08
BN1708_004218	-2.82	8.37E-05	0.000241748
BN1708_004242	-3.37	4.82E-08	1.96E-07
BN1708_004244	-2.20	2.99E-10	1.45E-09
BN1708_004254	-2.78	2.33E-41	3.62E-40
BN1708_004438	-2.46	1.21E-27	1.31E-26
BN1708_004481	-2.28	2.13E-05	6.61E-05
BN1708_004495	-2.03	1.74E-06	6.09E-06
BN1708_004552	-2.01	1.89E-06	6.60E-06

**Table S3: List of *Verticillium* genes most down-regulated after 120 min of co-cultivation with *Pseudomonas protegens* P_DAPG, continued.**			
**Identifier**	**Log_2-fold-change**	**P-value**	**Adjusted p-value**
BN1708_004616	-2.17	3.02E-09	1.36E-08
BN1708_004657	-2.26	3.84E-05	0.000115816
BN1708_004687	-2.10	1.61E-11	8.50E-11
BN1708_004696	-2.34	2.03E-31	2.44E-30
BN1708_004698	-2.22	3.38E-07	1.26E-06
BN1708_004770	-2.85	3.16E-07	1.19E-06
BN1708_004773	-2.45	7.30E-06	2.39E-05
BN1708_004775	-2.85	3.27E-80	1.02E-78
BN1708_004778	-4.10	4.31E-102	1.87E-100
BN1708_004789	-3.57	1.84E-54	3.70E-53
BN1708_004889	-2.54	1.11E-12	6.30E-12
BN1708_004937	-2.38	3.50E-37	4.92E-36
BN1708_004938	-2.74	1.77E-10	8.67E-10
BN1708_004975	-2.35	1.17E-11	6.24E-11
BN1708_005085	-2.41	1.78E-11	9.42E-11
BN1708_005191	-2.78	1.42E-07	5.53E-07
BN1708_005199	-2.30	1.35E-08	5.74E-08
BN1708_005208	-2.56	8.64E-32	1.05E-30
BN1708_005217	-2.19	1.83E-21	1.59E-20
BN1708_005234	-2.07	5.80E-16	3.87E-15
BN1708_005275	-2.41	3.87E-09	1.72E-08
BN1708_005301	-3.60	5.80E-08	2.34E-07
BN1708_005376	-3.21	5.03E-05	0.00014965
BN1708_005382	-2.17	3.41E-05	0.00010354
BN1708_005401	-2.33	2.02E-15	1.32E-14
BN1708_005423	-2.32	1.46E-05	4.63E-05
BN1708_005427	-2.48	6.20E-11	3.15E-10
BN1708_005430	-2.20	2.71E-05	8.30E-05
BN1708_005507	-2.26	7.66E-25	7.56E-24
BN1708_005508	-2.93	1.27E-33	1.63E-32
BN1708_005558	-2.09	5.10E-21	4.32E-20
BN1708_005559	-2.05	5.79E-13	3.35E-12
BN1708_005573	-2.36	3.03E-27	3.25E-26
BN1708_005600	-2.33	6.81E-09	2.96E-08
BN1708_005601	-2.11	1.46E-10	7.20E-10
BN1708_005661	-2.29	8.71E-13	4.97E-12
BN1708_005662	-2.01	1.07E-11	5.74E-11
BN1708_005666	-2.01	5.71E-15	3.64E-14
BN1708_005703	-2.20	4.78E-24	4.55E-23
BN1708_005722	-2.01	6.60E-05	0.000193258
BN1708_005724	-2.09	3.60E-30	4.18E-29

**Table S3: List of *Verticillium* genes most down-regulated after 120 min of co-cultivation with *Pseudomonas protegens* P_DAPG, continued.**			
**Identifier**	**Log_2-fold-change**	**P-value**	**Adjusted p-value**
BN1708_005725	-2.14	1.78E-32	2.21E-31
BN1708_005727	-2.46	2.23E-67	5.56E-66
BN1708_005740	-2.07	6.39E-20	5.17E-19
BN1708_005785	-2.08	7.71E-12	4.15E-11
BN1708_005804	-2.26	4.93E-06	1.65E-05
BN1708_005808	-2.21	1.14E-08	4.87E-08
BN1708_005892	-3.61	1.54E-35	2.08E-34
BN1708_005905	-2.43	2.26E-91	8.45E-90
BN1708_005931	-3.51	4.90E-46	8.37E-45
BN1708_005932	-2.77	2.00E-54	4.01E-53
BN1708_005947	-2.02	1.10E-49	2.04E-48
BN1708_005980	-2.11	1.31E-16	9.06E-16
BN1708_006011	-2.12	6.99E-05	0.000203976
BN1708_006040	-2.06	4.26E-11	2.19E-10
BN1708_006061	-2.68	1.04E-30	1.22E-29
BN1708_006106	-2.99	6.78E-27	7.18E-26
BN1708_006156	-2.36	1.11E-37	1.58E-36
BN1708_006185	-3.06	7.89E-28	8.59E-27
BN1708_006205	-2.02	1.10E-05	3.54E-05
BN1708_006285	-2.09	1.41E-25	1.43E-24
BN1708_006313	-2.19	1.43E-38	2.07E-37
BN1708_006333	-2.55	2.11E-32	2.61E-31
BN1708_006338	-3.00	2.11E-06	7.32E-06
BN1708_006363	-2.65	2.16E-13	1.27E-12
BN1708_006385	-2.42	9.82E-46	1.67E-44
BN1708_006492	-2.14	1.91E-05	5.99E-05
BN1708_006537	-2.58	1.48E-15	9.73E-15
BN1708_006538	-2.82	1.62E-06	5.71E-06
BN1708_006551	-2.02	1.80E-07	6.92E-07
BN1708_006597	-2.30	1.10E-06	3.93E-06
BN1708_006651	-2.45	1.05E-09	4.89E-09
BN1708_006710	-3.05	1.34E-05	4.26E-05
BN1708_006714	-2.02	7.37E-25	7.27E-24
BN1708_006728	-2.05	9.37E-26	9.55E-25
BN1708_006742	-2.66	9.12E-07	3.29E-06
BN1708_006749	-2.56	8.85E-14	5.32E-13
BN1708_006771	-2.18	6.29E-28	6.85E-27
BN1708_006875	-2.81	1.43E-08	6.07E-08
BN1708_006947	-2.19	5.62E-05	0.000165928
BN1708_006995	-2.11	9.04E-09	3.90E-08

**Table S3: List of *Verticillium* genes most down-regulated after 120 min of co-cultivation with *Pseudomonas protegens* P_DAPG, continued.**			
**Identifier**	**Log_2-fold-change**	**P-value**	**Adjusted p-value**
BN1708_007041	-2.20	6.76E-19	5.19E-18
BN1708_007089	-2.08	1.23E-20	1.03E-19
BN1708_007094	-2.62	1.53E-38	2.21E-37
BN1708_007130	-2.39	1.03E-10	5.15E-10
BN1708_007137	-2.38	1.03E-09	4.80E-09
BN1708_007144	-3.39	1.27E-32	1.58E-31
BN1708_007175	-2.53	4.30E-21	3.66E-20
BN1708_007217	-5.42	2.55E-21	2.19E-20
BN1708_007283	-2.27	4.39E-23	4.04E-22
BN1708_007289	-2.43	1.13E-18	8.60E-18
BN1708_007358	-2.03	9.17E-08	3.63E-07
BN1708_007364	-2.40	5.65E-27	6.02E-26
BN1708_007462	-3.19	5.86E-30	6.75E-29
BN1708_007497	-2.03	3.09E-05	9.44E-05
BN1708_007502	-2.45	8.35E-13	4.77E-12
BN1708_007518	-2.34	2.96E-06	1.01E-05
BN1708_007526	-2.60	8.24E-82	2.68E-80
BN1708_007528	-2.15	2.10E-06	7.29E-06
BN1708_007574	-2.16	5.78E-09	2.53E-08
BN1708_007608	-2.55	2.33E-21	2.01E-20
BN1708_007610	-2.96	2.79E-20	2.29E-19
BN1708_007615	-2.23	5.27E-08	2.14E-07
BN1708_007738	-2.28	1.82E-05	5.71E-05
BN1708_007755	-2.11	5.52E-19	4.27E-18
BN1708_007766	-3.82	4.06E-09	1.80E-08
BN1708_007779	-2.83	4.86E-06	1.63E-05
BN1708_007789	-2.14	4.48E-08	1.83E-07
BN1708_007838	-2.90	4.94E-06	1.65E-05
BN1708_007843	-2.67	1.88E-28	2.07E-27
BN1708_007913	-2.04	1.21E-10	6.03E-10
BN1708_007927	-2.14	2.49E-06	8.55E-06
BN1708_007930	-3.88	4.58E-21	3.89E-20
BN1708_007935	-3.27	6.19E-19	4.77E-18
BN1708_008011	-2.92	8.35E-10	3.90E-09
BN1708_008029	-3.16	1.08E-19	8.62E-19
BN1708_008033	-4.35	3.84E-43	6.15E-42
BN1708_008041	-2.18	6.46E-08	2.59E-07
BN1708_008088	-2.04	5.74E-09	2.52E-08
BN1708_008133	-4.08	1.41E-29	1.61E-28
BN1708_008139	-3.47	1.04E-28	1.16E-27
BN1708_008146	-2.25	9.76E-11	4.89E-10

**Table S3: List of *Verticillium* genes most down-regulated after 120 min of co-cultivation with *Pseudomonas protegens* P_DAPG, continued.**			
**Identifier**	**Log_2-fold-change**	**P-value**	**Adjusted p-value**
BN1708_008177	-2.25	6.10E-19	4.70E-18
BN1708_008232	-2.66	3.80E-06	1.28E-05
BN1708_008244	-2.23	8.61E-32	1.05E-30
BN1708_008254	-2.85	2.49E-12	1.38E-11
BN1708_008288	-2.01	2.23E-21	1.93E-20
BN1708_008289	-2.42	1.05E-21	9.27E-21
BN1708_008298	-2.39	4.85E-07	1.79E-06
BN1708_008350	-2.34	3.25E-06	1.11E-05
BN1708_008414	-2.18	8.31E-13	4.75E-12
BN1708_008428	-2.14	1.01E-33	1.30E-32
BN1708_008442	-2.53	9.37E-11	4.70E-10
BN1708_008447	-2.05	3.69E-30	4.28E-29
BN1708_008525	-2.66	8.85E-11	4.45E-10
BN1708_008560	-2.39	3.18E-11	1.65E-10
BN1708_008587	-2.10	7.29E-21	6.12E-20
BN1708_008596	-2.11	4.47E-12	2.45E-11
BN1708_008704	-2.09	1.69E-08	7.14E-08
BN1708_008707	-2.55	4.07E-07	1.52E-06
BN1708_008755	-2.38	3.63E-05	0.000109728
BN1708_008757	-2.07	9.97E-14	5.99E-13
BN1708_008759	-2.38	1.57E-10	7.73E-10
BN1708_008804	-2.34	5.21E-05	0.000154574
BN1708_008806	-2.16	1.21E-09	5.59E-09
BN1708_008815	-2.27	8.52E-05	0.000245837
BN1708_008819	-2.22	3.42E-36	4.69E-35
BN1708_008829	-4.00	5.46E-08	2.21E-07
BN1708_008832	-2.57	1.00E-05	3.24E-05
BN1708_008833	-3.91	1.21E-93	4.72E-92
BN1708_008834	-2.63	1.50E-38	2.17E-37
BN1708_008835	-4.50	1.37E-57	2.89E-56
BN1708_008846	-2.14	1.56E-07	6.05E-07
BN1708_008922	-2.31	3.40E-36	4.66E-35
BN1708_008923	-2.43	1.15E-07	4.52E-07
BN1708_008996	-2.03	5.81E-25	5.76E-24
BN1708_009068	-2.61	2.67E-140	1.86E-138
BN1708_009069	-2.59	3.16E-137	2.13E-135
BN1708_009097	-2.03	3.75E-21	3.20E-20
BN1708_009098	-2.03	6.34E-27	6.73E-26
BN1708_009108	-2.15	1.03E-32	1.28E-31
BN1708_009109	-2.12	3.00E-70	7.88E-69

**Table S3: List of *Verticillium* genes most down-regulated after 120 min of co-cultivation with *Pseudomonas protegens* P_DAPG, continued.**			
**Identifier**	**Log_2-fold-change**	**P-value**	**Adjusted p-value**
BN1708_009126	-2.35	2.93E-21	2.51E-20
BN1708_009163	-3.22	8.11E-154	6.87E-152
BN1708_009243	-2.14	8.13E-07	2.95E-06
BN1708_009245	-2.71	1.25E-19	9.93E-19
BN1708_009266	-3.40	1.63E-10	8.03E-10
BN1708_009267	-2.46	2.89E-10	1.40E-09
BN1708_009268	-2.72	3.38E-07	1.26E-06
BN1708_009333	-2.19	4.94E-05	0.000147133
BN1708_009334	-2.48	2.59E-07	9.79E-07
BN1708_009394	-2.43	1.40E-10	6.94E-10
BN1708_009397	-2.29	9.03E-06	2.94E-05
BN1708_009405	-2.11	6.20E-05	0.000182088
BN1708_009418	-2.36	1.23E-08	5.26E-08
BN1708_009483	-2.45	7.66E-09	3.32E-08
BN1708_009556	-3.65	4.12E-14	2.52E-13
BN1708_009557	-2.30	4.80E-43	7.66E-42
BN1708_009576	-2.27	3.10E-12	1.71E-11
BN1708_009579	-2.50	1.36E-12	7.66E-12
BN1708_009586	-2.80	1.32E-17	9.55E-17
BN1708_009590	-2.54	2.49E-37	3.50E-36
BN1708_009612	-2.41	8.40E-12	4.52E-11
BN1708_009630	-2.12	6.27E-08	2.52E-07
BN1708_009631	-3.10	3.80E-66	9.27E-65
BN1708_009635	-3.32	1.16E-12	6.56E-12
BN1708_009638	-2.43	5.72E-05	0.000168843
BN1708_009662	-2.12	4.72E-07	1.75E-06
BN1708_009694	-2.91	1.24E-19	9.88E-19
BN1708_009696	-2.06	2.63E-07	9.93E-07
BN1708_009722	-2.13	1.34E-05	4.27E-05
BN1708_009870	-2.58	3.64E-18	2.70E-17
BN1708_009910	-2.13	1.45E-05	4.61E-05
BN1708_009985	-2.38	7.09E-07	2.59E-06
BN1708_009997	-3.13	9.09E-09	3.92E-08
BN1708_010051	-3.73	2.31E-10	1.13E-09
BN1708_010074	-2.12	1.76E-06	6.17E-06
BN1708_010077	-2.48	5.99E-11	3.05E-10
BN1708_010166	-2.20	2.72E-43	4.38E-42
BN1708_010187	-2.19	2.49E-10	1.21E-09
BN1708_010224	-2.38	2.75E-05	8.41E-05
BN1708_010230	-2.63	4.93E-07	1.82E-06
BN1708_010305	-2.16	9.34E-07	3.37E-06

**Table S3: List of *Verticillium* genes most down-regulated after 120 min of co-cultivation with *Pseudomonas protegens* P_DAPG, continued.**			
**Identifier**	**Log$_2$-fold-change**	**P-value**	**Adjusted p-value**
BN1708_010326	-3.16	9.65E-20	7.74E-19
BN1708_010390	-2.03	1.23E-30	1.44E-29
BN1708_010408	-2.48	1.62E-05	5.12E-05
BN1708_010423	-2.45	2.19E-44	3.59E-43
BN1708_010426	-2.07	7.79E-18	5.71E-17
BN1708_010428	-2.83	2.33E-48	4.16E-47
BN1708_010431	-3.19	4.72E-14	2.88E-13
BN1708_010446	-2.17	6.44E-05	0.000188584
BN1708_010464	-2.37	2.18E-09	9.85E-09
BN1708_010468	-2.21	1.20E-13	7.14E-13
BN1708_010485	-2.20	3.58E-10	1.72E-09
BN1708_010487	-2.06	1.12E-10	5.58E-10
BN1708_010617	-3.08	2.44E-17	1.75E-16
BN1708_010709	-2.93	1.75E-163	1.70E-161
BN1708_010713	-2.73	6.66E-117	3.49E-115
BN1708_010715	-2.02	9.08E-16	6.01E-15
BN1708_010754	-3.46	2.09E-21	1.81E-20
BN1708_010757	-2.89	4.89E-20	3.97E-19
BN1708_010758	-2.80	5.85E-06	1.94E-05
BN1708_010761	-2.78	2.26E-66	5.55E-65
BN1708_010762	-2.14	9.62E-17	6.68E-16
BN1708_010771	-2.36	4.69E-10	2.23E-09
BN1708_010772	-2.14	1.76E-14	1.09E-13
BN1708_010807	-2.09	2.01E-07	7.68E-07
BN1708_010888	-2.26	6.54E-05	0.000191353
BN1708_010940	-2.65	2.39E-44	3.92E-43
BN1708_010966	-3.54	5.26E-06	1.75E-05
BN1708_011029	-2.75	6.96E-07	2.54E-06
BN1708_011030	-2.60	5.77E-14	3.50E-13
BN1708_011031	-2.06	1.13E-06	4.03E-06
BN1708_011033	-2.36	1.94E-10	9.49E-10
BN1708_011050	-2.73	1.50E-28	1.66E-27
BN1708_011079	-2.63	8.88E-69	2.27E-67
BN1708_011080	-2.28	1.59E-79	4.89E-78
BN1708_011148	-3.08	2.36E-07	8.98E-07
BN1708_011164	-2.01	1.85E-06	6.46E-06
BN1708_011256	-2.15	1.28E-13	7.63E-13
BN1708_011278	-2.17	3.37E-15	2.17E-14
BN1708_011311	-2.05	1.01E-12	5.77E-12
BN1708_011340	-2.30	3.46E-24	3.32E-23

**Table S3: List of *Verticillium* genes most down-regulated after 120 min of co-cultivation with *Pseudomonas protegens* P_DAPG, continued.**			
**Identifier**	**Log_2-fold-change**	**P-value**	**Adjusted p-value**
BN1708_011367	-2.45	2.26E-14	1.40E-13
BN1708_011379	-2.05	1.61E-92	6.16E-91
BN1708_011411	-2.04	5.11E-07	1.89E-06
BN1708_011447	-2.68	1.07E-19	8.57E-19
BN1708_011453	-2.18	1.47E-18	1.11E-17
BN1708_011459	-2.54	1.82E-13	1.08E-12
BN1708_011465	-2.51	4.27E-06	1.43E-05
BN1708_011472	-2.67	1.77E-09	8.06E-09
BN1708_011650	-2.82	1.40E-22	1.26E-21
BN1708_011755	-4.25	2.25E-61	5.09E-60
BN1708_011756	-3.77	1.11E-31	1.34E-30
BN1708_011772	-4.53	3.33E-20	2.72E-19
BN1708_011773	-3.99	5.95E-24	5.66E-23
BN1708_011892	-3.30	4.06E-26	4.19E-25
BN1708_011917	-2.62	6.87E-05	0.000200702
BN1708_011947	-5.64	1.44E-31	1.74E-30
BN1708_011963	-2.32	2.46E-05	7.58E-05
BN1708_012016	-2.99	2.24E-05	6.93E-05
BN1708_012018	-2.86	1.50E-24	1.46E-23
BN1708_012031	-2.84	1.55E-06	5.47E-06
BN1708_012060	-2.04	6.35E-05	0.000186365
BN1708_012092	-2.71	9.11E-137	6.14E-135
BN1708_012117	-2.58	4.55E-07	1.69E-06
BN1708_012259	-5.27	6.57E-27	6.96E-26
BN1708_012277	-2.08	1.54E-48	2.77E-47
BN1708_012309	-2.45	1.42E-20	1.19E-19
BN1708_012321	-2.59	9.63E-24	9.10E-23
BN1708_012330	-2.81	2.64E-14	1.63E-13
BN1708_012483	-2.66	6.06E-06	2.01E-05
BN1708_012492	-2.01	1.42E-12	7.98E-12
BN1708_012503	-2.11	4.15E-05	0.000124821
BN1708_012548	-3.74	4.58E-17	3.23E-16
BN1708_012554	-2.50	1.50E-07	5.81E-07
BN1708_012614	-2.43	5.00E-07	1.85E-06
BN1708_012621	-2.43	9.01E-10	4.19E-09
BN1708_012623	-2.26	2.09E-07	7.99E-07
BN1708_012626	-2.77	2.17E-14	1.35E-13
BN1708_012633	-2.09	8.86E-07	3.20E-06
BN1708_012637	-3.95	4.12E-13	2.40E-12
BN1708_012694	-2.31	1.41E-07	5.50E-07
BN1708_012745	-3.19	1.45E-05	4.61E-05

Table S3: List of *Verticillium* genes most down-regulated after 120 min of co-cultivation with *Pseudomonas protegens* P_DAPG, continued.

Identifier	Log_2-fold-change	P-value	Adjusted p-value
BN1708_012867	-2.09	5.08E-40	7.68E-39
BN1708_012880	-2.87	5.75E-10	2.72E-09
BN1708_012881	-3.98	2.75E-27	2.95E-26
BN1708_012882	-4.44	1.92E-24	1.86E-23
BN1708_012889	-2.22	1.67E-10	8.24E-10
BN1708_012905	-2.24	3.39E-09	1.51E-08
BN1708_012911	-2.42	1.23E-09	5.70E-09
BN1708_012912	-2.84	1.36E-05	4.33E-05
BN1708_012914	-2.53	1.92E-10	9.42E-10
BN1708_012932	-2.05	2.00E-05	6.23E-05
BN1708_012940	-2.52	2.64E-22	2.37E-21
BN1708_012941	-2.18	2.89E-23	2.68E-22
BN1708_012942	-2.09	4.08E-20	3.33E-19
BN1708_012953	-2.77	2.14E-05	6.65E-05
BN1708_012963	-2.71	5.96E-06	1.98E-05
BN1708_013033	-2.35	1.80E-14	1.12E-13
BN1708_013035	-4.36	2.14E-05	6.65E-05
BN1708_013068	-3.89	4.70E-08	1.91E-07
BN1708_013096	-2.43	8.62E-07	3.12E-06
BN1708_013111	-2.62	1.02E-45	1.73E-44
BN1708_013124	-3.75	6.13E-27	6.50E-26
BN1708_013125	-3.01	1.14E-27	1.24E-26
BN1708_013138	-2.77	6.17E-50	1.16E-48
BN1708_013198	-2.35	6.02E-06	2.00E-05
BN1708_013307	-2.35	1.47E-07	5.70E-07
BN1708_013315	-2.30	6.90E-11	3.49E-10
BN1708_013330	-2.16	7.09E-06	2.33E-05
BN1708_013381	-2.61	3.69E-16	2.49E-15
BN1708_013394	-2.47	4.50E-06	1.51E-05
BN1708_013510	-2.05	2.27E-16	1.55E-15
BN1708_013533	-2.81	4.65E-21	3.94E-20
BN1708_013553	-2.69	2.05E-08	8.60E-08
BN1708_013582	-2.21	1.46E-05	4.63E-05
BN1708_013583	-2.89	7.37E-09	3.20E-08
BN1708_013617	-2.28	8.59E-05	0.000247689
BN1708_013687	-2.32	1.41E-08	6.00E-08
BN1708_013715	-2.93	1.40E-07	5.45E-07
BN1708_013722	-2.47	3.15E-33	4.00E-32
BN1708_013723	-2.52	3.17E-28	3.47E-27
BN1708_013724	-3.21	2.38E-11	1.25E-10

**Table S3: List of *Verticillium* genes most down-regulated after 120 min of co-cultivation with *Pseudomonas protegens* P_DAPG, continued.**			
**Identifier**	**Log_2-fold-change**	**P-value**	**Adjusted p-value**
BN1708_013757	-2.63	6.00E-09	2.62E-08
BN1708_013763	-2.01	6.93E-07	2.53E-06
BN1708_013772	-2.63	8.15E-07	2.95E-06
BN1708_013788	-4.14	1.02E-50	1.93E-49
BN1708_013842	-2.28	3.94E-19	3.07E-18
BN1708_013844	-2.12	1.05E-12	5.94E-12
BN1708_013853	-3.22	9.44E-16	6.25E-15
BN1708_013864	-2.44	6.75E-44	1.10E-42
BN1708_013922	-2.68	3.35E-101	1.45E-99
BN1708_013923	-3.40	2.83E-74	7.97E-73
BN1708_013941	-2.81	6.82E-24	6.47E-23
BN1708_013958	-2.21	4.31E-16	2.90E-15
BN1708_013968	-2.38	3.21E-45	5.35E-44
BN1708_013985	-2.42	2.82E-38	4.05E-37
BN1708_013995	-2.51	5.92E-11	3.02E-10
BN1708_013997	-2.12	5.78E-08	2.33E-07
BN1708_014057	-2.22	2.47E-08	1.03E-07
BN1708_014081	-2.21	6.20E-05	0.000181969
BN1708_014092	-2.13	2.40E-09	1.08E-08
BN1708_014119	-2.63	4.10E-08	1.68E-07
BN1708_014161	-2.80	2.62E-24	2.53E-23
BN1708_014269	-2.97	3.80E-09	1.69E-08
BN1708_014301	-2.55	5.37E-49	9.78E-48
BN1708_014354	-3.28	1.03E-47	1.83E-46
BN1708_014370	-2.35	1.92E-11	1.01E-10
BN1708_014371	-2.08	1.02E-24	1.00E-23
BN1708_014435	-3.17	4.36E-06	1.46E-05
BN1708_014460	-2.12	9.10E-08	3.60E-07
BN1708_014485	-2.20	1.88E-17	1.36E-16
BN1708_014486	-2.66	2.49E-18	1.86E-17
BN1708_014500	-2.96	8.30E-06	2.71E-05
BN1708_014501	-3.62	2.39E-13	1.40E-12
BN1708_014506	-2.05	8.90E-11	4.47E-10
BN1708_014549	-2.66	7.36E-21	6.17E-20
BN1708_014552	-2.06	1.75E-09	7.99E-09
BN1708_014553	-2.07	3.36E-25	3.35E-24
BN1708_014565	-5.50	4.46E-180	5.35E-178
BN1708_014566	-2.27	9.65E-07	3.48E-06
BN1708_014579	-3.64	2.38E-13	1.40E-12
BN1708_014580	-3.02	1.89E-45	3.18E-44
BN1708_014583	-3.18	4.76E-06	1.59E-05

Table S3: List of *Verticillium* genes most down-regulated after 120 min of co-cultivation with *Pseudomonas protegens* P_DAPG, continued.

Identifier	Log_2-fold-change	P-value	Adjusted p-value
BN1708_014584	-4.10	6.38E-06	2.11E-05
BN1708_014594	-4.04	8.88E-06	2.89E-05
BN1708_014627	-3.43	9.97E-46	1.69E-44
BN1708_014628	-2.74	8.07E-101	3.43E-99
BN1708_014635	-2.88	1.97E-09	8.92E-09
BN1708_014641	-2.44	3.34E-31	4.00E-30
BN1708_014662	-2.13	2.08E-05	6.48E-05
BN1708_014675	-2.27	2.47E-06	8.49E-06
BN1708_014680	-2.13	2.46E-11	1.29E-10
BN1708_014688	-2.30	5.87E-12	3.18E-11
BN1708_014774	-3.07	1.69E-26	1.78E-25
BN1708_014796	-2.15	3.09E-10	1.49E-09
BN1708_014797	-2.09	1.14E-06	4.09E-06
BN1708_014807	-2.33	1.88E-21	1.63E-20
BN1708_014839	-2.91	3.82E-16	2.58E-15
BN1708_014870	-3.24	1.38E-45	2.33E-44
BN1708_014922	-4.14	9.29E-21	7.77E-20
BN1708_014933	-3.02	1.63E-05	5.15E-05
BN1708_014939	-3.92	8.50E-74	2.37E-72
BN1708_014940	-3.50	8.60E-69	2.20E-67
BN1708_014997	-2.17	1.96E-07	7.52E-07
BN1708_014998	-3.60	5.37E-233	1.30E-230
BN1708_015000	-2.58	5.69E-09	2.49E-08
BN1708_015003	-2.22	3.55E-13	2.07E-12
BN1708_015007	-2.09	6.97E-13	4.01E-12
BN1708_015009	-2.17	8.45E-06	2.75E-05
BN1708_015015	-3.31	6.71E-82	2.19E-80
BN1708_015056	-2.17	2.63E-12	1.46E-11
BN1708_015068	-2.05	1.64E-05	5.17E-05
BN1708_015082	-3.17	2.17E-51	4.14E-50
BN1708_015111	-2.51	6.40E-55	1.30E-53
BN1708_015118	-2.45	1.50E-06	5.30E-06
BN1708_015132	-2.36	3.17E-20	2.60E-19
BN1708_015133	-2.68	4.62E-19	3.59E-18
BN1708_015160	-2.36	2.03E-17	1.46E-16
BN1708_015228	-2.85	7.41E-09	3.22E-08
BN1708_015257	-2.57	1.42E-55	2.90E-54
BN1708_015266	-3.01	1.62E-06	5.71E-06
BN1708_015327	-3.31	2.99E-18	2.23E-17
BN1708_015375	-2.72	2.62E-17	1.87E-16

**Table S3: List of *Verticillium* genes most down-regulated after 120 min of co-cultivation with *Pseudomonas protegens* P_DAPG, continued.**			
**Identifier**	**Log_2-fold-change**	**P-value**	**Adjusted p-value**
BN1708_015376	-2.17	1.41E-12	7.95E-12
BN1708_015403	-2.21	8.66E-05	0.000249554
BN1708_015404	-2.22	5.49E-07	2.02E-06
BN1708_015424	-2.91	4.92E-06	1.65E-05
BN1708_015470	-2.23	1.34E-21	1.17E-20
BN1708_015477	-2.69	1.94E-05	6.05E-05
BN1708_015482	-2.93	5.51E-80	1.70E-78
BN1708_015489	-2.62	4.60E-15	2.94E-14
BN1708_015537	-2.42	2.25E-33	2.86E-32
BN1708_015556	-2.52	2.89E-20	2.38E-19
BN1708_015558	-3.43	7.28E-25	7.19E-24
BN1708_015583	-2.19	1.16E-07	4.56E-07
BN1708_015645	-3.75	4.18E-146	3.21E-144
BN1708_015646	-2.39	4.47E-05	0.000133841
BN1708_015649	-2.71	7.15E-11	3.62E-10
BN1708_015655	-2.79	5.78E-14	3.51E-13
BN1708_015656	-2.86	2.81E-08	1.17E-07
BN1708_015658	-2.60	1.51E-98	6.25E-97
BN1708_015661	-4.23	1.39E-117	7.35E-116
BN1708_015705	-2.49	1.23E-05	3.93E-05
BN1708_015942	-2.07	1.95E-05	6.10E-05
BN1708_015958	-2.40	3.90E-05	0.000117605
BN1708_016048	-2.74	3.79E-06	1.28E-05
BN1708_016055	-2.48	1.08E-13	6.48E-13
BN1708_016059	-3.70	1.46E-08	6.20E-08
BN1708_016114	-2.19	1.81E-07	6.97E-07
BN1708_016144	-2.19	6.79E-10	3.19E-09
BN1708_016235	-2.76	5.05E-18	3.73E-17
BN1708_016276	-3.53	5.88E-11	3.00E-10
BN1708_016297	-3.44	4.79E-222	9.82E-220
BN1708_016313	-2.60	5.17E-161	4.81E-159
BN1708_016316	-2.83	3.38E-11	1.75E-10
BN1708_016328	-2.07	4.87E-33	6.14E-32
BN1708_016384	-2.31	7.36E-07	2.68E-06
BN1708_016438	-2.14	1.05E-13	6.28E-13
BN1708_016482	-2.24	2.77E-15	1.79E-14
BN1708_016526	-2.80	1.00E-61	2.29E-60
BN1708_016596	-2.08	6.52E-11	3.31E-10
BN1708_016713	-2.31	2.18E-07	8.33E-07
BN1708_016719	-2.72	1.21E-11	6.47E-11
BN1708_016790	-2.34	2.22E-19	1.75E-18

Table S3: List of *Verticillium* genes most down-regulated after 120 min of co-cultivation with *Pseudomonas protegens* P_DAPG, continued.

Identifier	Log_2-fold-change	P-value	Adjusted p-value
BN1708_016817	-2.34	5.04E-22	4.50E-21
BN1708_016841	-2.29	1.02E-47	1.81E-46
BN1708_016843	-3.44	2.55E-23	2.37E-22
BN1708_016851	-2.34	1.81E-05	5.68E-05
BN1708_016852	-2.12	6.35E-31	7.51E-30
BN1708_016875	-2.80	9.68E-05	0.000276757
BN1708_016889	-2.64	2.79E-06	9.55E-06
BN1708_016921	-2.11	3.73E-06	1.26E-05
BN1708_016933	-3.32	4.65E-08	1.89E-07
BN1708_017002	-2.06	7.19E-05	0.000209373
BN1708_017053	-2.79	1.29E-10	6.43E-10
BN1708_017058	-2.11	6.44E-05	0.000188737
BN1708_017125	-2.19	9.07E-08	3.59E-07
BN1708_017167	-2.04	1.19E-45	2.02E-44
BN1708_017256	-3.09	3.84E-16	2.59E-15
BN1708_017343	-2.66	8.73E-08	3.46E-07
BN1708_017380	-2.80	1.00E-07	3.95E-07
BN1708_017458	-2.08	1.57E-48	2.83E-47
BN1708_017483	-2.06	1.81E-06	6.32E-06
BN1708_017520	-2.43	1.29E-06	4.57E-06
BN1708_017543	-2.35	5.47E-07	2.01E-06
BN1708_017547	-2.75	7.57E-173	8.30E-171
BN1708_017549	-2.29	7.00E-14	4.23E-13
BN1708_017583	-2.25	8.50E-06	2.77E-05
BN1708_017587	-2.41	1.21E-92	4.63E-91
BN1708_017611	-2.44	2.83E-46	4.88E-45
BN1708_017625	-2.21	2.73E-09	1.23E-08
BN1708_017670	-2.79	2.30E-08	9.59E-08
BN1708_017687	-2.93	1.29E-34	1.70E-33
BN1708_017749	-2.71	1.89E-08	7.94E-08
BN1708_017803	-2.74	1.18E-12	6.66E-12
BN1708_017868	-2.10	1.14E-12	6.43E-12
BN1708_017930	-2.72	1.17E-05	3.76E-05
BN1708_017977	-4.40	5.85E-10	2.77E-09
BN1708_018001	-2.36	2.77E-05	8.49E-05
BN1708_018002	-2.98	2.74E-10	1.33E-09
BN1708_018011	-5.22	3.19E-10	1.54E-09
BN1708_018016	-2.35	1.91E-13	1.13E-12
BN1708_018066	-3.18	2.32E-08	9.68E-08
BN1708_018093	-2.66	8.52E-12	4.58E-11

**Table S3: List of *Verticillium* genes most down-regulated after 120 min of co-cultivation with *Pseudomonas protegens* P_DAPG, continued.**			
**Identifier**	**Log_2-fold-change**	**P-value**	**Adjusted p-value**
BN1708_018097	-2.68	2.29E-09	1.04E-08
BN1708_018104	-3.51	7.49E-07	2.73E-06
BN1708_018122	-4.14	3.40E-11	1.76E-10
BN1708_018128	-2.80	1.83E-16	1.25E-15
BN1708_018160	-2.46	9.33E-06	3.02E-05
BN1708_018174	-2.33	2.09E-06	7.24E-06
BN1708_018233	-2.01	6.09E-05	0.000179104
BN1708_018385	-2.75	7.82E-55	1.58E-53
BN1708_018408	-2.16	3.31E-54	6.60E-53
BN1708_018478	-2.11	4.81E-10	2.29E-09
BN1708_018503	-2.14	4.22E-12	2.31E-11
BN1708_018519	-2.11	4.76E-05	0.000141903
BN1708_018525	-4.88	1.01E-113	5.14E-112
BN1708_018548	-2.01	4.00E-08	1.63E-07
BN1708_018642	-2.46	7.70E-08	3.07E-07
BN1708_018659	-2.88	9.24E-06	2.99E-05
BN1708_018675	-2.56	3.36E-64	7.97E-63
BN1708_018683	-2.45	2.20E-09	9.97E-09
BN1708_018699	-2.52	2.60E-06	8.92E-06
BN1708_018713	-2.35	5.39E-11	2.76E-10
BN1708_018784	-2.09	2.77E-08	1.15E-07
BN1708_018793	-2.13	8.44E-41	1.29E-39
BN1708_018827	-2.61	5.48E-08	2.22E-07
BN1708_018842	-3.02	6.39E-05	0.000187464
BN1708_018848	-2.50	2.64E-05	8.10E-05
BN1708_018873	-3.50	3.48E-05	0.000105497
BN1708_018897	-2.36	2.13E-10	1.04E-09
BN1708_018928	-2.50	7.95E-07	2.89E-06
BN1708_018972	-2.28	1.13E-05	3.61E-05
BN1708_019037	-2.33	2.04E-08	8.55E-08
BN1708_019139	-2.30	1.41E-06	4.98E-06
BN1708_019185	-3.46	2.88E-36	3.96E-35
BN1708_019191	-2.46	1.58E-05	4.99E-05
BN1708_019202	-2.50	2.08E-05	6.48E-05
BN1708_019228	-2.29	7.08E-12	3.82E-11
BN1708_019272	-2.04	6.03E-07	2.21E-06
BN1708_019311	-2.35	1.35E-05	4.31E-05
BN1708_019321	-2.62	2.77E-05	8.48E-05
BN1708_019338	-3.14	5.69E-10	2.69E-09
BN1708_019347	-2.25	6.16E-22	5.46E-21
BN1708_019405	-4.82	1.23E-06	4.37E-06

Table S3: List of *Verticillium* genes most down-regulated after 120 min of co-cultivation with *Pseudomonas protegens* P_DAPG, continued.

Identifier	Log_2-fold-change	P-value	Adjusted p-value
BN1708_019474	-2.26	4.63E-10	2.21E-09
BN1708_019484	-3.14	7.68E-11	3.88E-10
BN1708_019542	-2.10	1.28E-07	5.01E-07
BN1708_019694	-2.57	3.18E-05	9.68E-05
BN1708_019697	-2.22	4.33E-15	2.78E-14
BN1708_019802	-2.08	1.51E-11	8.00E-11
BN1708_019861	-2.25	1.76E-06	6.15E-06
BN1708_019868	-3.98	8.45E-20	6.79E-19
BN1708_019985	-2.15	4.21E-13	2.45E-12
BN1708_020003	-2.32	8.20E-08	3.26E-07
BN1708_020144	-4.01	9.63E-14	5.79E-13
BN1708_020292	-5.00	2.60E-11	1.36E-10
BN1708_020297	-2.71	2.37E-06	8.19E-06
BN1708_020327	-2.23	3.11E-10	1.50E-09
BN1708_020424	-2.64	4.52E-18	3.34E-17
BN1708_020489	-3.83	8.99E-21	7.53E-20
BN1708_020651	-2.99	2.62E-05	8.03E-05

Table S4: Significantly enriched GO terms of *Verticillium* transcripts most up-regulated after 120 min of co-cultivation with *Pseudomonas protegens* P_DAPG. Reads of *V. longisporum* Vl43 have been mapped to the *V. longisporum* VL1 genome. GO enrichment analysis was performed for most up-regulated genes with Log_2-fold-change > 2 and p-value < 0.0001. Identifiers of each gene are shown as well as associated GO terms and predicted domains of the deduced protein sequences.

Identifier	GO terms		Predicted domains
	Biological process	Molecular function	
BN1708_000105	GO:0046501<br>GO:0006782<br>GO:0006783<br>GO:0006779<br>GO:0006778<br>GO:0033014<br>GO:0033013		Tetrapyrrole biosynthesis, uroporphyrinogen III synthase (PF0260)
BN1708_000106		GO:0016491	ERV/ALR sulfhydryl oxidase domain (PF04777)
BN1708_000169		GO:0016491	NADH:flavin oxidoreductase/NADH oxidase, N-terminal (PF00724)
BN1708_000187		GO:0016491	Short-chain dehydrogenase/reductase SDR (PF00106)
BN1708_000251		GO:0016491	Oxoglutarate/iron-dependent dioxygenase (PF03171)
BN1708_000517		GO:0016491	ATP11 (PF06644)
BN1708_000520		GO:0016491	Haem peroxidase (PF00141)
BN1708_000782		GO:0016491	Alternative oxidase (PF01786)
BN1708_000899		GO:0016491	Catalase core domain (PF00199), Catalase immune-responsive domain (PF06628)
BN1708_000956	GO:0006560		Aminotransferase class-III (PF00202)
BN1708_000972	GO:0006560		Aminotransferase class-III (PF00202)
BN1708_001040		GO:0016491	Alcohol dehydrogenase, N-terminal (PF08240)
BN1708_001164		GO:0016491	Cytochrome P450 (PF00067)
BN1708_001360		GO:0016491	BN1708_009105
BN1708_001526		GO:0016491	Manganese/iron superoxide dismutase, C-terminal (PF02777), Manganese/iron superoxide dismutase, N-terminal (PF00081), Protein of unknown function DUF1993 (PF09351)
BN1708_001554		GO:0016491	Alcohol dehydrogenase, C-terminal (PF00107), Oxidoreductase, N-terminal domain (PF16884)

Table S4: Significantly enriched GO terms of *Verticillium* transcripts most up-regulated after 120 min of co-cultivation with *Pseudomonas protegens* P_DAPG, continued.

Identifier	GO terms		Predicted domains
	Biological process	Molecular function	
BN1708_001566		GO:0016491	D-isomer specific 2-hydroxyacid dehydrogenase, NAD-binding domain (PF02826), D-isomer specific 2-hydroxyacid dehydrogenase, catalytic domain (PF00389), Flavin monooxygenase-like (PF00743), Major facilitator superfamily (PF07690)
BN1708_001645		GO:0016491	Enoyl-(Acyl carrier protein) reductase (PF13561)
BN1708_001674		GO:0016491	Enoyl-(Acyl carrier protein) reductase (PF13561)
BN1708_001686		GO:0016491	Non-haem dioxygenase N-terminal domain (PF14226), Oxoglutarate/iron-dependent dioxygenase (PF03171)
BN1708_001741		GO:0016491	Cytochrome P450 (PF00067)
BN1708_001831		GO:0016491	DSBA-like thioredoxin domain (PF01323)
BN1708_001881		GO:0016491	Alcohol dehydrogenase, C-terminal (PF00107), Alcohol dehydrogenase, N-terminal (PF08240)
BN1708_001941		GO:0016491	Alcohol dehydrogenase, C-terminal (PF00107), Alcohol dehydrogenase, N-terminal (PF08240)
BN1708_002036		GO:0016491	Hydroxylase/desaturase AsaB-like (PTHR34598)
BN1708_002150		GO:0019825<br>GO:0016491	Globin (PF00042), Oxidoreductase FAD/NAD(P)-binding (PF00175)
BN1708_002180		GO:0019825<br>GO:0016491	Flavoprotein pyridine nucleotide cytochrome reductase-like, FAD-binding domain (PF00970), Globin (PF00042), Oxidoreductase FAD/NAD(P)-binding (PF00175)
BN1708_002181		GO:0016491	Amine oxidase (PF01593)
BN1708_002198		GO:0016491	Non-haem dioxygenase N-terminal domain (PF14226), Oxoglutarate/iron-dependent dioxygenase (PF03171)
BN1708_002249		GO:0016491	Amino acid/polyamine transporter I (PF13520), Cytochrome P450 (PF00067)
BN1708_002621		GO:0016491	Cytochrome b5-like heme/steroid binding domain (PF00173), Fatty acid hydroxylase (PF04116)
BN1708_002858		GO:0016491	TauD/TfdA-like domain (PF02668)
BN1708_003004		GO:0016491	Cysteine dioxygenase type I (PF05995)

**Table S4: Significantly enriched GO terms of *Verticillium* transcripts most up-regulated after 120 min of co-cultivation with *Pseudomonas protegens* P_DAPG, continued.**			
**Identifier**	**GO terms**		**Predicted domains**
	**Biological process**	**Molecular function**	
BN1708_003075	GO:0006081<br>GO:0046184<br>GO:0042819<br>GO:0042816<br>GO:0042822<br>GO:0042823	GO:0016491	Pyridoxamine 5'-phosphate oxidase, putative (PF01243), Pyridoxine 5'-phosphate oxidase, dimerisation, C-terminal (PF10590)
BN1708_003122		GO:0016491	D-isomer specific 2-hydroxyacid dehydrogenase, NAD-binding domain (PF02826), D-isomer specific 2-hydroxyacid dehydrogenase, catalytic domain (PF00389)
BN1708_003217		GO:0016491	Histidinol dehydrogenase (PF00815)
BN1708_003267	GO:0006783<br>GO:0006779<br>GO:0006778<br>GO:0033014<br>GO:0033013	GO:0016491	COX15/CtaA family (PF02628)
BN1708_003628		GO:0016491	Tyrosinase copper-binding domain (PF00264)
BN1708_003893		GO:0016491	Lactate/malate dehydrogenase, C-terminal (PF02866), Lactate/malate dehydrogenase, N-terminal (PF00056)
BN1708_003927		GO:0016491	FMN-dependent dehydrogenase (PF01070), FMN-dependent dehydrogenase (PF01070)
BN1708_004067		GO:0016491	Acyl-CoA dehydrogenase/oxidase C-terminal (PF00441), Acyl-CoA dehydrogenase/oxidase, N-terminal (PF02771), Acyl-CoA oxidase/dehydrogenase, central domain (PF02770), FAD-binding 8 (PF08022), Ferric reductase transmembrane component-like domain (PF01794), Ferric reductase, NAD binding domain (PF08030), Integral membrane protein SYS1-related (PF09801), Ubiquitin-conjugating enzyme E2 (PF00179)
BN1708_004406		GO:0016491	Nitroreductase (PF00881)
BN1708_004408		GO:0016491	Oxidoreductase FAD/NAD(P)-binding (PF00175), Sulfite reductase [NADPH] flavoprotein alpha-component-like, FAD-binding (PF00667)
BN1708_004546		GO:0016491	Amino acid permease/ SLC12A domain (PF00324), FAD linked oxidase, N-terminal (PF01565), NAD-dependent epimerase/dehydratase (PF01370), Short-chain dehydrogenase/reductase SDR (PF00106)

Table S4: Significantly enriched GO terms of *Verticillium* transcripts most up-regulated after 120 min of co-cultivation with *Pseudomonas protegens* P_DAPG, continued.

Identifier	GO terms		Predicted domains
	Biological process	Molecular function	
BN1708_004660	GO:0046501<br>GO:0006782<br>GO:0006783<br>GO:0006779<br>GO:0006778<br>GO:0033014<br>GO:0033013		Delta-aminolevulinic acid dehydratase (PF00490)
BN1708_004726		GO:0016491	NADH:flavin oxidoreductase/NADH oxidase, N-terminal (PF00724)
BN1708_004846		GO:0016491	Extradiol ring-cleavage dioxygenase, class III enzyme, subunit B (PF02900)
BN1708_004867	GO:0046501<br>GO:0006782<br>GO:0006783<br>GO:0006779<br>GO:0006778<br>GO:0033014<br>GO:0033013		Uroporphyrinogen decarboxylase (URO-D) (PF1208)
BN1708_005004		GO:0016491	FAD dependent oxidoreductase (PF01266)
BN1708_005008		GO:0016491	FAD dependent oxidoreductase (PF01266)
BN1708_005042	GO:0006081<br>GO:0046184<br>GO:0042819<br>GO:0042816<br>GO:0042822<br>GO:0042823	GO:0016491	Pyridoxamine 5'-phosphate oxidase, putative (PF01243), Pyridoxine 5'-phosphate oxidase, dimerisation, C-terminal (PF10590)
BN1708_005066		GO:0016491	Short-chain dehydrogenase/reductase SDR (PF00106)
BN1708_005183		GO:0016491	BP28, C-terminal domain (PF08146), FAD/NAD(P)-binding domain (PF07992)
BN1708_005304		GO:0016491	Fatty acid hydroxylase (PF04116), Inositol phosphatase (PF12456), SAC domain (PF02383)
BN1708_005327		GO:0016491	Nitroreductase (PF00881)
BN1708_005334		GO:0016491	Flavin monooxygenase-like (PF00743)

**Table S4: Significantly enriched GO terms of *Verticillium* transcripts most up-regulated after 120 min of co-cultivation with *Pseudomonas protegens* P_DAPG, continued.**			
**Identifier**	**GO terms**		**Predicted domains**
	**Biological process**	**Molecular function**	
BN1708_005555		GO:0016491	Cellulose-binding domain, fungal (PF00734), Cutinase/acetylxylan esterase (PF01083), TauD/TfdA-like domain (PF02668)
BN1708_005564		GO:0016491	Short-chain dehydrogenase/reductase SDR (PF00106)
BN1708_005595		GO:0016491	CHCH (PF06747)
BN1708_005817	GO:0046501<br>GO:0006782<br>GO:0006783<br>GO:0006779<br>GO:0006778<br>GO:0033014<br>GO:0033013	GO:0016491	Coproporphyrinogen III oxidase, aerobic (PF01218)
BN1708_005936		GO:0016491	Malic enzyme, N-terminal domain (PF00390), Malic enzyme, NAD-binding (PF03949)
BN1708_006002		GO:0016491	Alcohol dehydrogenase GroES-associated (PF13823), Alcohol dehydrogenase, C-terminal (PF00107), Alcohol dehydrogenase, N-terminal (PF08240)
BN1708_006132	GO:0006081<br>GO:0046184<br>GO:0042819<br>GO:0042816<br>GO:0042822<br>GO:0042823	GO:0016843<br>GO:0036381	PdxS/SNZ N-terminal domain (PF01680)
BN1708_006158		GO:0016491	Alcohol dehydrogenase GroES-associated (PF13823), Alcohol dehydrogenase, N-terminal (PF08240), Glycoside hydrolase, family 5 (PF00150)
BN1708_006174		GO:0016491	Arsenical pump membrane protein, ArsB (PF02040), Catechol dioxygenase, N-terminal (PF04444), Intradiol ring-cleavage dioxygenase, C-terminal (PF00775)
BN1708_006230		GO:0016491	Endoplasmic reticulum oxidoreductin 1 (PF04137)
BN1708_006324		GO:0016491	Molybdopterin dinucleotide-binding domain (PF01568), Molybdopterin oxidoreductase (PF00384), Molybdopterin oxidoreductase, 4Fe-4S domain (PF04879)
BN1708_006526	GO:0006081		Putative esterase (PF00756)
BN1708_006593		GO:0016491	D-isomer specific 2-hydroxyacid dehydrogenase, NAD-binding domain (PF02826), D-isomer specific 2-hydroxyacid dehydrogenase, catalytic domain (PF00389)

**Table S4: Significantly enriched GO terms of *Verticillium* transcripts most up-regulated after 120 min of co-cultivation with *Pseudomonas protegens* P_DAPG, continued.**			
**Identifier**	**GO terms**		**Predicted domains**
	**Biological process**	**Molecular function**	
BN1708_006703		GO:0016491	Thioredoxin-like superfamily (SSF52833)
BN1708_006838		GO:0016491	Enoyl-(Acyl carrier protein) reductase (PF13561)
BN1708_006944		GO:0016491	Multicopper oxidase, type 1 (PF00394), Multicopper oxidase, type 2 (PF07731), Multicopper oxidase, type 3 (PF07732)
BN1708_007239		GO:0016491	FAD-dependent oxidoreductase 2, FAD binding domain (PF00890)
BN1708_007262		GO:0016491	Cytochrome P450 (PF00067)
BN1708_007271		GO:0016491	Enolase C-terminal domain-like (PF13378), Short-chain dehydrogenase/reductase SDR (PF00106)
BN1708_007602		GO:0016491	Short-chain dehydrogenase/reductase SDR (PF00106)
BN1708_007675		GO:0019825<br>GO:0016491	Kelch motif (PF13854), Globin (PF00042), Oxidoreductase FAD/NAD(P)-binding (PF00175)
BN1708_007771		GO:0016491	Fatty acid hydroxylase (PF04116)
BN1708_007812		GO:0016491	Alcohol dehydrogenase, N-terminal (PF08240)
BN1708_007850		GO:0016491	Cytochrome b5-like heme/steroid binding domain (PF00173), Moybdenum cofactor oxidoreductase, dimerisation (PF03404), Oxidoreductase, molybdopterin-binding domain (PF00174)
BN1708_007889		GO:0016491	Carotenoid oxygenase (PF03055), Cytochrome P450 (PF00067)
BN1708_007900		GO:0016491	Alcohol dehydrogenase, C-terminal (PF00107)
BN1708_007904		GO:0016491	Cytochrome P450 (PF00067), Protein kinase domain (PF00069), YTH domain (PF04146)
BN1708_007923		GO:0016491	Enoyl-(Acyl carrier protein) reductase (PF13561)
BN1708_008175		GO:0016491	Mitochondrial intermembrane space import and assembly protein 40 (PTHR21622)
BN1708_008301		GO:0016491	Catechol dioxygenase, N-terminal (PF04444), Intradiol ring-cleavage dioxygenase, C-terminal (PF00775), SUI1 domain (PF01253)
BN1708_008618		GO:0016491	tRNA-dihydrouridine synthase (PF01207)
BN1708_008781		GO:0016491	Alcohol dehydrogenase, C-terminal (PF00107), Alcohol dehydrogenase, N-terminal (PF08240), Zinc finger C2H2-type (PF00096), Zinc finger C2H2-type (PF00096)
BN1708_008844		GO:0016491	Short-chain dehydrogenase/reductase SDR (PF00106)
BN1708_008877		GO:0016491	Flavoprotein pyridine nucleotide cytochrome reductase-like, FAD-binding domain (PF00970), Oxidoreductase FAD/NAD(P)-binding (PF00175)

**Table S4: Significantly enriched GO terms of *Verticillium* transcripts most up-regulated after 120 min of co-cultivation with *Pseudomonas protegens* P_DAPG, continued.**			
**Identifier**	**GO terms**		**Predicted domains**
	**Biological process**	**Molecular function**	
BN1708_008894		GO:0016491	Short-chain dehydrogenase/reductase SDR (PF00106)
BN1708_008926		GO:0016491	Cytochrome P450 (PF00067)
BN1708_008936		GO:0016491	Flavin monooxygenase-like (PF00743), GPCR, family 2, secretin-like (PF00002), Peptidase C12, ubiquitin carboxyl-terminal hydrolase (PF01088),
BN1708_009151		GO:0016491	Bacteriophage T5, Orf172 DNA-binding (PF10544), TauD/TfdA-like domain (PF02668), TauD/TfdA-like domain (PF02668)
BN1708_009172		GO:0016491	Enoyl-(Acyl carrier protein) reductase (PF13561), Serine aminopeptidase, S33 (PF12146), Transcription factor domain, fungi (PF04082), Zinc finger C2H2-type (PF00096), Zinc finger C2H2-type (PF00096), Zn(2)-C6 fungal-type DNA-binding domain (PF00172)
BN1708_009747		GO:0016491	NADH:flavin oxidoreductase/NADH oxidase, N-terminal (PF00724)
BN1708_009848		GO:0016491	Glucose-methanol-choline oxidoreductase, C-terminal (PF05199), Glucose-methanol-choline oxidoreductase, N-terminal (PF00732)
BN1708_010044		GO:0016491	Lactate/malate dehydrogenase, C-terminal (PF02866), Lactate/malate dehydrogenase, N-terminal (PF00056)
BN1708_010232		GO:0016491	Short-chain dehydrogenase/reductase SDR (PF00106)
BN1708_010397		GO:0016491	TauD/TfdA-like domain (PF02668)
BN1708_010519		GO:0016491	Fatty acid hydroxylase (PF04116)
BN1708_010632		GO:0016491	FAD/NAD(P)-binding domain (PF07992)
BN1708_010733		GO:0016491	NADP-dependent oxidoreductase domain (PF00248)
BN1708_010734		GO:0016491	NADP-dependent oxidoreductase domain (PF00248)
BN1708_010895		GO:0016491	Initiation factor 2B-related (PF01008), L-lysine 6-monooxygenase/L-ornithine 5-monooxygenase (PF13434), NUDIX hydrolase domain (PF00293)
BN1708_010900		GO:0016491	Aldehyde dehydrogenase domain (PF00171), Major facilitator, sugar transporter-like (PF00083), Major facilitator, sugar transporter-like (PF00083), Short-chain dehydrogenase/reductase SDR (PF00106), Transcription factor domain, fungi (PF04082), Zinc finger C2H2-type (PF00096), Zinc finger C2H2-type (PF00096), Zn(2)-C6 fungal-type DNA-binding domain (PF00172)
BN1708_010901		GO:0016491	Isopropylmalate dehydrogenase-like domain (PF00180)
BN1708_011003		GO:0016491	Short-chain dehydrogenase/reductase SDR (PF00106), Short-chain dehydrogenase/reductase SDR (PF00106)

Table S4: Significantly enriched GO terms of *Verticillium* transcripts most up-regulated after 120 min of co-cultivation with *Pseudomonas protegens* P_DAPG, continued.

Identifier	GO terms		Predicted domains
	Biological process	Molecular function	
BN1708_011123		GO:0016491	Haem peroxidase (PF00141)
BN1708_011133		GO:0016491	Uricase (PF01014), Uricase (PF01014)
BN1708_011151		GO:0016491	Multicopper oxidase, type 1 (PF00394), Multicopper oxidase, type 2 (PF07731), Multicopper oxidase, type 3 (PF07732)
BN1708_011265		GO:0016491	Flavoprotein pyridine nucleotide cytochrome reductase-like, FAD-binding domain (PF00970), Oxidoreductase FAD/NAD(P)-binding (PF00175)
BN1708_011307		GO:0016491	BFD-like [2Fe-2S]-binding domain (PF04324), FAD/NAD(P)-binding domain (PF07992), Nitrite/Sulfite reductase ferredoxin-like domain (PF03460), Nitrite/sulphite reductase 4Fe-4S domain (PF01077), Rieske [2Fe-2S] iron-sulphur domain (PF00355)
BN1708_011387		GO:0016491	GroES-like superfamily (SSF50129), NAD(P)-binding domain superfamily (SSF51735)
BN1708_011583		GO:0016491	Short-chain dehydrogenase/reductase SDR (PF00106)
BN1708_011752		GO:0016491	Acyl-CoA dehydrogenase, C-terminal domain (PF08028), Acyl-CoA dehydrogenase/oxidase, N-terminal (PF02771)
BN1708_011814		GO:0016491	DSBA-like thioredoxin domain (PF01323), DSBA-like thioredoxin domain (PF01323)
BN1708_011849		GO:0016491	Questin oxidase-like (PF14027)
BN1708_011882	GO:0006560	GO:0004657<br>GO:0016491	Proline dehydrogenase domain (PF01619)
BN1708_011930		GO:0016491	Glutaredoxin (PF00462)
BN1708_012007		GO:0016491	Glucose-methanol-choline oxidoreductase, C-terminal (PF05199), Glucose-methanol-choline oxidoreductase, N-terminal (PF00732)
BN1708_012066		GO:0016491	Tyrosinase copper-binding domain (PF00264)
BN1708_012079		GO:0016491	FAD linked oxidase, N-terminal (PF01565)
BN1708_012195		GO:0019825	Protoglobin (PF11563)
BN1708_012270	GO:0006560	GO:0004657<br>GO:0016491	Proline dehydrogenase domain (PF01619)
BN1708_012275		GO:0016491	Non-haem dioxygenase N-terminal domain (PF14226), Oxoglutarate/iron-dependent dioxygenase (PF03171)

**Table S4: Significantly enriched GO terms of *Verticillium* transcripts most up-regulated after 120 min of co-cultivation with *Pseudomonas protegens* P_DAPG, continued.**			
**Identifier**	**GO terms**		**Predicted domains**
	**Biological process**	**Molecular function**	
BN1708_012286		GO:0016491	Ferric reductase transmembrane component-like domain (PF01794)
BN1708_012470		GO:0016491	Enoyl-(Acyl carrier protein) reductase (PF13561)
BN1708_012475		GO:0016491	Initiation factor 2B-related (PF01008), L-lysine 6-monooxygenase/L-ornithine 5-monooxygenase (PF13434), NUDIX hydrolase domain (PF00293)
BN1708_012660		GO:0016491	Cytochrome P450 (PF00067)
BN1708_012669		GO:0016491	D-isomer specific 2-hydroxyacid dehydrogenase, NAD-binding domain (PF02826), Transcription factor Opi1 (PF08618), Transcription factor Opi1 (PF08618)
BN1708_012763	GO:0006560	GO:0004657<br>GO:0016491	Proline dehydrogenase domain (PF01619)
BN1708_012776		GO:0016491	BFD-like [2Fe-2S]-binding domain (PF04324), FAD/NAD(P)-binding domain (PF07992), Nitrite/Sulfite reductase ferredoxin-like domain (PF03460), Nitrite/sulphite reductase 4Fe-4S domain (PF01077), Rieske [2Fe-2S] iron-sulphur domain (PF00355)
BN1708_012785		GO:0016491	DSBA-like thioredoxin domain (PF01323), DSBA-like thioredoxin domain (PF01323)
BN1708_012808	GO:0006783<br>GO:0006779<br>GO:0006778<br>GO:0033014<br>GO:0033013	GO:0016491	COX15/CtaA family (PF02628)
BN1708_013191	GO:0006560	GO:0004657<br>GO:0016491	Proline dehydrogenase domain (PF01619)
BN1708_013441	GO:0006081<br>GO:0046184<br>GO:0042819<br>GO:0042816<br>GO:0042822<br>GO:0042823		Pyridoxal 5'-phosphate synthase subunit PdxT/SNO (PF01174)

Table S4: Significantly enriched GO terms of *Verticillium* transcripts most up-regulated after 120 min of co-cultivation with *Pseudomonas protegens* P_DAPG, continued.

Identifier	GO terms		Predicted domains
	Biological process	Molecular function	
BN1708_013442	GO:0006081<br>GO:0046184<br>GO:0042819<br>GO:0042816<br>GO:0042822<br>GO:0042823	GO:0016843<br>GO:0036381	PdxS/SNZ N-terminal domain (PF01680)
BN1708_013797		GO:0016491	Tyrosinase copper-binding domain (PF00264)
BN1708_013877		GO:0016491	FAD dependent oxidoreductase (PF01266), Glycosyltransferase, ALG3 (PF05208)
BN1708_013881		GO:0016491	Multicopper oxidase, type 1 (PF00394), Multicopper oxidase, type 2 (PF07731), Multicopper oxidase, type 3 (PF07732)
BN1708_013888		GO:0019825	Protoglobin (PF11563)
BN1708_013954		GO:0016491	FAD-binding 8 (PF08022), Ferric reductase transmembrane component-like domain (PF01794)
BN1708_014078		GO:0016491	Alcohol dehydrogenase, C-terminal (PF00107), Alcohol dehydrogenase, N-terminal (PF08240)
BN1708_014105		GO:0016491	Extradiol ring-cleavage dioxygenase, class III enzyme, subunit B (PF02900)
BN1708_014150		GO:0016491	Endoplasmic reticulum oxidoreductin 1 (PF04137), Endoplasmic reticulum oxidoreductin 1 (PF04137)
BN1708_014167		GO:0016491	Cytochrome b5-like heme/steroid binding domain (PF00173), Moybdenum cofactor oxidoreductase, dimerisation (PF03404), Oxidoreductase, molybdopterin-binding domain (PF00174)
BN1708_014216		GO:0016491	Acyl-CoA dehydrogenase/oxidase C-terminal (PF00441), Acyl-CoA dehydrogenase/oxidase, N-terminal (PF02771), Acyl-CoA oxidase/dehydrogenase, central domain (PF02770)
BN1708_014217		GO:0016491	Cytochrome P450 (PF00067)
BN1708_014222		GO:0016491	Aldehyde dehydrogenase domain (PF00171)
BN1708_014380		GO:0016491	ERV/ALR sulfhydryl oxidase domain (PF04777), Thiamine pyrophosphate enzyme, C-terminal TPP-binding (PF02775), Thiamine pyrophosphate enzyme, N-terminal TPP-binding domain (PF02776), Thiamine pyrophosphate enzyme, central domain (PF00205)

**Table S4: Significantly enriched GO terms of *Verticillium* transcripts most up-regulated after 120 min of co-cultivation with *Pseudomonas protegens* P_DAPG, continued.**			
**Identifier**	**GO terms**		**Predicted domains**
	**Biological process**	**Molecular function**	
BN1708_014793		GO:0016491	Malic enzyme, N-terminal domain (PF00390), Malic enzyme, NAD-binding (PF03949)
BN1708_014799		GO:0016491	GroES-like superfamily (SSF50129), NAD(P)-binding domain superfamily (SSF51735)
BN1708_014840		GO:0016491	FAD-binding 8 (PF08022), Ferric reductase transmembrane component-like domain (PF01794)
BN1708_014880		GO:0016491	Hydroxylase/desaturase AsaB-like (PTHR34598)
BN1708_014888		GO:0016491	Alcohol dehydrogenase, C-terminal (PF00107)
BN1708_014936		GO:0016491	Acetate transporter GPR1/FUN34/SatP family (PF01184), FAD linked oxidase, N-terminal (PF01565)
BN1708_014937		GO:0016491	Acyl-CoA dehydrogenase, C-terminal domain (PF08028), Acyl-CoA dehydrogenase/oxidase, N-terminal (PF02771)
BN1708_015012		GO:0016491	Non-haem dioxygenase N-terminal domain (PF14226), Oxoglutarate/iron-dependent dioxygenase (PF03171)
BN1708_015040		GO:0016491	Histidinol dehydrogenase (PF00815)
BN1708_015200		GO:0016491	FMN-dependent dehydrogenase (PF01070), FMN-dependent dehydrogenase (PF01070), FMN-dependent dehydrogenase (PF01070)
BN1708_015225		GO:0016491	Tyrosinase copper-binding domain (PF00264)
BN1708_015233		GO:0016491	FAD linked oxidase, N-terminal (PF01565)
BN1708_015846		GO:0016491	Catalase core domain (PF00199)
BN1708_015971		GO:0016491	Aldehyde dehydrogenase domain (PF00171), Kynurenine formamidase/cyclase-like (PF04199)
BN1708_016056		GO:0016491	Ferric reductase transmembrane component-like domain (PF01794)
BN1708_016238	GO:0046501<br>GO:0006782<br>GO:0006783<br>GO:0006779<br>GO:0006778<br>GO:0033014<br>GO:0033013		Delta-aminolevulinic acid dehydratase (PF00490)
BN1708_016412		GO:0016491	Haem peroxidase (PF00141)

Table S4: Significantly enriched GO terms of *Verticillium* transcripts most up-regulated after 120 min of co-cultivation with *Pseudomonas protegens* P_DAPG, continued.

**Identifier**	**GO terms**		**Predicted domains**
	**Biological process**	**Molecular function**	
BN1708_016631		GO:0016491	FAD/NAD(P)-binding domain (PF07992)
BN1708_016797		GO:0016491	Glucose-methanol-choline oxidoreductase, C-terminal (PF05199)
BN1708_016862	GO:0006560		Aminotransferase class-III (PF00202)
BN1708_016887		GO:0016491	Moybdenum cofactor oxidoreductase, dimerisation (PF03404), Oxidoreductase, molybdopterin-binding domain (PF00174)
BN1708_017214		GO:0016491	D-isomer specific 2-hydroxyacid dehydrogenase, NAD-binding domain (PF02826), D-isomer specific 2-hydroxyacid dehydrogenase, catalytic domain (PF00389)
BN1708_017233		GO:0016491	Glucose-methanol-choline oxidoreductase, C-terminal (PF05199), Glucose-methanol-choline oxidoreductase, N-terminal (PF00732)
BN1708_017240		GO:0016491	Enoyl-(Acyl carrier protein) reductase (PF13561)
BN1708_017384	GO:0006783<br>GO:0006779<br>GO:0006778<br>GO:0033014<br>GO:0033013	GO:0016491	COX15/CtaA family (PF02628)
BN1708_017602	GO:0046501<br>GO:0006782<br>GO:0006783<br>GO:0006779<br>GO:0006778<br>GO:0033014<br>GO:0033013	GO:0016491	Coproporphyrinogen III oxidase, aerobic (PF01218)
BN1708_017655		GO:0016491	Uricase (PF01014), Uricase (PF01014)
BN1708_017690		GO:0016491	Alternative oxidase (PF01786)
BN1708_017981		GO:0016491	Oxoglutarate/iron-dependent dioxygenase (PF03171)
BN1708_018098	GO:0006081<br>GO:0046184<br>GO:0042819<br>GO:0042816<br>GO:0042822<br>GO:0042823	GO:0016843<br>GO:0036381	Thiazole synthase ThiG (PF05690)

**Table S4: Significantly enriched GO terms of *Verticillium* transcripts most up-regulated after 120 min of co-cultivation with *Pseudomonas protegens* P_DAPG, continued.**			
**Identifier**	**GO terms**		**Predicted domains**
	**Biological process**	**Molecular function**	
BN1708_018240		GO:0016491	Fatty acid hydroxylase (PF04116)
BN1708_018459		GO:0016491	Tyrosinase copper-binding domain (PF00264)
BN1708_018606		GO:0016491	Multicopper oxidase, type 2 (PF07731)
BN1708_018685	GO:0046501<br>GO:0006782<br>GO:0006783<br>GO:0006779<br>GO:0006778<br>GO:0033014<br>GO:0033013		Delta-aminolevulinic acid dehydratase (PF00490)
BN1708_018745	GO:0046501<br>GO:0006782<br>GO:0006783<br>GO:0006779<br>GO:0006778<br>GO:0033014<br>GO:0033013	GO:0016491	Coproporphyrinogen III oxidase, aerobic (PF01218)
BN1708_018753		GO:0016491	Aldehyde dehydrogenase domain (PF00171)
BN1708_019028		GO:0016491	Oxidoreductase FAD/NAD(P)-binding (PF00175)
BN1708_019382		GO:0016491	Nitrite/sulphite reductase 4Fe-4S domain (PF01077)
BN1708_019717	GO:0006560	GO:0004657<br>GO:0016491	Maleylacetate reductase (IPR034786)
BN1708_020384		GO:0019825<br>GO:0016491	Globin (PF00042)
BN1708_020734	GO:0006081		S-formylglutathione hydrolase (PTHR10061)

GO:0006081 - Cellular aldehyde metabolic process

GO:0046184 - Aldehyde biosynthetic process

GO:0042819 - Vitamin B6 biosynthetic process

GO:0042816 - Vitamin B6 metabolic process

GO:0042822 - Pyridoxal phosphate metabolic process

GO:0046501 - Protoporphyrinogen IX metabolic process
GO:0006782 - Protoporphyrinogen IX biosynthetic process
GO:0006783 - Heme biosynthetic process
GO:0006779 - Porphyrin-containing compound biosynthetic process
GO:0006778 - Porphyrin-containing compound metabolic process
GO:0033014 - Tetrapyrrole biosynthetic process
GO:0042823 - Pyridoxal phosphate biosynthetic process
GO:0033013 - Tetrapyrrole metabolic process
GO:0006560 - Proline metabolic process
GO:0019825 - Oxygen binding
GO:0016491 - Oxidoreductase activity
GO:0004657 - Proline dehydrogenase activity
GO:0016843 - Amine-lyase activity
GO:0036381 - Pyridoxal 5'-phosphate synthase (glutamine hydrolyzing) activity

Table S5: Significantly enriched GO terms of *Verticillium* transcripts most down-regulated after 120 min of co-cultivation with *Pseudomonas protegens* P_DAPG. Reads of *V. longisporum* Vl43 have been mapped to the *V. longisporum* VL1 genome. GO enrichment analysis was performed for most down-regulated genes with Log_2-fold-change < -2 and p-value < 0.0001. Identifiers of each gene are shown as well as associated GO terms and predicted domains of the deduced protein sequences.

**Identifier**	**GO terms**			**Predicted domains**
	**Biological process**	**Cellular component**	**Molecular function**	
BN1708_000101	GO:0006418<br>GO:0043039		GO:0140098<br>GO:0016875<br>GO:0004812<br>GO:0140101	Aminoacyl-tRNA synthetase, class II (G/ P/ S/T) (PF00587), Anticodon-binding (PF03129)
BN1708_000693	GO:0016043		GO:0005200	Tubulin/FtsZ, 2-layer sandwich domain (PF03953), Tubulin/FtsZ, GTPase domain (PF00091)
BN1708_000854	GO:0006491			Glucosidase 2 subunit beta-like (PF13015), Glucosidase II beta subunit, N-terminal (PF12999), Glucosidase II beta subunit, N-terminal (PF12999)
BN1708_000999			GO:0140098<br>GO:0140101	Peptidyl-tRNA hydrolase, PTH2 (PF01981)
BN1708_001073		GO:0005839		Proteasome alpha-subunit, N-terminal domain (PF10584), Proteasome, subunit alpha/beta (PF00227)
BN1708_001396	GO:0016043			RecF/RecN/SMC, N-terminal (PF02463), SMCs flexible hinge (PF06470)
BN1708_001719	GO:0045229<br>GO:0016043<br>GO:0071554<br>GO:0071555			Beta galactosidase small chain/ domain 5 (PF02929), Beta-galactosidase, domain 4 (PF16353), Glycoside hydrolase family 2, catalytic domain (PF02836), Glycoside hydrolase, family 2, immunoglobulin-like beta-sandwich (PF00703), Glycosyl hydrolases family 2, sugar binding domain (PF02837)
BN1708_001794	GO:0046365<br>GO:0005996		GO:0004619	BPG-independent PGAM, N-terminal (PF06415), Metalloenzyme (PF01676), NWD NACHT-NTPase, N-terminal (PF17100)
BN1708_002112	GO:0016192			Vesicle transport protein, Got1/SFT2-like (PF04178)
BN1708_002252	GO:0045229<br>GO:0016043<br>GO:0071554<br>GO:0071555			Pectinesterase, catalytic (PF01095)
BN1708_002253			GO:0140098	mRNA (guanine-N(7))-methyltransferase domain (PF03291), mRNA (guanine-N(7))-methyltransferase domain (PF03291)
BN1708_002267	GO:0016192			Sec1-like protein (PF00995)

Significantly enriched GO terms of *Verticillium* transcripts most down-regulated after 120 min of co-cultivation with *Pseudomonas protegens* P_DAPG, continued

Identifier	GO terms			Predicted domains
	Biological process	Cellular component	Molecular function	
BN1708_002334	GO:0016192			Calcineurin-like phosphoesterase domain, lpxH-type (PF12850)
BN1708_002346	GO:0016192			Calcineurin-like phosphoesterase domain, lpxH-type (PF12850)
BN1708_003183	GO:0045229<br>GO:0016043<br>GO:0071554<br>GO:0071555		GO:0004650	Glycoside hydrolase, family 28 (PF00295) (Pectin), Thioredoxin domain (PF00085)
BN1708_003333	GO:0016043<br>GO:0016192			Anaphase-promoting complex subunit 4, WD40 domain (PF12894), WD40 repeat (PF00400), WD40 repeat (PF00400)
BN1708_003364	GO:0016043		GO:0005200	ABC transporter-like (PF00005), ABC transporter-like (PF00005), ABC-transporter extension domain (PF12848), Tubulin/FtsZ, GTPase domain (PF00091)
BN1708_003366	GO:0016043		GO:0005200	Tubulin/FtsZ, 2-layer sandwich domain (PF03953)
BN1708_003537	GO:0016043			Rad21/Rec8-like protein, N-terminal (PF04825)
BN1708_003655			GO:0140098	RNA 3'-terminal phosphate cyclase domain (PF01137)
BN1708_003698	GO:0045229<br>GO:0016043<br>GO:0071554<br>GO:0071555		GO:0004650	Glycoside hydrolase, family 28 (PF00295) (Pectin)
BN1708_003700	GO:0045229<br>GO:0016043<br>GO:0071554<br>GO:0071555		GO:0004650	Glycoside hydrolase, family 28 (PF00295) (Pectin)
BN1708_003705	GO:0005996			Alpha-L-arabinofuranosidase, C-terminal (PF06964)
BN1708_003758	GO:0016043			Conserved oligomeric Golgi complex, subunit 2, N-terminal (PF06148)
BN1708_003787	GO:0071554			Glycosyl hydrolases family 11 (GH11) domain (PF00457) (Xylan)
BN1708_003788	GO:0006418<br>GO:0043039		GO:0140098<br>GO:0016875<br>GO:0004812<br>GO:0140101	tRNA synthetases class I, catalytic domain (PF01406)
BN1708_003793	GO:0071554			Glycosyl hydrolases family 11 (GH11) domain (PF00457) (Xylan)

**Significantly enriched GO terms of *Verticillium* transcripts most down-regulated after 120 min of co-cultivation with *Pseudomonas protegens* P_DAPG, continued**				
**Identifier**	**GO terms**			**Predicted domains**
	**Biological process**	**Cellular component**	**Molecular function**	
BN1708_003794	GO:0006418<br>GO:0043039		GO:0140098<br>GO:0016875<br>GO:0004812<br>GO:0140101	tRNA synthetases class I, catalytic domain (PF01406)
BN1708_003808	GO:0005996			Serine dehydratase beta chain (PF03315), Serine dehydratase-like, alpha subunit (PF03313)
BN1708_003866	GO:0035494<br>GO:0016043<br>GO:0016192			Aspartate decarboxylase-like domain superfamily (SSF50692), CDC48 domain 2-like superfamily (SSF54585), P-loop containing nucleoside triphosphate hydrolase (SSF52540)
BN1708_003867	GO:0035494<br>GO:0016043<br>GO:0016192			AAA ATPase, AAA+ lid domain (PF17862), ATPase, AAA-type, core (PF00004)
BN1708_004094	GO:0045229<br>GO:0016043<br>GO:0071554<br>GO:0071555			Pectinesterase, catalytic (PF01095)
BN1708_004119	GO:0016043			Glycolipid transfer protein domain (PF08718)
BN1708_004254	GO:0006418<br>GO:0043039		GO:0140098<br>GO:0016875<br>GO:0004812<br>GO:0140101	Aminoacyl-tRNA synthetase, class II (D/K/N) (PF00152)
BN1708_004438	GO:0016192			Mu homology domain (PF00928)
BN1708_004495			GO:0140098<br>GO:0140101	tRNA-guanine(15) transglycosylase-like (PF01702)
BN1708_004778	GO:0045229<br>GO:0016043<br>GO:0071554<br>GO:0071555		GO:0004650	Glycoside hydrolase, family 28 (PF00295) (Pectin)
BN1708_004937	GO:0016192			Coatomer, WD associated region (PF04053), WD40 repeat (PF00400), WD40 repeat (PF00400), WD40 repeat (PF00400), WD40 repeat (PF00400)

Significantly enriched GO terms of *Verticillium* transcripts most down-regulated after 120 min of co-cultivation with *Pseudomonas protegens* P_DAPG, continued.

Identifier	GO terms			Predicted domains
	Biological process	Cellular component	Molecular function	
BN1708_005208	GO:0006418<br>GO:0043039		GO:0140098<br>GO:0016875<br>GO:0004812<br>GO:0140101	Aminoacyl-tRNA synthetase, class II (D/K/N) (PF00152), OB-fold nucleic acid binding domain, AA-tRNA synthetase-type (PF01336)
BN1708_005217	GO:0016192			AP complex, mu/sigma subunit (PF01217), Mu homology domain (PF00928)
BN1708_005666			GO:0140098	Ribonuclease kappa (PTHR31733)
BN1708_005740	GO:0016192			Exocyst complex component Sec10-like (PF07393), F-box domain (PF12937), SNF2-related, N-terminal domain (PF00176), Zinc finger, RING-type (PF13639)
BN1708_005931	GO:0045229<br>GO:0016043<br>GO:0071554<br>GO:0071555			Pectinesterase, catalytic (PF01095)
BN1708_006106	GO:0006491			Glucosidase 2 subunit beta-like (PF13015), Glucosidase II beta subunit, N-terminal (PF12999), Glucosidase II beta subunit, N-terminal (PF12999)
BN1708_006333	GO:0016043			Glycolipid transfer protein domain (PF08718)
BN1708_006385	GO:0006418<br>GO:0043039		GO:0140098<br>GO:0016875<br>GO:0004812<br>GO:0140101	Aminoacyl-tRNA synthetase, class Ia (PF00133), Methionyl/Valyl/Leucyl/Isoleucyl-tRNA synthetase, anticodon-binding (PF08264)
BN1708_006728	GO:0016043		GO:0005200	Tubulin/FtsZ, 2-layer sandwich domain (PF03953), Tubulin/FtsZ, GTPase domain (PF00091)
BN1708_007130	GO:0046365<br>GO:0005996			NADP-dependent oxidoreductase domain (PF00248)
BN1708_007358	GO:0016043			DNA replication licensing factor Mcm2 (PF12619), MCM N-terminal domain (PF14551), MCM OB domain (PF17207), MCM domain (PF00493), MCM, AAA-lid domain (PF17855)
BN1708_007364	GO:0016192			Coatomer epsilon subunit (PF04733)
BN1708_007738			GO:0140098	mRNA (guanine-N(7))-methyltransferase domain (PF03291), mRNA (guanine-N(7))-methyltransferase domain (PF03291)

**Significantly enriched GO terms of *Verticillium* transcripts most down-regulated after 120 min of co-cultivation with *Pseudomonas protegens* P_DAPG, continued.**				
**Identifier**	**GO terms**			**Predicted domains**
	**Biological process**	**Cellular component**	**Molecular function**	
BN1708_007935	GO:0045229<br>GO:0016043<br>GO:0071554<br>GO:0071555		GO:0004650	Glycoside hydrolase, family 28 (PF00295) (Pectin)
BN1708_008029	GO:0016192			Sec1-like protein (PF00995)
BN1708_008177	GO:0016192			Snare region anchored in the vesicle membrane C-terminus (PF12352)
BN1708_008288	GO:0016192			AP180 N-terminal homology (ANTH) domain (PF07651), I/LWEQ domain (PF01608)
BN1708_009126		GO:0005839	GO:0140098	Dicer dimerisation domain (PF03368), DEAD/DEAH box helicase domain (PF00270), Helicase, C-terminal (PF00271), Proteasome alpha-subunit, N-terminal domain (PF10584), Proteasome, subunit alpha/beta (PF00227), Proteasome, subunit alpha/beta (PF00227), Ribonuclease III domain (PF00636), Ribonuclease III domain (PF00636)
BN1708_009245	GO:0006418<br>GO:0043039		GO:0140098<br>GO:0016875<br>GO:0004812<br>GO:0140101	Glutamyl/glutaminyl-tRNA synthetase, class Ib, anti-codon binding domain (PF03950), Glutamyl/glutaminyl-tRNA synthetase, class Ib, catalytic domain (PF00749)
BN1708_009557	GO:0016192			Coatomer, WD associated region (PF04053), Coatomer, alpha subunit, C-terminal (PF06957), WD40 repeat (PF00400), WD40 repeat (PF00400), WD40 repeat (PF00400), WD40 repeat (PF00400), WD40 repeat (PF00400)
BN1708_009579	GO:0016043			Rad21/Rec8-like protein, N-terminal (PF04825)
BN1708_009630	GO:0016192			Vacuolar protein sorting-associated protein 54, C-terminal (PF07928), Vacuolar protein sorting-associated protein 54, N-terminal (PF10475)
BN1708_009694	GO:0005996			Glycoside hydrolase family 38, N-terminal domain (PF01074), Glycoside hydrolase family 38, central domain (PF09261), Glycosyl hydrolase family 38, C-terminal (PF07748), Glycosyl hydrolases family 38, C-terminal beta sandwich domain (PF17677)
BN1708_009722	GO:0016043			MRG domain (PF05712), RNA binding activity-knot of a chromodomain (PF11717)

Significantly enriched GO terms of *Verticillium* transcripts most down-regulated after 120 min of co-cultivation with *Pseudomonas protegens* P_DAPG, continued.

Identifier	GO terms			Predicted domains
	Biological process	Cellular component	Molecular function	
BN1708_010166	GO:0006418<br>GO:0043039		GO:0140098<br>GO:0016875<br>GO:0004812<br>GO:0140101	Arginyl-tRNA synthetase, catalytic core domain (PF00750), DALR anticodon binding (PF05746)
BN1708_010426	GO:0006418<br>GO:0043039		GO:0140098<br>GO:0016875<br>GO:0004812<br>GO:0140101	Anticodon-binding (PF03129), Proline-tRNA ligase, class II, C-terminal (PF09180)
BN1708_010428	GO:0006418<br>GO:0043039		GO:0140098<br>GO:0016875<br>GO:0004812<br>GO:0140101	Aminoacyl-tRNA synthetase, class II (G/ P/ S/T) (PF00587), Anticodon-binding (PF03129), Proline-tRNA ligase, class II, C-terminal (PF09180)
BN1708_010762	GO:0016192			Vacuolar protein sorting-associated protein 54, C-terminal (PF07928), Vacuolar protein sorting-associated protein 54, N-terminal (PF10475)
BN1708_010940	GO:0005996			Galactose-1-phosphate uridyl transferase, C-terminal (PF02744), Nucleoporin, Nup133/Nup155-like, C-terminal (PF03177), Nucleoporin, Nup133/Nup155-like, C-terminal (PF03177), Nucleoporin, Nup133/Nup155-like, C-terminal (PF03177), Nucleoporin, Nup133/Nup155-like, N-terminal (PF08801), Nucleoporin, Nup133/Nup155-like, N-terminal (PF08801), Short-chain dehydrogenase/reductase SDR (PF00106), Short-chain dehydrogenase/reductase SDR (PF00106)
BN1708_011278		GO:0005839		Proteasome, subunit alpha/beta (PF00227)
BN1708_011447	GO:0046365<br>GO:0005996			NADP-dependent oxidoreductase domain (PF00248)
BN1708_012092	GO:0006418<br>GO:0043039		GO:0140098<br>GO:0016875<br>GO:0004812<br>GO:0140101	Aminoacyl-tRNA synthetase, class II (G/ P/ S/T) (PF00587), Aminotransferase, class I/classII (PF00155), Serine-tRNA synthetase, type1, N-terminal (PF02403)
BN1708_012492	GO:0016192			AP180 N-terminal homology (ANTH) domain (PF07651), I/LWEQ domain (PF01608)

Significantly enriched GO terms of *Verticillium* transcripts most down-regulated after 120 min of co-cultivation with *Pseudomonas protegens* P_DAPG, continued.

Identifier	GO terms			Predicted domains
	Biological process	Cellular component	Molecular function	
BN1708_012880	GO:0005996			Alpha-L-arabinofuranosidase, C-terminal (PF06964)
BN1708_013772			GO:0140098<br>GO:0140101	Peptidyl-tRNA hydrolase, PTH2 (PF01981), Peptidyl-tRNA hydrolase, PTH2 (PF01981)
BN1708_013968	GO:0005996			Serine dehydratase beta chain (PF03315), Serine dehydratase-like, alpha subunit (PF03313)
BN1708_014119			GO:0140098	Ribonuclease II/R (PF00773)
BN1708_014301	GO:0016043<br>GO:0016192			Gelsolin-like domain (PF00626), Sec23/Sec24 beta-sandwich (PF08033), Sec23/Sec24, helical domain (PF04815), Sec23/Sec24, trunk domain (PF04811), Zinc finger, Sec23/Sec24-type (PF04810)
BN1708_014370			GO:0140098	RNA 3'-terminal phosphate cyclase domain (PF01137)
BN1708_014635			GO:0140098	Ribonuclease III domain (PF00636)
BN1708_014839	GO:0006418<br>GO:0043039		GO:0140098<br>GO:0016875<br>GO:0004812<br>GO:0140101	Aminoacyl-tRNA synthetase, class II (D/K/N) (PF00152)
BN1708_015003	GO:0006418<br>GO:0043039		GO:0140098<br>GO:0016875<br>GO:0004812<br>GO:0140101	Leucyl-tRNA synthetase, class Ia, archaeal/eukaryotic cytosolic (PTHR45794)
BN1708_015082	GO:0046365<br>GO:0005996		GO:0004619	BPG-independent PGAM, N-terminal (PF06415), Metalloenzyme (PF01676)
BN1708_015118	GO:0016192			-
BN1708_015375		GO:0005839		Proteasome alpha-subunit, N-terminal domain (PF10584), Proteasome, subunit alpha/beta (PF00227), Proteasome, subunit alpha/beta (PF00227)
BN1708_015661	GO:0045229<br>GO:0016043<br>GO:0071554<br>GO:0071555		GO:0004650	Glycoside hydrolase, family 28 (PF00295) (Pectin)
BN1708_015705	GO:0016192			Vacuolar sorting protein 39/Transforming growth factor beta receptor-associated domain 2 (PF10367)
BN1708_016438		GO:0005839		Proteasome, subunit alpha/beta (PF00227)

Significantly enriched GO terms of *Verticillium* transcripts most down-regulated after 120 min of co-cultivation with *Pseudomonas protegens* P_DAPG, continued.

Identifier	GO terms			Predicted domains
	Biological process	Cellular component	Molecular function	
BN1708_016482	GO:0035494<br>GO:0016043<br>GO:0016192			AAA ATPase, AAA+ lid domain (PF17862), ATPase, AAA-type, core (PF00004), ATPase, AAA-type, core (PF00004)
BN1708_016713	GO:0016043			Lunapark domain (PF10058)
BN1708_016841	GO:0016043		GO:0005200	Tubulin/FtsZ, 2-layer sandwich domain (PF03953), Tubulin/FtsZ, GTPase domain (PF00091)
BN1708_017053	GO:0006418<br>GO:0043039		GO:0140098<br>GO:0016875<br>GO:0004812<br>GO:0140101	Nucleic acid-binding, OB-fold (SSF50249)
BN1708_017483	GO:0016192			Coatomer, WD associated region (PF04053), WD40 repeat (PF00400)
BN1708_017549	GO:0016043		GO:0005200	Tubulin/FtsZ, 2-layer sandwich domain (PF03953), Tubulin/FtsZ, GTPase domain (PF00091)
BN1708_017583	GO:0016192			Pep3/Vps18/deep orange (PF05131)
BN1708_017749	GO:0046365<br>GO:0005996		GO:0004619	Metalloenzyme (PF01676)
BN1708_018001		GO:0005839		Proteasome, subunit alpha/beta (PF00227)
BN1708_018066	GO:0006491			Glucosidase 2 subunit beta-like (PF13015)
BN1708_018097		GO:0005839		Proteasome, subunit alpha/beta (PF00227)
BN1708_018122	GO:0045229<br>GO:0016043<br>GO:0071554<br>GO:0071555		GO:0004650	Glycoside hydrolase, family 28 (PF00295) (Pectin)
BN1708_018548	GO:0016192			Mu homology domain (PF00928)
BN1708_018699	GO:0035494<br>GO:0016043<br>GO:0016192			AAA ATPase, AAA+ lid domain (PF17862), ATPase, AAA-type, core (PF00004)

Significantly enriched GO terms of *Verticillium* transcripts most down-regulated after 120 min of co-cultivation with *Pseudomonas protegens* P_DAPG, continued.

Identifier	GO terms			Predicted domains
	Biological process	Cellular component	Molecular function	
BN1708_018897	GO:0006418<br>GO:0043039		GO:0140098<br>GO:0016875<br>GO:0004812<br>GO:0140101	Aminoacyl-tRNA synthetase, class II (D/K/N) (PF00152)
BN1708_019037		GO:0005839		Proteasome, subunit alpha/beta (PF00227)
BN1708_019139	GO:0016192			Coatomer, WD associated region (PF04053)
BN1708_019338	GO:0006418<br>GO:0043039		GO:0140098<br>GO:0016875<br>GO:0004812<br>GO:0140101	Anticodon-binding (PF03129)
BN1708_019347	GO:0016043		GO:0005200	Tubulin/FtsZ, 2-layer sandwich domain (PF03953)
BN1708_020489	GO:0045229<br>GO:0016043<br>GO:0071554<br>GO:0071555		GO:0004650	Glycoside hydrolase, family 28 (PF00295) (Pectin)

GO:0006418 - tRNA aminoacylation for protein translation
GO:0046365 - monosaccharide catabolic process
GO:0071555 - cell wall organization
GO:0045229 - external encapsulating structure organization
GO:0005996 - monosaccharide metabolic process
GO:0035494 - SNARE complex disassembly
GO:0043039 - tRNA aminoacylation
GO:0016043 - cellular component organization
GO:0071554 - cell wall organization or biogenesis
GO:0016192 - vesicle-mediated transport
GO:0006491 - N-glycan processing
GO:0005839 - proteasome core complex
GO:0005200 - structural constituent of cytoskeleton

GO:0140098 - catalytic activity, acting on RNA
GO:0004650 - polygalacturonase activity
GO:0016875 - ligase activity, forming carbon-oxygen bonds
GO:0004812 - aminoacyl-tRNA ligase activity
GO:0140101 - catalytic activity, acting on a tRNA
GO:0004619 - phosphoglycerate mutase activity

Table S6: Significant differences between the inhibition of *Verticillium* and *Aspergillus* species by lipopeptide producing fluorescent pseudomonads. Statistical differences of the data shown in Figure 1 were calculated with two-tailed Student's T-test (*p < 0.05, **p < 0.01, ***p < 0.001).

	E. coli	DF41	N2C3	N2C3 Δ*LUXR*	N2C3 Δ*SYR* Δ*SYP*	N2C3 Δ*SYR*	N2C3 Δ*SYP*	WCS 365	N2E2
V. longisporum vs *V. dahliae*		*							**
V. longisporum vs *A. nidulans*		**	**				*		
V. longisporum vs *A. fumigatus*		**	*				**		
V. dahliae vs. *A. nidulans*		**	**						***
V. dahliae vs. *A. fumigatus*		**	*				**		***
A. nidulans vs *A. fumigatus*							*		

Supplementary Figures

(A)

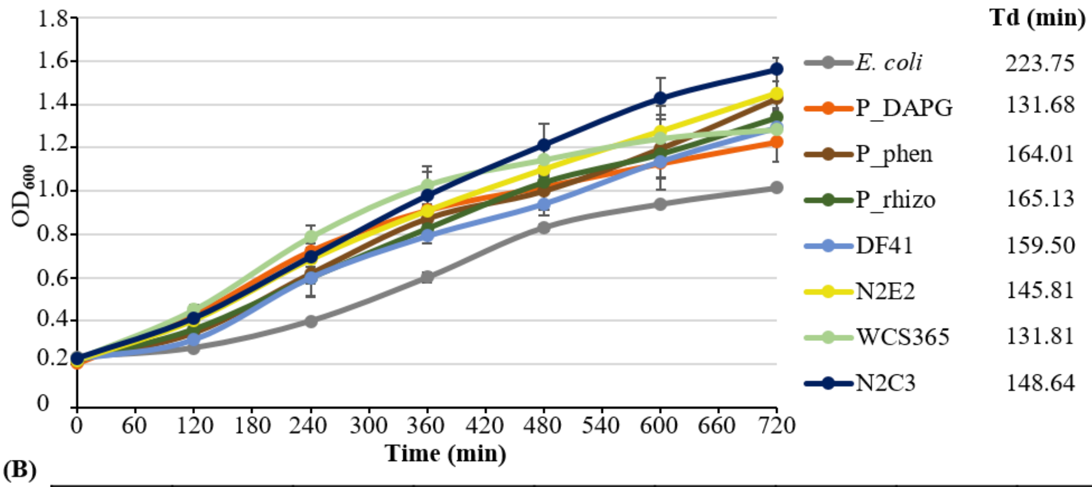

(B)

	E. coli	P_DAPG	P_phen	P_rhizo	DF41	N2E2	WCS365	N2C3
E. coli		***	***	**	**	***	***	***
P_DAPG	p=0.00004		**	*	*	ns	ns	**
P_phen	p=0.0007	p=0.00354		ns	ns	ns	**	ns
P_rhizo	p=0.0078	p=0.04179	p=0.93006		ns	ns	*	ns
DF41	p=0.00179	p=0.02496	p=0.64287	p=0.69733		ns	*	ns
N2E2	p=0.00032	p=0.06986	p=0.06294	p=0.19123	p=0.21466		ns	ns
WCS365	p=0.00008	p=0.97763	p=0.00587	p=0.04642	p=0.03087	p=0.09574		*
N2C3	p=0.00012	p=0.00931	p=0.05018	p=0.22341	p=0.25333	p=0.66211	p=0.02229	

(C)

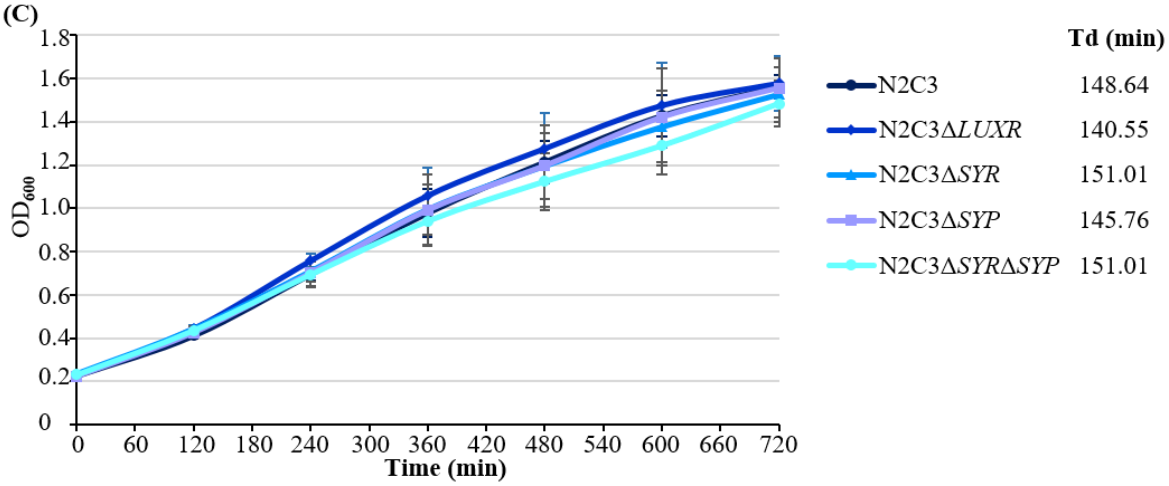

Figure S1: Doubling time between bacterial wild type strains slightly differs, but N2C3 mutant strains grow indifferent from N2C3 wild type. Bacteria were inoculated to an OD_{600} of 0.2 in liquid pectin-rich simulated xylem medium. Cultures were incubated at 25 °C and grown for 12 hours. Directly after inoculation and every two hours OD_{600} was measured. **(A)** OD_{600} is graphicaly depicted for all wild type *Pseudomonas* strains used in this study (P_DAPG, P_phen, P_rhizo, DF41, N2E2, WCS365, N2C3) and *E. coli*. Data points represent the mean of three biological replicates and error bars the respective standard deviation. The doubling time (Td) of the individual bacterial strains was determined from the exponential growth phase of each biological replicate and mean Td's are listed. **(B)** The mean Td's were compared between the bacterial wild type strains depicted in (A). Significant differences were calculated with two-tailed Student's T-test (*$p < 0.05$, **$p < 0.01$, ***$p < 0.001$). Respective p-values are listed in the left-bottom part of the table, while resulting significance levels are indicated in the top-right part. **(C)** OD_{600} of N2C3 wild type and N2C3 deletion strains Δ*LUXR* (neither syringomycin nor syringopeptin synthesis), Δ*SYR* (no syringomycin synthesis), Δ*SYP* (no syringopeptin synthesis) and Δ*SYR*Δ*SYP* (neither syringomycin nor syringopeptin synthesis) is graphically depicted. Data points represent the mean of three biological replicates and error bars the respective standard deviation. The doubling time (Td) of the individual bacterial strains with values representing the mean Td determined from the exponential growth phase of three biological replicates is listed. No significant differences between wild type and bacterial mutants were observed.

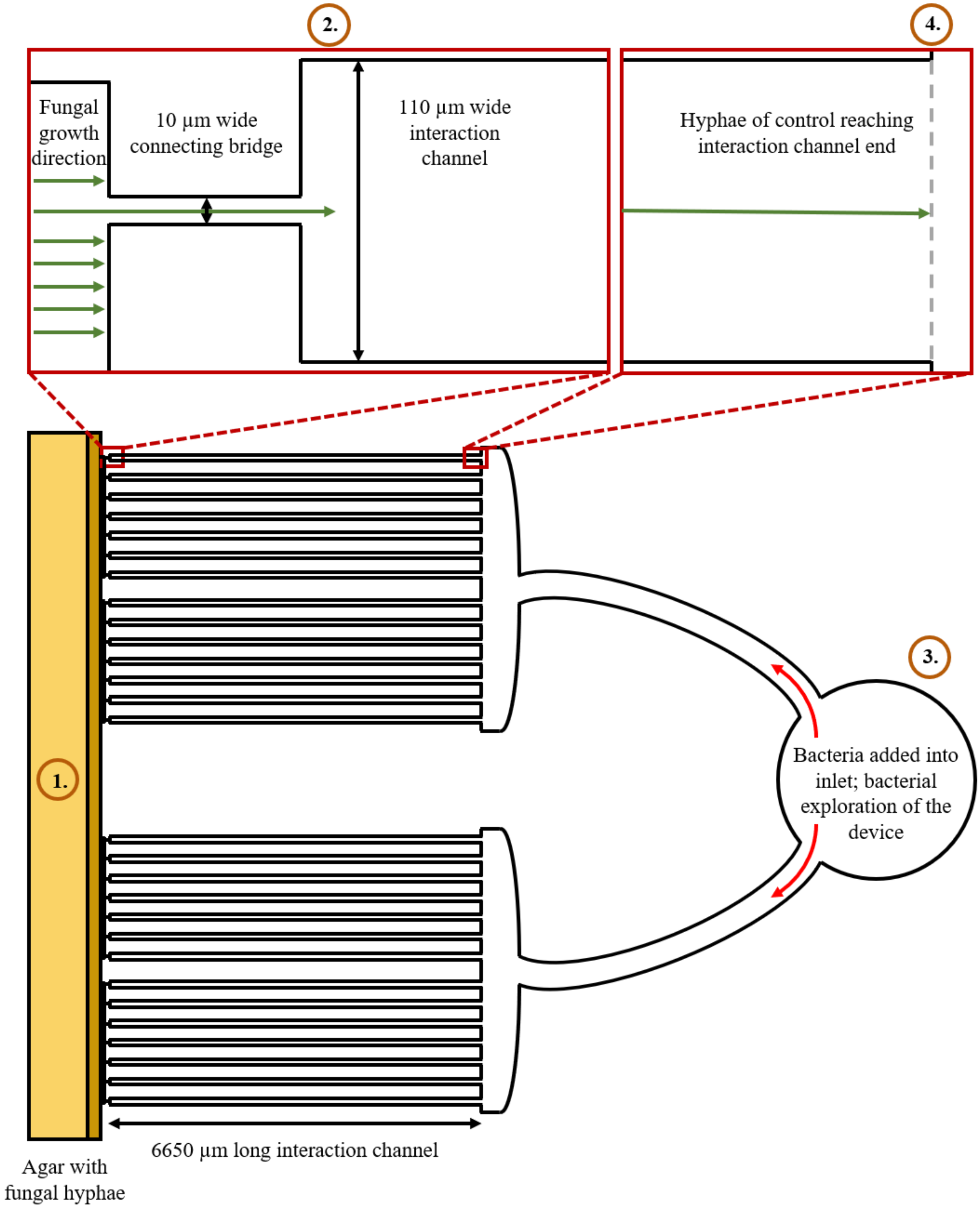
2.
4.
Fungal growth direction
10 µm wide connecting bridge
110 µm wide interaction channel
Hyphae of control reaching interaction channel end
3.
Bacteria added into inlet; bacterial exploration of the device
1.
Agar with fungal hyphae
6650 µm long interaction channel

Figure S2: Schematic representation of the microfluidic interaction device and important experimental steps. Depicted is a scheme of the microfluidic interaction device used in our experimental setup. The device was filled with liquid pectin-rich SXM. (1) As a first step a piece of agar with pre-grown fungal hyphae was pressed against the device and incubated at 25 °C. (2) Hyphae grew into the device, and only few hyphae could pass through a small connecting bridge (10 µm wide) to eventually reach the beginning of the interaction channel (110 µm wide, 6650 µm long). (3) Once the hyphae reached the interaction channels bacteria were added into the inlet (right side) and allowed to explore the device. The device was further incubated at 25 °C. (4) Fungal growth in presence of bacteria was evaluated when fungal hyphae of the untreated control reached the end of the interaction channels. Green arrows symbolize fungal growth (from left to right), red arrows indicate bacterial movement from the inlet. More detailed images of the device are published by Stanley et al. (2014).

(A)

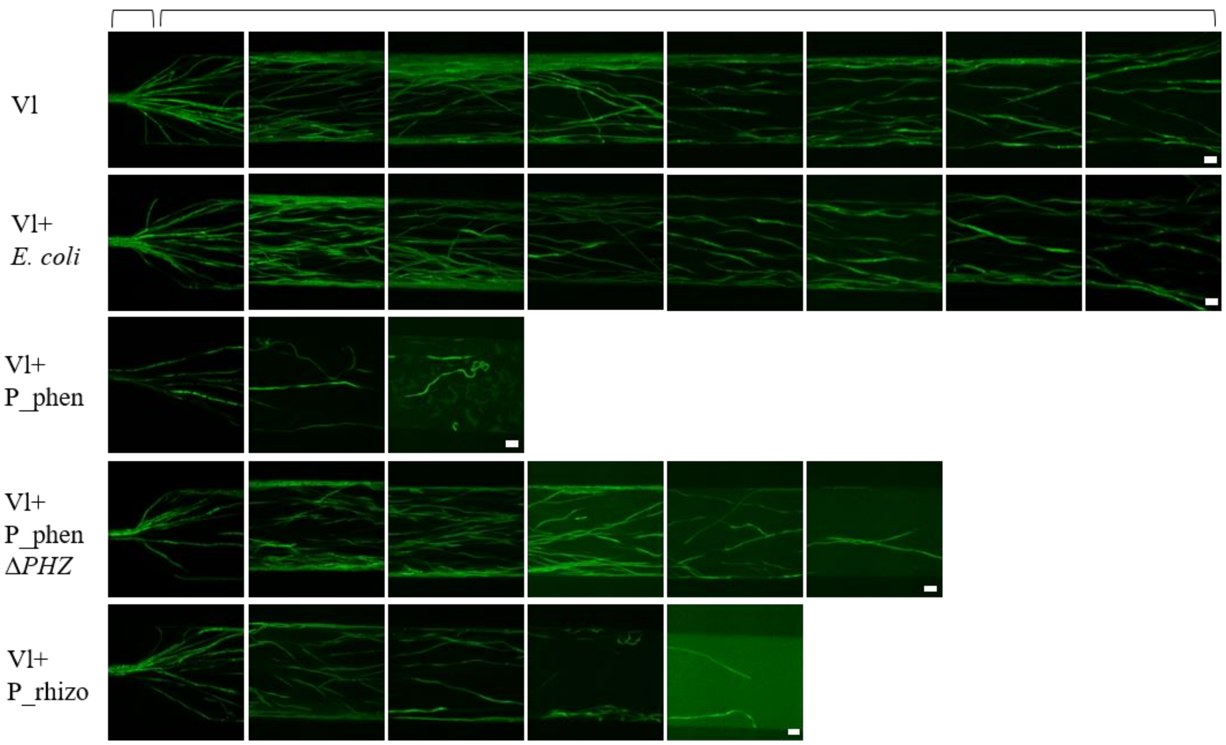
Vl
Vl + bacterium
Vl
Vl+
E. coli
Vl+
P_phen
Vl+
P_phen
ΔPHZ
Vl+
P_rhizo

(B)

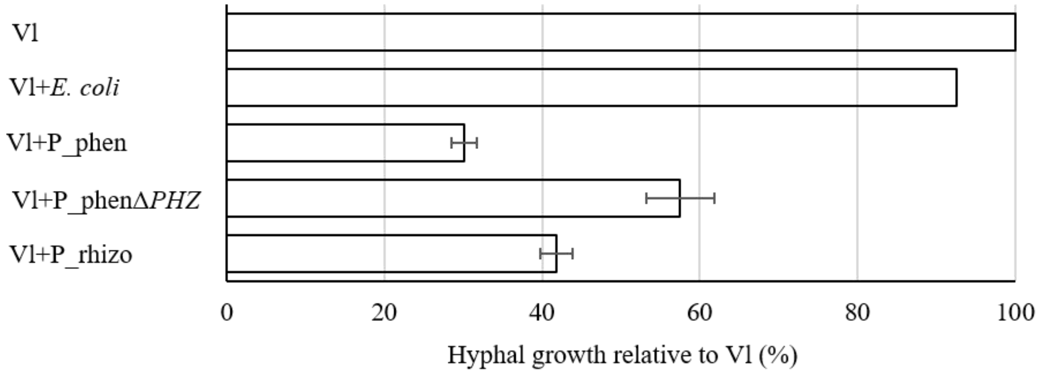
Vl
Vl+E. coli
Vl+P_phen
Vl+P_phenΔPHZ
Vl+P_rhizo
0
20
40
60
80
100
Hyphal growth relative to Vl (%)

(C)

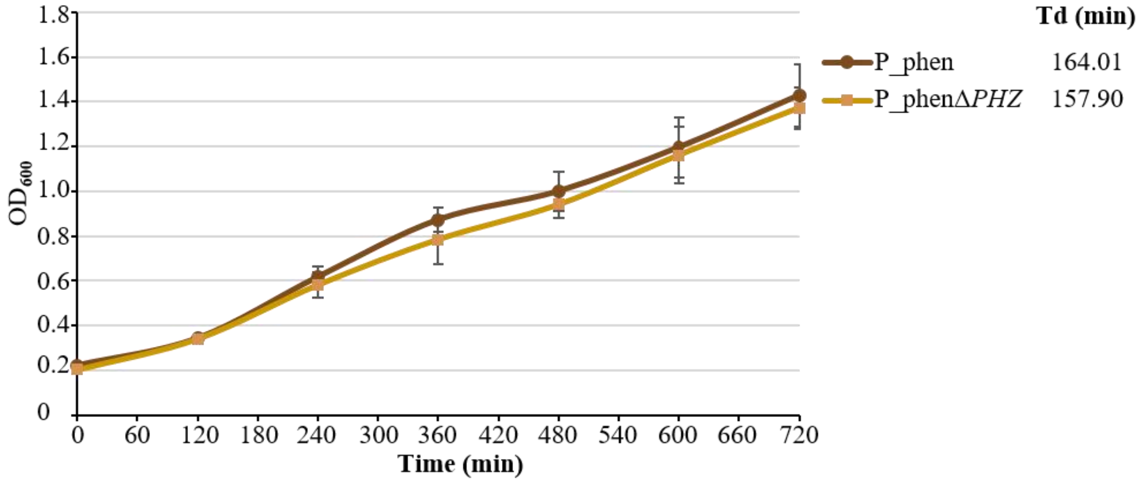
1.8
1.6
1.4
1.2
1.0
0.8
0.6
0.4
0.2
0
OD600
0
60
120
180
240
300
360
420
480
540
600
660
720
Time (min)
Td (min)
P_phen 164.01
P_phenΔPHZ 157.90

Figure S3: P_phen and P_rhizo inhibit fungal growth in microfluidic devices. Co-cultivation was performed in liquid pectin-rich simulated xylem medium. *V. longisporum* Vl43 producing high amounts of GFP (Vl) was inoculated at one site of the device with an agar block containing hyphae. The device was incubated at 25 °C until hyphae entered the microchannels. As soon as the fungal hyphae reached the beginning of the interaction channels, fluorescent pseudomonads were inoculated at the opposite end of the channel. Bacteria spread throughout the device. Three devices were evaluated for the control without bacteria, two devices for co-cultivation with *Pseudomonas* strains and one device for *E. coli* control. **(A)** Representative micrographs of a *V. longisporum* strain producing high amounts of GFP without (Vl) and with fluorescent bacteria with genes for phenazine synthesis (Vl+P_phen), a mutant strain impaired in phenazine production (P_phenΔ*PHZ*), a bacterial isolate from the rhizosphere of rapeseed (P_rhizo) or *E. coli* as control. Scale bars: 20 µm. **(B)** Graphic representation of fungal growth in microfluidic devices relative to *V. longisporum* growth without bacteria (Vl), which was set to 100% for each device. Bars represent the mean of two devices and error bars the respective standard deviation. **(C)** For doubling time comparison, P_phen wild type and mutant strain P_phenΔ*PHZ* were inoculated to an OD_{600} of 0.2 in liquid pectin-rich simulated xylem medium. Cultures were incubated at 25 °C and grown for 12 hours. Directly after inoculation and every two hours OD_{600} was measured. Data points represent the mean of three biological replicates and error bars the respective standard deviation. The doubling time (Td) of the individual bacterial strains was determined from the exponential growth phase of each biological replicate. The mean Td is listed. No significant differences in Td were observed between wild type and the bacterial mutant strains.

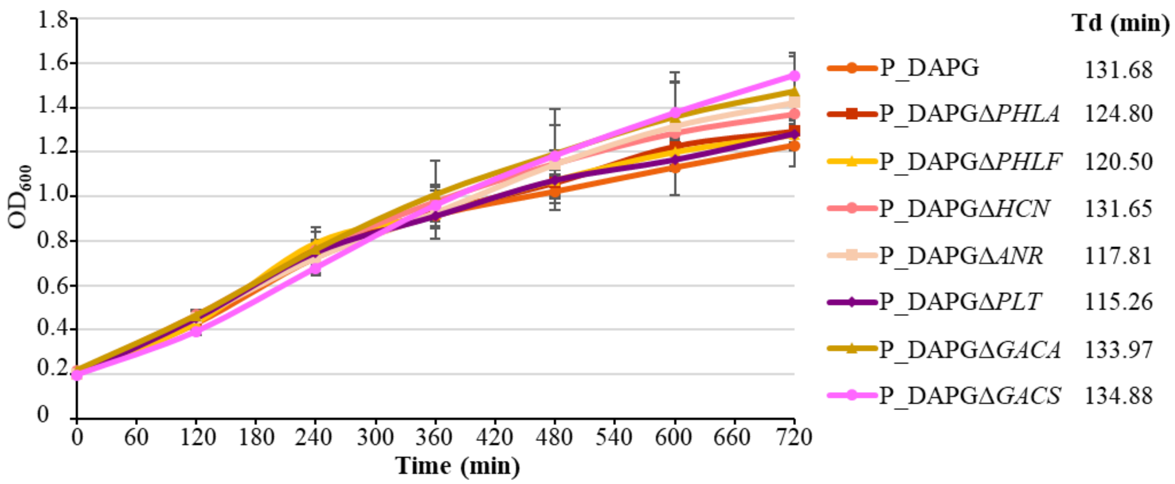

Figure S4: Doubling times of P_DAPG mutant strains are indifferent from wild type. P_DAPG wild type and mutant strains impaired in DAPG production (P_DAPGΔ*PHLA*) or overproducing DAPG (P_DAPGΔ*PHLF*), mutants deficient in HCN production (P_DAPGΔ*HCN* and P_DAPGΔ*ANR*), unable to produce pyoluteorin (P_DAPGΔ*PLT*) or mutants lacking a functional GacS/GacA two-component system (P_DAPGΔ*GACS* and P_DAPGΔ*GACA*) were inoculated to an OD_{600} of 0.2 in liquid pectin-rich simulated xylem medium. Cultures were incubated at 25 °C and grown for 12 hours. Directly after inoculation and every two hours OD_{600} was measured. Data points represent the mean of three biological replicates and error bars the respective standard deviation. The doubling time (Td) of the individual bacterial strains was determined from the exponential growth phase of each biological replicate. The individual mean Td is listed and no significant differences between wild type and bacterial mutants were observed.

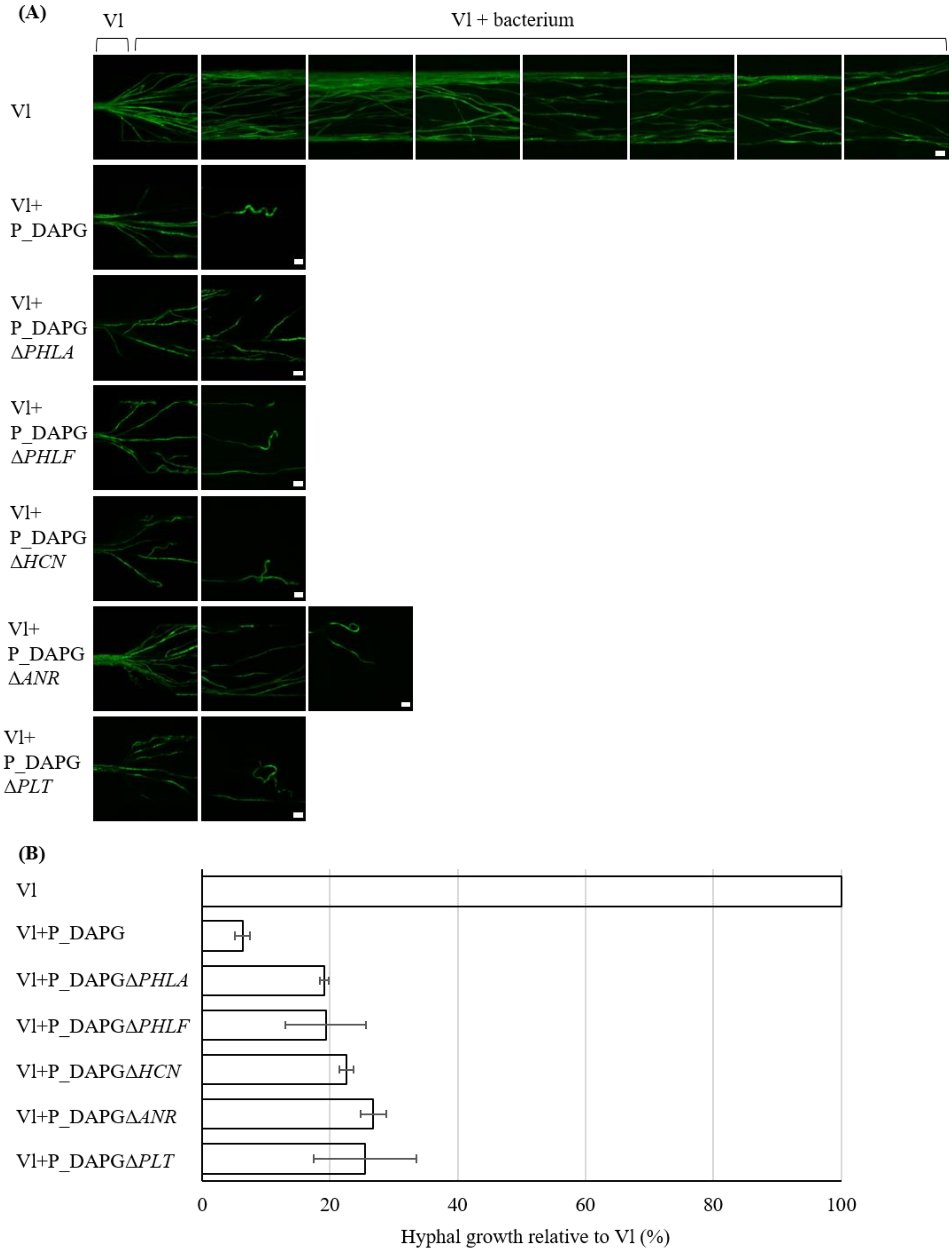

(A)
Vl
Vl + bacterium
Vl
Vl+ P_DAPG
Vl+ P_DAPG ΔPHLA
Vl+ P_DAPG ΔPHLF
Vl+ P_DAPG ΔHCN
Vl+ P_DAPG ΔANR
Vl+ P_DAPG ΔPLT
(B)
Vl
Vl+P_DAPG
Vl+P_DAPGΔPHLA
Vl+P_DAPGΔPHLF
Vl+P_DAPGΔHCN
Vl+P_DAPGΔANR
Vl+P_DAPGΔPLT
0
20
40
60
80
100
Hyphal growth relative to Vl (%)

Figure S5: The antagonistic potential of P_DAPG towards *V. longisporum* in microfluidic devices is independent from DAPG, pyoluteorin and HCN. Co-cultivation was performed in liquid pectin-rich simulated xylem medium. *V. longisporum* Vl43 producing high amounts of GFP (Vl) was inoculated at one site of the device with an agar block containing hyphae. The device was incubated at 25 °C until hyphae entered the microchannels. As soon as the fungal hyphae reached the beginning of the interaction channels, fluorescent pseudomonads were inoculated at the opposite end of the channel. Bacteria spread throughout the device. Two devices were analyzed for respective mutant strains and three devices for control without bacteria or with P_DAPG wild type. **(A)** Representative micrographs of a *V. longisporum* strain producing high amounts of GFP without (Vl) and with fluorescent bacteria with the P_DAPG wild type (Vl+P_DAPG), a mutant strain impaired in DAPG production (P_DAPGΔ*PHLA*) or overproducing DAPG (P_DAPGΔ*PHLF*), mutants deficient in HCN production (P_DAPGΔ*HCN* and P_DAPGΔ*ANR*) or unable to produce pyoluteorin (P_DAPGΔ*PLT*). Scale bars: 20 µm. **(B)** Graphic representation of fungal growth in microfluidic devices relative to *V. longisporum* growth without bacteria (Vl), which was set to 100% for each device. Bars represent the mean of two devices and error bars the respective standard deviation. Similar inhibitory potential was observed for all strains.

2 References

Berg, G., and Ballin, G. (1994). Bacterial antagonists to *Verticillium dahliae* Kleb. *J. Phytopathol.* 141, 99–110. doi:10.1111/j.1439-0434.1994.tb01449.x.

Eynck, C., Koopmann, B., Grunewaldt-Stoecker, G., Karlovsky, P., and von Tiedemann, A. (2007). Differential interactions of *Verticillium longisporum* and *V. dahliae* with *Brassica napus* detected with molecular and histological techniques. *Eur. J. Plant Pathol.* 118, 259–274. doi:10.1007/s10658-007-9144-6.

Fradin, E. F., Zhang, Z., Juarez Ayala, J. C., Castroverde, C. D. M., Nazar, R. N., Robb, J., et al. (2009). Genetic dissection of Verticillium wilt resistance mediated by tomato Ve1. *Plant Physiol.* 150, 320–332. doi:10.1104/pp.109.136762.

Geels, F. P., and Schippers, B. (1983). Selection of antagonistic fluorescent *Pseudomonas* spp. and their root colonization and persistence following treatment of seed potatoes. *J. Phytopathol.* 108, 193–206. doi:10.1111/j.1439-0434.1983.tb00579.x.

Khan, S. R., Mavrodi, D. V., Jog, G. J., Suga, H., Thomashow, L. S., and Farrand, S. K. (2005). Activation of the phz operon of *Pseudomonas fluorescens* 2-79 requires the LuxR homolog PhzR, N-(3-OH-hexanoyl)-L-homoserine lactone produced by the LuxI homolog PhzI, and a cis-acting phz box. *J. Bacteriol.* 187, 6517–6527. doi:10.1128/JB.187.18.6517-6527.2005.

Krappmann, S., Sasse, C., and Braus, G. H. (2006). Gene targeting in *Aspergillus fumigatus* by homologous recombination is facilitated in a nonhomologous end-joining-deficient genetic background. *Eukaryot. Cell* 5, 212–215. doi:10.1128/EC.5.1.212-215.2006.

Laville, J., Blumer, C., Von Schroetter, C., Gaia, V., Défago, G., Keel, C., et al. (1998). Characterization of the hcnABC gene cluster encoding hydrogen cyanide synthase and anaerobic regulation by ANR in the strictly aerobic biocontrol agent *Pseudomonas fluorescens* CHA0. *J. Bacteriol.* 180, 3187–3196. doi:10.1128/jb.180.12.3187-3196.1998.

Laville, J., Voisard, C., Keel, C., Maurhofer, M., Défago, G., and Haas, D. (1992). Global control in *Pseudomonas fluorescens* mediating antibiotic synthesis and suppression of black root rot of tobacco. *Proc. Natl. Acad. Sci. U. S. A.* 89, 1562–1566. doi:10.1073/pnas.89.5.1562.

Maurhofer, M., Keel, C., Haas, D., and Défago, G. (1994). Pyoluteorin production by *Pseudomonas fluorescens* strain CHA0 is involved in the suppression of *Pythium* damping-off of cress but not of cucumber. *Eur. J. Plant Pathol.* 100, 221–232. doi:10.1007/BF01876237.

McCluskey, K., Wiest, A., and Plamann, M. (2010). The Fungal Genetics Stock Center: a repository for 50 years of fungal genetics research. *J. Biosci.* 35, 119–126. doi:10.1007/s12038-010-0014-6.

Melnyk, R. A., Hossain, S. S., and Haney, C. H. (2019). Convergent gain and loss of genomic islands drive lifestyle changes in plant-associated *Pseudomonas*. *ISME J.* 13, 1575–1588. doi:10.1038/s41396-019-0372-5.

Pontecorvo, G., Roper, J. A., Hemmons, L. M., MacDonald, K. D., and Bufton, A. W. J. (1953). The genetics of *Aspergillus nidulans*. *Adv. Genet.* 5, 141–238. doi:10.14821/stomatopharyngology1989.9.178.

Price, M. N., Wetmore, K. M., Waters, R. J., Callaghan, M., Ray, J., Liu, H., et al. (2018). Mutant phenotypes for thousands of bacterial genes of unknown function. *Nature* 557, 503–509. doi:10.1038/s41586-018-0124-0.

Savchuk, S., and Fernando, W. G. D. (2004). Effect of timing of application and population dynamics on the degree of biological control of *Sclerotinia sclerotiorum* by bacterial antagonists. *FEMS Microbiol. Ecol.* 49, 379–388. doi:10.1016/j.femsec.2004.04.014.

Schnider-Keel, U., Seematter, A., Maurhofer, M., Blumer, C., Duffy, B., Gigot-Bonnefoy, C., et al. (2000). Autoinduction of 2,4-diacetylphloroglucinol biosynthesis in the biocontrol agent *Pseudomonas fluorescens* CHA0 and repression by the bacterial metabolites salicylate and pyoluteorin. *J. Bacteriol.* 182, 1215–1225. doi:10.1128/JB.182.5.1215-1225.2000.

Stanley, C. E., Stöckli, M., Van Swaay, D., Sabotič, J., Kallio, P. T., Künzler, M., et al. (2014). Probing bacterial-fungal interactions at the single cell level. *Integr. Biol.* 6, 935–945. doi:10.1039/c4ib00154k.

Stutz, E. W., Defago, G., and Kern, H. (1986). Naturally occuring fluorescent pseudomonads involved in suppression of black root rot of tobacco. *Phytopathology* 76, 181–185. doi:10.1094/Phyto-76-181.

Thorgersen, M. P., Lancaster, W. A., Vaccaro, B. J., Poole, F. L., Rocha, A. M., Mehlhorn, T., et al. (2015). Molybdenum availability is key to nitrate removal in contaminated groundwater environments. *Appl. Environ. Microbiol.* 81, 4976–4983. doi:10.1128/AEM.00917-15.

Tran, V.-T., Braus-Stromeyer, S. A., Kusch, H., Reusche, M., Kaever, A., Kühn, A., et al. (2014). *Verticillium* transcription activator of adhesion Vta2 suppresses microsclerotia formation and is required for systemic infection of plant roots. *New Phytol.* 202, 565–581. doi:10.1111/nph.12671.

Weller, D. M., and Cook, R. J. (1983). Suppression of take-all of wheat by seed treatments with fluorescent pseudomonads. *Phytopathology* 73, 463–469. doi:10.1094/Phyto-73-463.

Woodcock, D. M., Crowther, P. J., Doherty, J., Jefferson, S., DeCruz, E., Noyer-Weidner, M., et al. (1989). Quantitative evaluation of *Escherichia coli* host strains for tolerance to cytosine methylation in plasmid and phage recombinants. *Nucleic Acids Res.* 17, 3469–3478. doi:10.1093/nar/17.9.3469.

Zeise, K., and von Tiedemann, A. (2001). Morphological and physiological differentiation among vegetative compatibility groups of *Verticillium dahliae* in relation to *V. longisporum*. *J. Phytopathol.* 149, 469–475. doi:10.1111/j.1439-0434.2001.tb03879.x.

Zuber, S., Carruthers, F., Keel, C., Mattart, A., Blumer, C., Pessi, G., et al. (2003). GacS sensor domains pertinent to the regulation of exoproduct formation and to the biocontrol potential of *Pseudomonas fluorescens* CHA0. *Mol. Plant-Microbe Interact.* 16, 634–644. doi:10.1094/MPMI.2003.16.7.634.
